# The burden of child and maternal malnutrition and trends in its indicators in the states of India: the Global Burden of Disease Study 1990–2017

**DOI:** 10.1016/S2352-4642(19)30273-1

**Published:** 2019-12

**Authors:** India State-Level Disease Burden Initiative Malnutrition Collaborators, Soumya Swaminathan, Rajkumar Hemalatha, Anamika Pandey, Nicholas J Kassebaum, Avula Laxmaiah, Thingnganing Longvah, Rakesh Lodha, Siddarth Ramji, G Anil Kumar, Ashkan Afshin, Subodh S Gupta, Anita Kar, Ajay K Khera, Matthews Mathai, Shally Awasthi, Reeta Rasaily, Chris M Varghese, Anoushka I Millear, Helena Manguerra, William M Gardner, Reed Sorenson, Mari J Sankar, Manorama Purwar, Melissa Furtado, Priyanka G Bansal, Ryan Barber, Joy K Chakma, Julian Chalek, Supriya Dwivedi, Nancy Fullman, Brahmam N Ginnela, Scott D Glenn, William Godwin, Zaozianlungliu Gonmei, Rachita Gupta, Suparna G Jerath, Rajni Kant, Varsha Krish, Rachakulla H Kumar, Laishram Ladusingh, Indrapal I Meshram, Parul Mutreja, Balakrishna Nagalla, Arlappa Nimmathota, Christopher M Odell, Helen E Olsen, Ashalata Pati, Brandon Pickering, Kankipati V Radhakrishna, Neena Raina, Zane Rankin, Deepika Saraf, R S Sharma, Anju Sinha, Bhaskar Varanasi, Chander Shekhar, Hendrik J Bekedam, K Srinath Reddy, Stephen S Lim, Simon I Hay, Rakhi Dandona, Christopher J L Murray, G S Toteja, Lalit Dandona

## Abstract

**Background:**

Malnutrition is a major contributor to disease burden in India. To inform subnational action, we aimed to assess the disease burden due to malnutrition and the trends in its indicators in every state of India in relation to Indian and global nutrition targets.

**Methods:**

We analysed the disease burden attributable to child and maternal malnutrition, and the trends in the malnutrition indicators from 1990 to 2017 in every state of India using all accessible data from multiple sources, as part of Global Burden of Diseases, Injuries, and Risk Factors Study (GBD) 2017. The states were categorised into three groups using their Socio-demographic Index (SDI) calculated by GBD on the basis of per capita income, mean education, and fertility rate in women younger than 25 years. We projected the prevalence of malnutrition indicators for the states of India up to 2030 on the basis of the 1990–2017 trends for comparison with India National Nutrition Mission (NNM) 2022 and WHO and UNICEF 2030 targets.

**Findings:**

Malnutrition was the predominant risk factor for death in children younger than 5 years of age in every state of India in 2017, accounting for 68·2% (95% UI 65·8–70·7) of the total under-5 deaths, and the leading risk factor for health loss for all ages, responsible for 17·3% (16·3–18·2) of the total disability-adjusted life years (DALYs). The malnutrition DALY rate was much higher in the low SDI than in the middle SDI and high SDI state groups. This rate varied 6·8 times between the states in 2017, and was highest in the states of Uttar Pradesh, Bihar, Assam, and Rajasthan. The prevalence of low birthweight in India in 2017 was 21·4% (20·8–21·9), child stunting 39·3% (38·7–40·1), child wasting 15·7% (15·6–15·9), child underweight 32·7% (32·3–33·1), anaemia in children 59·7% (56·2–63·8), anaemia in women 15–49 years of age 54·4% (53·7–55·2), exclusive breastfeeding 53·3% (51·5–54·9), and child overweight 11·5% (8·5–14·9). If the trends estimated up to 2017 for the indicators in the NNM 2022 continue in India, there would be 8·9% excess prevalence for low birthweight, 9·6% for stunting, 4·8% for underweight, 11·7% for anaemia in children, and 13·8% for anaemia in women relative to the 2022 targets. For the additional indicators in the WHO and UNICEF 2030 targets, the trends up to 2017 would lead to 10·4% excess prevalence for wasting, 14·5% excess prevalence for overweight, and 10·7% less exclusive breastfeeding in 2030. The prevalence of malnutrition indicators, their rates of improvement, and the gaps between projected prevalence and targets vary substantially between the states.

**Interpretation:**

Malnutrition continues to be the leading risk factor for disease burden in India. It is encouraging that India has set ambitious targets to reduce malnutrition through NNM. The trends up to 2017 indicate that substantially higher rates of improvement will be needed for all malnutrition indicators in most states to achieve the Indian 2022 and the global 2030 targets. The state-specific findings in this report indicate the effort needed in each state, which will be useful in tracking and motivating further progress. Similar subnational analyses might be useful for other low-income and middle-income countries.

**Funding:**

Bill & Melinda Gates Foundation; Indian Council of Medical Research, Department of Health Research, Ministry of Health and Family Welfare, Government of India.

## Introduction

Malnutrition is a major contributor to disease burden, with more than half of global deaths in children younger than 5 years of age attributable to undernutrition, the vast majority of which are in low-income and middle-income countries, including India.^1^, ^2^, ^3^, ^4^, ^5^ However, overweight among children is also increasing globally, including in Africa and Asia.^3^, ^6^ Addressing the challenge of malnutrition in children and women is essential to ensure optimal cognitive growth and development and overall health and productivity.^7^

Addressing the global burden of malnutrition is a major priority.^8^ To spur action and monitor progress, WHO Global Nutrition Targets were established for six malnutrition indicators to be achieved by 2025.^9^, ^10^ The UN Sustainable Development Goals (SDGs) also set targets with the aim of eliminating malnutrition by 2030.^11^ To strengthen the joint efforts towards reducing malnutrition worldwide, 2016–25 was declared, by the UN, as the Decade of Action on Nutrition.^12^ A WHO and UNICEF review in 2018 suggested that the SDG goal of eliminating all forms of malnutrition by 2030 was aspirational but not achievable and, on the basis of trends so far, recommended targets for the malnutrition indicators up to 2030.^13^

**Research in context**

**Evidence before this study**

Existing evidence suggests that India, with a population of 1·4 billion people residing across states at varying levels of health transition, has a large and persistent burden of malnutrition, especially among children and women of reproductive age. We searched PubMed for published literature on malnutrition in India, Google for reports in the public domain, and references in these papers and reports, using the search terms “anaemia”, “breastfeeding”, “burden”, “child growth failure”, “child obesity”, “child overweight”, “DALY”, “death”, “epidemiology”, “global nutrition targets”, “India”, “infant”, “low birthweight”, “malnutrition”, “morbidity”, “mortality”, “national nutrition mission”, “neonate”, “prevalence”, “stunting”, “sustainable development goals”, “under-five”, “undernutrition”, “underweight”, and “wasting” on April 4, 2019, without language or publication date restrictions. We found several previous studies that have estimated subnational variations in malnutrition burden in India and its association with health outcomes, mainly using single data sources. However, a comprehensive understanding of the variations between the states of India in the prevalence of each malnutrition indicator, the associated deaths and disease burden, and its progress towards achieving the Indian and the global nutrition targets, using all available data sources in a single framework has not been compiled to inform relevant policy interventions suitable for the situation in each state.

**Added value of this study**

This study provides a comprehensive account of the burden of child and maternal malnutrition in every state of India from 1990 to 2017, by use of all available and accessible data that were analysed in the unified Global Burden of Diseases, Injuries, and Risk Factors Study framework. The findings highlight that, even with the many efforts to reduce malnutrition in India, it remains the predominant risk factor for deaths and disease burden in children younger than 5 years and the leading risk factor for disease burden in all ages combined. This study compares the projected prevalence of the malnutrition indicators in each state based on the trends up to 2017, with the targets set by the India National Nutrition Mission for 2022 and WHO and UNICEF for 2030. The substantial gaps between the trends and targets estimated in this report for most states of India indicate that progress toward all malnutrition indicators needs to be accelerated. These gaps vary between the states, indicating the extent of additional effort needed to control malnutrition in each state. The findings highlight that the modest rate of improvement in low birthweight, which is the biggest contributor among the malnutrition indicators to deaths and disease burden in children younger than 5 years of age, should be addressed through focused policy action. Besides the substantial continuing burden of poor nutrition in India, this study also reports that child overweight is increasing rapidly across all states of India.

**Implications of all available evidence**

Malnutrition remains one of the most serious public health challenges across India, although substantial heterogeneity exists between the states for the various malnutrition indicators and their trends over time. The resurgence in policy interest in India to reduce malnutrition across the country through the National Nutrition Mission is encouraging. This momentum can benefit from the use of state-level trends in this study, which highlight the extent of effort needed in each state to achieve the national and the global targets for the various malnutrition indicators.

Decades of policy and programmatic efforts have been made in India to tackle the continuing challenge of malnutrition. In 2017, India released the National Nutrition Strategy, which outlined measures to address malnutrition across the life cycle.^14^ In early 2018, the Prime Minister of India launched the National Nutrition Mission (NNM), also known as POSHAN Abhiyaan, to bring focus and momentum to this effort, which has the overarching goal of reducing child and maternal malnutrition.^15^, ^16^

India had a population of 1·38 billion in 2017, spread across 29 states and seven union territories, which are at varying levels of development, leading to a heterogeneous distribution of health risks and their effects.^17^ The India State-Level Disease Burden Initiative has reported a varied epidemiological transition across the states of India since 1990 as part of the Global Burden of Diseases, Injuries, and Risk Factors Study (GBD).^17^, ^18^ Some subnational studies in India have reported the trends in one or more malnutrition indicators,^19^, ^20^, ^21^, ^22^, ^23^ and some from other countries have reported trends in malnutrition burden^24^, ^25^, ^26^, ^27^, ^28^ or trends in child growth failure indicators.^29^, ^30^ However, there has been no comprehensive consolidation of the malnutrition burden and the trends in all major malnutrition indicators in all states of any country using all available data sources that also relates the projected subnational trends with the policy targets for 2022 and 2030. In this report, we present consolidated findings for each state in India from 1990 to 2017 and compare these trends with Indian and global targets up to 2030 to inform state-specific policy action.

## Methods

### Overview

The analysis and findings of child and maternal malnutrition reported in this Article were produced by the India State-Level Disease Burden Initiative as part of GBD 2017. The work of this Initiative has been approved by the Health Ministry Screening Committee at the Indian Council of Medical Research and the ethics committee of the Public Health Foundation of India. A comprehensive description of the metrics, data sources, and statistical modelling for GBD 2017 has been reported elsewhere.^5^, ^17^, ^18^ The GBD 2017 methods relevant for this paper are summarised here and described in detail in the appendix (pp 3–26).

### Estimation of exposure to malnutrition

The GBD comparative risk assessment framework was used to estimate malnutrition exposure and attributable disease burden. The components of child and maternal malnutrition in GBD are described in the appendix (p 5). All accessible data sources from India were used, including national household surveys, a variety of dietary and nutrition surveys, and other epidemiological studies (appendix pp 25–37). The modelling approaches integrated multiple data inputs, using Spatiotemporal Gaussian process regression, and borrowed information across age, time, and location to produce the best possible estimates of risk exposure by location, age, sex, and year.

For the purpose of reporting the prevalence of the eight malnutrition indicators included in the India NNM target 2022 and the WHO and UNICEF target 2030,^13^, ^31^ the following definitions were used: low birthweight as less than 2500 g; stunting, wasting, and underweight in children younger than 5 years as height-for-age, weight-for-height, and weight-for-age below two SDs of the median in the WHO 2006 standard curve; anaemia in children younger than 5 years as haemoglobin less than 110 g/L; anaemia in women 15–49 years of age as haemoglobin less than 110 g/L if pregnant and 120 g/L if not pregnant; exclusive breastfeeding as no oral food or fluid intake during the first 6 months of life except breast milk and oral rehydration solution drops or syrups containing vitamins, minerals or medicines;^32^ and overweight in children aged 2–4 years as body-mass index above the monthly cutoff for normal weight as reported in the International Obesity Task Force tables.^5^, ^33^

### Estimation of deaths and DALYs attributable to malnutrition

Estimation of attributable disease burden included ascertainment of relative risk of disease outcomes for risk exposure-disease outcome pairs with sufficient evidence of a causal relationship in randomised controlled trials, prospective cohort studies, or case-control studies, as assessed with an approach similar to the World Cancer Research Fund grading system.^5^ Population attributable fractions were estimated from risk exposure, relative risks of outcomes due to exposures, and the theoretical minimum risk exposure (lowest level of risk exposure, below which its relation with a disease outcome is not supported by available evidence) for each malnutrition indicator as explained in the appendix (pp 3–24). Population attributable fractions were used to produce estimates of deaths and disability-adjusted life-years (DALYs) attributable to each malnutrition risk factor by location, age, sex, and year. DALYs are the summary measure of years of healthy life lost due to disability (YLDs) and years of life lost due to premature mortality (YLLs). The major data inputs included vital registration, verbal autopsy studies, large population-level surveys, surveillance data, and hospital-based and community-based studies (appendix pp 25–37).

GBD uses covariates, which are explanatory variables that have a known association with the outcome of interest, to arrive at the best possible estimate when data for the outcome are scarce but data for covariates are available.^5^, ^34^ This approach was part of the estimation process for the findings reported.

### Analysis presented in this paper

We report findings for 31 geographical units in India: 29 states, Union Territory of Delhi, and the union territories other than Delhi (combining the six smaller union territories of Andaman and Nicobar Islands, Chandigarh, Dadra and Nagar Haveli, Daman and Diu, Lakshadweep, and Puducherry). The state of Jammu and Kashmir was divided into two union territories in August, 2019. Because we are reporting findings up to 2017, we report findings for the state of Jammu and Kashmir. We also present findings for three groups of states categorised on the basis of their Socio-demographic Index (SDI) as calculated by GBD.^35^ SDI is a composite indicator of development status, which ranges from 0 to 1, and is a geometric mean of the values of the indices of lag-distributed per capita income, mean education for those 15 years of age or older, and total fertility rate in people younger than 25 years. We assessed the relationship of each malnutrition indicator with the SDI value of the states in 2017. The states were categorised into the three state groups on the basis of their SDI in 2017: low SDI (≤0·53), middle SDI (0·54–0·60), and high SDI (>0·60; appendix p 38).^36^

We assess the rates and proportion of deaths and DALYs attributable to child and maternal malnutrition among children younger than 5 years and DALYs attributable to child and maternal malnutrition among all ages in every state of India in 2017, and compare them with other risk factor categories. We also report cause-specific DALYs in children younger than 5 years attributable to malnutrition and its components in India in 2017. We present the prevalence of the eight malnutrition indicators included in Indian and global targets in the states of India. The targets set by the NNM 2022 and the WHO and UNICEF 2030 are summarised in the panel. We applied these targets to each state of India.

Panel **Targets set by the National Nutrition Mission for 2022 and WHO and UNICEF for 2030**

**National Nutrition Mission 2022 targets**^15^, ^16^

• Low birthweight: 2 percentage point reduction in prevalence annually from 2017 to 2022
• Child stunting*: prevalence of 25% in 2022
• Child underweight*: 2 percentage point reduction in prevalence annually from 2017 to 2022
• Anaemia†: 3 percentage point reduction in prevalence annually in children younger than 5 years and in women 15–49 years of age from 2017 to 2022

**WHO and UNICEF 2030 targets**^13^

• Low birthweight: 30% reduction in prevalence from 2012 to 2030
• Child stunting‡: 50% reduction in number of children younger than 5 years of age who are stunted from 2012 to 2030
• Child wasting: prevalence of less than 3% by 2030
• Anaemia: 50% reduction in prevalence in women 15–49 years of age from 2012 to 2030
• Breastfeeding: prevalence of exclusive breastfeeding in the first 6 months of at least 70% by 2030
• Child overweight: prevalence of less than 3% by 2030

We estimated the annualised percentage change in mid-year estimates of the prevalence of malnutrition indicators for the state SDI groups for three periods: 1990–2000, 2000–10, and 2010–17, and compared the annualised percentage change during 2010–17 with the annualised reduction needed to meet the NNM 2022 and the WHO and UNICEF 2030 targets in each state of India.

We projected the prevalence of malnutrition indicators for India and each state up to 2030 on the basis of the trends from 1990 to 2017. The annualised change for the projections for 2018–30 was calculated using a weight function that gave higher weight to the more recent trends in each state. The detailed methods used for these projections, including the out-of-sample predictive validity test, are described in the appendix (p 23) and elsewhere.^37^

We report estimates with 95% uncertainty intervals (UIs) where relevant. The UIs were based on 1000 runs of the models for each quantity of interest, which have been found to be adequate for the GBD models (appendix p 23 and pp 44–49).^5^ The mean of these distributions was regarded as the point estimate, and the 2·5th and 97·5th percentiles were considered the 95% UI.

### Role of the funding source

Some staff of the Indian Council of Medical Research are co-authors on this paper, having contributed to various aspects of the study and analysis. The other funder of the study had no role in the study design, data collection, data analysis, data interpretation, or writing of this paper. The corresponding author had full access to all of the data in the study and had final responsibility for the decision to submit for publication.

## Results

### Malnutrition burden

Of the 1·04 million under-5 deaths in India in 2017, 706 000 (95% UI 659 000–759 000; 68·2%, 65·8–70·7) could be attributed to malnutrition.^38^ Although all-cause under-5 death rate in India decreased from 2336 per 100 000 (2271–2405) in 1990 to 801 per 100 000 (759–850) in 2017, the proportion of under-5 deaths attributable to malnutrition changed only modestly from 70·4% (67·0–74·0) in 1990 to 68·2% (65·8–70·7) in 2017.^38^ Similarly, the DALY rate attributable to malnutrition in children younger than 5 years reduced by 65·8% (62·9–68·7) from 147 956 per 100 000 (139 350–156 327) in 1990 to 50 627 (47 301–54 199) in 2017, but the proportion of total DALYs in children younger than 5 years attributable to malnutrition changed only slightly from 70·1% (66·8–70·6) in 1990 to 67·1% (64·9–69·4) in 2017, making it the predominant risk factor for health loss (appendix p 39). The vast majority of the malnutrition DALYs in children younger than 5 years in 2017 were due to mortality (94·5% of YLLs, 5·5% of YLDs).^38^ Although the relative contribution of child and maternal malnutrition to total DALYs across all ages has declined in India from 36·5% (95% UI 34·5–38·4) in 1990 to 17·3% (16·3–18·2) in 2017, it is still the leading risk factor for health loss (appendix p 39). The population of 1·38 billion in India in 2017 made up 18·1% of the global population, but India had 25·4% of the total global DALYs attributable to child and maternal malnutrition in 2017.^38^

**Figure 1:**
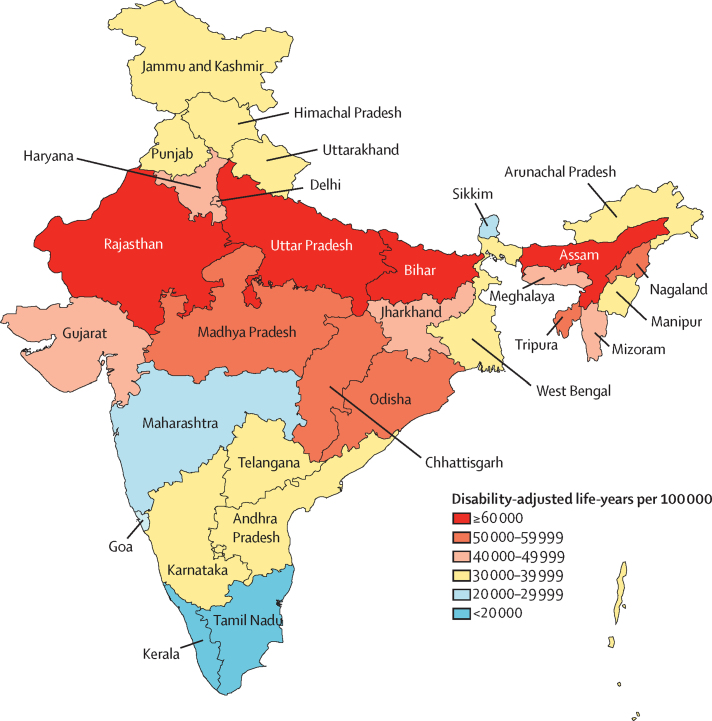
Disability-adjusted life-years rate attributable to malnutrition in children younger than 5 years of age in the states of India, 2017 The state of Jammu and Kashmir was divided into two union territories in August 2019; because we are reporting findings up to 2017, we report findings for the state of Jammu and Kashmir.

Malnutrition was the leading risk factor in children younger than 5 years in every state of India in 2017 (appendix p 39). The DALY rate attributable to malnutrition in children younger than 5 years varied 6·8 times between the states, and it was 1·8 times higher in the low SDI than in the middle SDI state groups and 2·4 times higher than in high SDI state groups (figure 1, appendix p 39). Malnutrition was also the leading risk factor across all ages in 23 states that comprised 64% of India's population in 2017, contributing 10·0%–26·4% of the total DALYs (appendix p 40). The DALY rate attributable to malnutrition across all ages varied 6·0 times between states, and it was 2·0 times higher in the low SDI than in the middle SDI state groups and 2·7 times higher than in high SDI state groups (appendix p 40).

**Figure 2:**
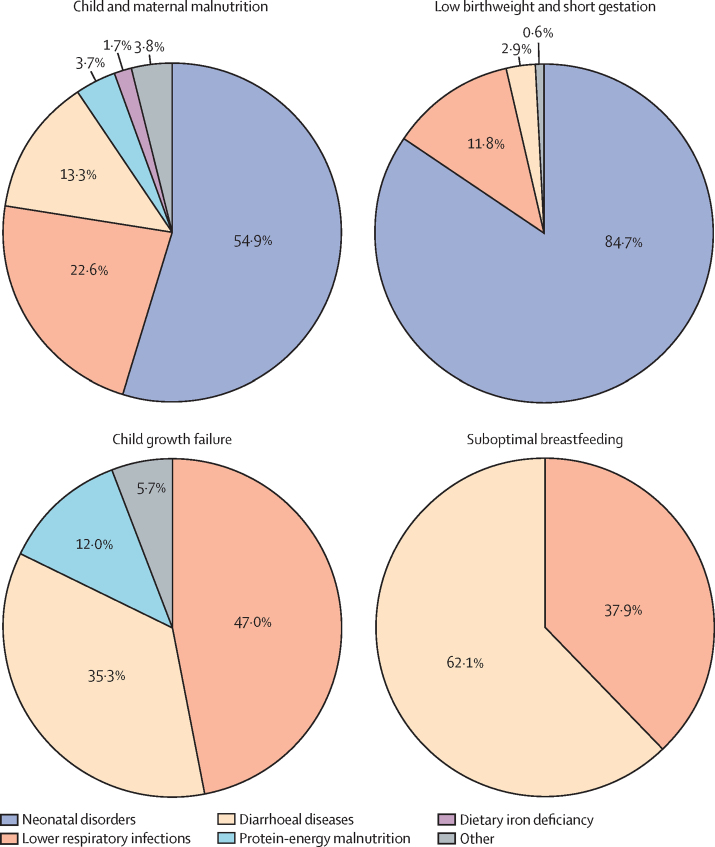
Cause-specific disability-adjusted life-years attributable to malnutrition in children younger than 5 years of age in India, 2017 Data are presented for child and maternal malnutrition and the three leading components. Data shown are percent of total disability-adjusted life-years for each risk that manifests through different diseases. Protein-energy malnutrition is a specific disease cause in Global Burden of Diseases, Injuries, and Risk Factors Study, as opposed to the malnutrition risk factor indicators. For child and maternal malnutrition, the other category includes childhood infections other than diarrhoeal diseases and lower respiratory infections, vitamin A deficiency, and sudden infant death syndrome. For child growth failure, the other category includes measles.

The highest proportion of the malnutrition DALYs in children younger than 5 years in India in 2017 was from low birthweight and short gestation (43·6%, 95% UI 41·8–45·2) followed by child growth failure (20·7%, 19·0–22·5; appendix p 41). Of the total DALYs attributable to malnutrition in children younger than 5 years in India in 2017, the largest proportions were from neonatal disorders (54·9%) followed by lower respiratory infections (22·6%) and diarrhoeal diseases (13·3%; figure 2). The highest proportion of DALYs attributable to low birthweight and short gestation were from neonatal disorders (84·7%; figure 2). The highest proportion of DALYs attributable to child growth failure were from lower respiratory infections (47·0%) followed by diarrhoeal diseases (35·3%; figure 2). The DALYs attributable to suboptimal breastfeeding were from diarrhoeal diseases (62·1%) and lower respiratory infections (37·9%; figure 2).

### Low birthweight

**Figure 3:**
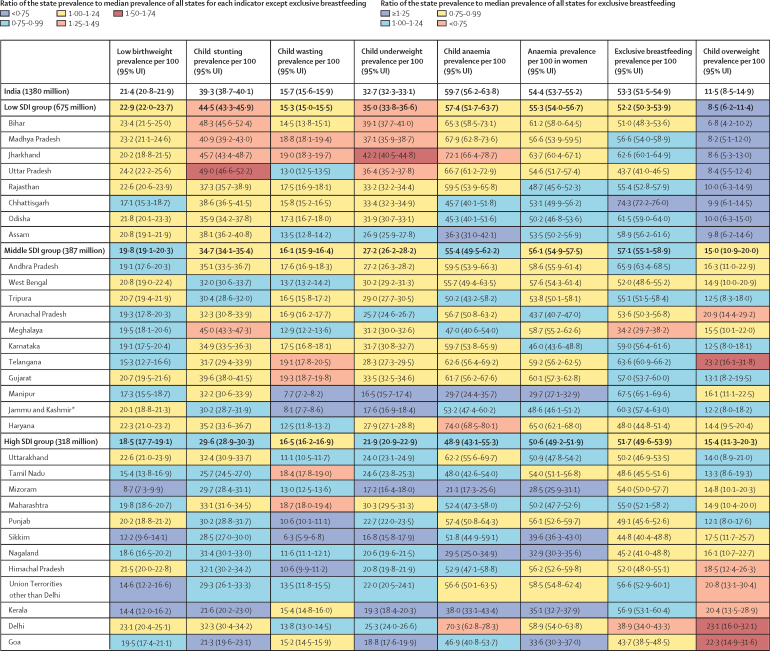
Prevalence of malnutrition indicators in the states of India, 2017 The states are listed in increasing order of Socio-demographic Index in 2017. The population of each state SDI group in 2017 is shown in parentheses. UI=uncertainty interval. SDI=Socio-demographic Index. *The state of Jammu and Kashmir was divided into two union territories in August, 2019; because we are reporting findings up to 2017, we report findings for the state of Jammu and Kashmir.

**Figure 4:**
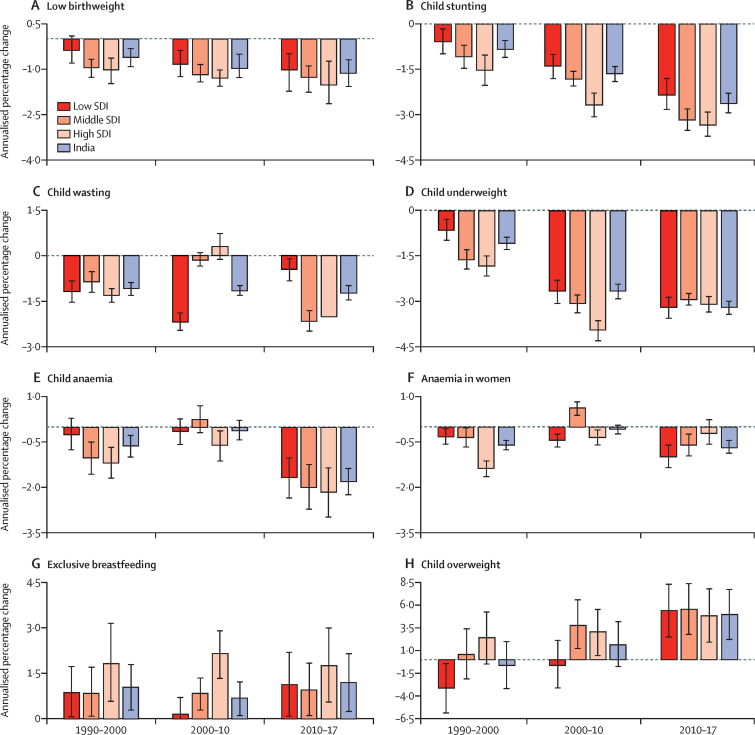
Annualised percentage change in mid-year estimates of the prevalence of malnutrition indicators in the states of India grouped by SDI, 1990–2000, 2000–10, and 2010–17 (A) Low birthweight. (B) Child stunting. (C) Child wasting. (D) Child underweight. (E) Child anaemia. (F) Anaemia in women. (G) Exclusive breastfeeding. (H) Child overweight. Error bars represent 95% uncertainty intervals. SDI=Socio-demographic Index.

**Figure 5:**
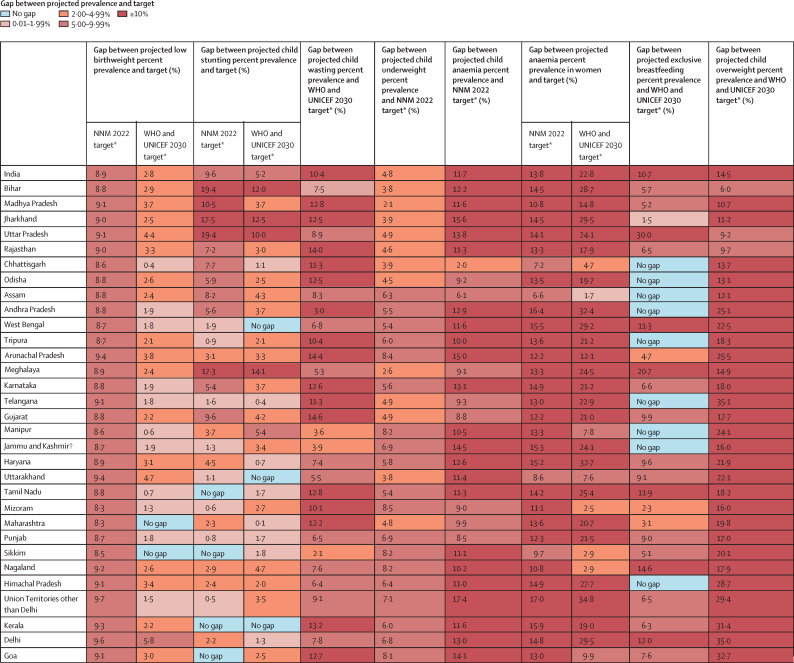
Gap between projected prevalence of malnutrition indicators and the National Nutrition Mission 2022 and the WHO and UNICEF 2030 targets in the states of India The states are listed in increasing order of Socio-demographic Index in 2017. NNM=National Nutrition Mission. *In 2022 and 2030 if trends up to 2017 continue. †The state of Jammu and Kashmir was divided into two union territories in August, 2019; because we are reporting findings up to 2017, we report findings for the state of Jammu and Kashmir.

The prevalence of low birthweight in India was 21·4% (95% UI 20·8–21·9) in 2017. This prevalence decreased moderately with increasing SDI of states (*r*=–0·38, p=0·034), and varied 2·8 times between the states (figure 3). Low birthweight prevalence decreased modestly in India in all the three periods, with relatively higher decline during 2010–17 (1·12% annualised, 95% UI 0·68–1·57; figure 4A; table). The point estimate of annualised percentage reduction was highest in the high SDI state group, with the magnitude of reduction increasing over the three periods across the SDI groups (figure 4A; table). Low birthweight prevalence decreased significantly in 14 states of India during 2010–17 (range 1·10%–3·76% annualised) but was much lower than the 11·8% annualised reduction needed for the NNM 2022 target (table). None of the states except Sikkim had the annualised reduction of 2·3% needed for the WHO and UNICEF 2030 target. The projected prevalence, based on trends between 1990 and 2017, of 20·3% in 2022 was 2·9% more than the NNM target of 11·4%, and the projected prevalence of 18·7% in 2030 was 11·4% more than the WHO and UNICEF target of 15·8% (figure 5; appendix pp 42–43). The projected prevalence of low birthweight was higher than the target prevalence in 2022 for all states and in 2030 for all states except Sikkim and Maharashtra (figure 5; appendix pp 42–43).

**Table:** Annualised percentage change in the prevalence of malnutrition indicators in the states of India, 2010–17

		**Low birthweight (95% UI)**	**Child stunting (95% UI)**	**Child wasting (95% UI)**	**Child underweight (95% UI)**	**Child anaemia (95% UI)**	**Anaemia in women (95% UI)**	**Exclusive breastfeeding (95% UI)**	**Child overweight (95% UI)**
India		−1·12% (−1·57 to −0·68)	−2·63% (−2·94 to −2·27)	−1·23% (−1·47 to −0·97)	−3·22% (−3·44 to −2·98)	−1·81% (−2·26 to −1·36)	−0·68% (−0·89 to −0·44)	1·19% (0·22 to 2·16)	4·98% (2·18 to 7·78)
Low SDI		−1·03% (−1·73 to −0·48)	−2·34% (−2·83 to −1·81)	−0·44% (−0·85 to −0·07)	−3·41% (−3·77 to −3·04)	−1·66% (−2·35 to −1·00)	−0·98% (−1·35 to −0·60)	1·13% (0·08 to 2·21)	5·43% (2·48 to 8·39)
	Bihar	−1·27% (−2·62 to 0·13)	−1·82% (−2·97 to −0·38)	−1·69% (−2·76 to −0·68)	−3·42% (−4·36 to −2·54)	−1·07% (−2·84 to 0·84)	−0·36% (−1·22 to 0·45)	2·58% (0·99 to 4·38)	5·04% (0·94 to 9·03)
	Madhya Pradesh	−0·94% (−2·10 to 0·28)	−2·93% (−3·70 to −2·10)	−1·71% (−2·53 to −0·94)	−4·78% (−5·76 to −3·88)	−1·83% (−3·20 to −0·53)	−1·75% (−2·51 to −0·99)	2·66% (1·16 to 4·35)	7·21% (3·35 to 11·24)
	Jharkhand	−1·28% (−2·29 to −0·34)	−1·63% (−2·59 to −0·56)	−0·30% (−1·13 to 0·55)	−3·15% (−4·20 to −2·19)	−0·88% (−2·33 to 0·73)	−0·46% (−1·21 to 0·29)	0·87% (−0·13 to 1·88)	5·98% (1·73 to 9·99)
	Uttar Pradesh	−0·77% (−2·21 to 0·77)	−2·02% (−3·02 to −0·88)	0·55% (−0·37 to 1·44)	−3·03% (−3·76 to −2·36)	−0·88% (−2·17 to 0·43)	−0·53% (−1·35 to 0·38)	−0·47% (−2·04 to 1·17)	5·08% (1·31 to 9·01)
	Rajasthan	−1·05% (−2·05 to 0·12)	−3·03% (−3·69 to −2·33)	0·84% (−0·02 to 1·71)	−3·31% (−3·86 to −2·77)	−1·37% (−3·02 to 0·13)	−1·44% (−2·33 to −0·52)	2·22% (0·73 to 3·95)	4·36% (0·39 to 8·25)
	Chhattisgarh	−2·09% (−3·82 to −0·56)	−3·51% (−4·63 to −2·27)	0·23% (−0·73 to 1·24)	−3·48% (−4·29 to −2·79)	−4·43% (−6·50 to −2·28)	−1·91% (−2·86 to −0·90)	0·28% (−0·23 to 0·84)	6·32% (2·44 to 10·22)
	Odisha	−1·15% (−2·18 to 0·04)	−3·15% (−3·97 to −2·23)	−1·14% (−1·98 to −0·26)	−2·94% (−3·68 to −2·32)	−5·03% (−7·09 to −3·02)	−0·98% (−2·15 to 0·13)	1·40% (0·32 to 2·56)	6·03% (1·84 to 9·94)
	Assam	−1·27% (−2·50 to −0·10)	−2·73% (−3·80 to −1·60)	−1·06% (−2·24 to 0·08)	−2·61% (−3·20 to −2·08)	−7·27% (−9·55 to −4·84)	−2·78% (−3·99 to −1·76)	0·87% (−0·15 to 2·00)	4·94% (1·01 to 9·05)
Middle SDI		−1·28% (−1·75 to −0·88)	−3·18% (−3·51 to −2·82)	−2·16% (−2·51 to −1·81)	−2·93% (−3·15 to −2·72)	−1·99% (−2·73 to −1·23)	−0·61% (−0·97 to −0·22)	0·94% (0·09 to 1·85)	5·58% (2·73 to 8·46)
	Andhra Pradesh	−1·29% (−2·30 to 0·03)	−2·87% (−3·66 to −2·12)	−1·34% (−2·20 to −0·44)	−2·69% (−3·20 to −2·21)	−1·79% (−3·71 to 0·20)	−0·17% (−1·02 to 0·59)	0·52% (−0·27 to 1·40)	5·54% (1·61 to 9·57)
	West Bengal	−1·45% (−2·72 to −0·39)	−3·92% (−4·81 to −3·07)	−3·51% (−4·37 to −2·56)	−2·67% (−3·18 to −2·16)	−1·66% (−3·90 to 0·52)	−0·33% (−1·29 to 0·73)	0·60% (−0·81 to 1·99)	5·89% (2·18 to 9·55)
	Tripura	−1·37% (−2·44 to −0·46)	−2·89% (−3·81 to −1·94)	−1·94% (−2·98 to −0·92)	−2·73% (−3·48 to −2·01)	−3·06% (−5·55 to −0·84)	−1·20% (−2·39 to 0·08)	2·75% (1·12 to 4·46)	4·29% (0·04 to 7·92)
	Arunachal Pradesh	−0·64% (−1·68 to 0·70)	−2·98% (−3·87 to −2·02)	−0·75% (−1·76 to 0·29)	−1·96% (−2·66 to −1·34)	−1·53% (−3·19 to 0·22)	−1·92% (−3·00 to −0·82)	1·06% (−0·29 to 2·52)	4·06% (0·36 to 7·66)
	Meghalaya	−1·23% (−2·54 to 0·28)	−1·22% (−1·97 to −0·44)	−4·04% (−5·25 to −2·87)	−5·37% (−6·32 to −4·45)	−4·04% (−7·12 to −1·54)	−0·23% (−1·16 to 0·78)	2·88% (−0·15 to 6·28)	2·63% (−1·16 to 6·50)
	Karnataka	−1·27% (−2·39 to −0·43)	−2·94% (−3·65 to −2·27)	−2·09% (−2·95 to −1·23)	−3·17% (−3·61 to −2·75)	−2·18% (−3·88 to −0·54)	−0·85% (−1·77 to 0·06)	0·44% (−0·57 to 1·52)	5·53% (1·70 to 9·40)
	Telangana	−0·99% (−3·06 to 1·13)	−3·63% (−4·67 to −2·64)	−3·07% (−4·05 to −2·17)	−4·20% (−4·75 to −3·66)	−2·17% (−3·95 to −0·43)	−0·91% (−1·89 to 0·06)	1·07% (0·19 to 2·09)	5·38% (1·60 to 9·23)
	Gujarat	−1·32% (−2·27 to −0·34)	−2·86% (−3·74 to −2·01)	−0·47% (−1·15 to 0·23)	−2·45% (−3·03 to −1·92)	−3·16% (−4·50 to −1·87)	−1·09% (−1·82 to −0·32)	1·07% (−0·28 to 2·54)	5·74% (2·03 to 9·57)
	Manipur	−1·84% (−3·49 to −0·82)	−2·29% (−3·13 to −1·50)	−1·93% (−3·35 to −0·60)	−2·88% (−3·55 to −2·20)	−5·52% (−8·87 to −2·46)	−2·74% (−4·29 to −1·05)	0·96% (0·22 to 1·76)	5·66% (1·56 to 9·65)
	Jammu and Kashmir*	−1·36% (−2·35 to −0·03)	−2·51% (−3·37 to −1·67)	−0·41% (−1·67 to 0·86)	−3·18% (−3·79 to −2·55)	−0·81% (−2·60 to 1·16)	−0·53% (−1·44 to 0·40)	1·49% (0·34 to 2·71)	4·84% (0·99 to 8·83)
	Haryana	−1·10% (−1·84 to −0·23)	−3·42% (−4·17 to −2·61)	−3·66% (−4·90 to −2·53)	−3·20% (−3·68 to −2·70)	−0·06% (−1·48 to 1·31)	−0·17% (−1·07 to 0·62)	3·36% (1·25 to 5·67)	6·70% (2·47 to 10·83)
High SDI		−1·52% (−2·16 to −0·74)	−3·33% (−3·72 to −2·92)	−2·01% (−2·46 to −1·58)	−3·11% (−3·36 to −2·84)	−2·16% (−2·98 to −1·33)	−0·21% (−0·60 to 0·25)	1·75% (0·54 to 3·01)	4·83% (1·88 to 7·90)
	Uttarakhand	−0·64% (−1·65 to 0·17)	−3·87% (−4·67 to −3·08)	−1·60% (−2·84 to −0·38)	−4·83% (−5·39 to −4·31)	−0·02% (−1·80 to 1·75)	−1·40% (−2·40 to −0·48)	2·15% (0·33 to 4·06)	7·12% (3·16 to 11·07)
	Tamil Nadu	−1·72% (−3·57 to 0·16)	−3·31% (−4·12 to −2·42)	−1·97% (−2·71 to −1·23)	−3·47% (−3·93 to −3·01)	−2·74% (−4·52 to −0·94)	0·32% (−0·45 to 1·08)	2·04% (0·39 to 3·86)	4·96% (1·06 to 9·05)
	Mizoram	−0·87% (−4·09 to 1·74)	−2·88% (−3·79 to −2·04)	−1·18% (−2·18 to −0·11)	−2·38% (−3·01 to −1·75)	−8·35% (−11·25 to −5·29)	−3·00% (−4·44 to −1·57)	1·91% (0·38 to 3·67)	2·49% (−1·45 to 6·44)
	Maharashtra	−2·14% (−3·04 to −1·07)	−3·26% (−4·03 to −2·50)	−2·67% (−3·44 to −1·92)	−2·84% (−3·28 to −2·39)	−2·47% (−4·12 to −0·88)	−0·69% (−1·54 to 0·29)	1·44% (0·08 to 2·99)	4·17% (0·42 to 8·18)
	Punjab	−1·44% (−2·47 to −0·45)	−3·37% (−4·13 to −2·60)	−1·90% (−3·00 to −0·80)	−3·52% (−3·97 to −3·07)	−1·75% (−3·32 to −0·10)	0·18% (−0·68 to 1·07)	2·15% (0·40 to 4·29)	4·86% (0·76 to 8·92)
	Sikkim	−3·76% (−7·24 to −1·29)	−3·15% (−4·21 to −2·23)	−2·35% (−3·88 to −0·82)	−3·41% (−4·21 to −2·64)	−1·79% (−4·04 to 0·28)	−3·40% (−4·64 to −2·07)	2·80% (0·65 to 5·04)	2·86% (−1·08 to 7·15)
	Nagaland	−1·17% (−2·75 to −0·15)	−2·68% (−3·52 to −1·88)	−1·34% (−2·48 to −0·30)	−2·99% (−3·62 to −2·39)	−6·17% (−9·71 to −2·37)	−3·45% (−4·72 to −2·10)	2·25% (0·05 to 4·67)	2·87% (−0·95 to 6·63)
	Himachal Pradesh	−0·99% (−2·38 to 0·26)	−3·18% (−4·26 to −2·16)	−1·95% (−3·32 to −0·56)	−3·87% (−4·61 to −3·24)	−0·72% (−2·68 to 1·46)	−0·05% (−1·03 to 0·96)	3·81% (1·85 to 6·10)	6·27% (2·56 to 10·21)
	UTs other than Delhi	−1·10% (−3·63 to 1·63)	−2·48% (−3·95 to −1·01)	−0·53% (−1·95 to 0·77)	−1·60% (−3·14 to −0·57)	1·01% (−1·19 to 2·93)	0·42% (−0·57 to 1·54)	1·01% (−0·34 to 2·54)	4·56% (0·74 to 8·29)
	Kerala	−0·84% (−4·11 to 1·51)	−3·94% (−4·92 to −2·95)	−0·27% (−1·22 to 0·66)	−3·89% (−4·49 to −3·29)	−3·68% (−5·93 to −1·42)	−0·58% (−1·85 to 0·74)	0·92% (−0·38 to 2·28)	6·15% (2·45 to 10·14)
	Delhi	−0·31% (−1·76 to 1·16)	−3·29% (−4·29 to −2·37)	−1·99% (−3·31 to −0·77)	−1·85% (−2·50 to −1·21)	0·38% (−1·40 to 2·30)	0·31% (−0·75 to 1·36)	2·83% (0·27 to 5·48)	5·11% (1·49 to 8·87)
	Goa	−0·88% (−2·71 to 0·33)	−2·41% (−3·80 to −1·17)	0·32% (−0·73 to 1·36)	−1·87% (−2·53 to −1·22)	−0·01% (−2·60 to 2·46)	−1·84% (−3·13 to −0·59)	3·97% (1·51 to 6·46)	4·61% (0·63 to 8·43)

The states are listed in increasing order of Socio-demographic Index in 2017. UI=uncertainty interval. SDI=Socio-demographic Index. UTs=Union Territiories.

* The state of Jammu and Kashmir was divided into two union territories in August 2019; as we are reporting findings up to 2017, we report findings for the state of Jammu and Kashmir.

### Child stunting

The prevalence of child stunting was 39·3% (95% UI 38·7–40·1) in India in 2017 (figure 3). This prevalence was inversely correlated with the SDI of the states (*r*=–0·79, p<0·0001), and varied 2·3 times between the states (figure 3). The annualised percentage reduction in stunting prevalence was seen in India in all the three periods, with the highest reduction during 2010–17 (2·63% annualised, 95% UI 2·27–2·94; figure 4B; table). The point estimate for annualised percentage reduction was higher in the high SDI compared with the low SDI state group, with the magnitude of reduction increasing over the three periods in all the SDI groups (figure 4B; table). Stunting prevalence reduced significantly in every state of India during 2010–17 (range 1·22%–3·94% annualised), but this decrease was less than the 8·6% annualised reduction needed for the NNM 2022 target and the 4·2% reduction needed for WHO and UNICEF 2030 target. The projected prevalence of 34·6% in 2022, based on trends between 1990 and 2017, was 9·6% more than the NNM target of 25·0%, and the projected prevalence of 27·7% in 2030 was 5·1% more than the WHO and UNICEF target of 22·6% (figure 5; appendix pp 42–43). The projected prevalence of stunting was higher than the target prevalence for most states of India, except for Tamil Nadu, Sikkim, Kerala, and Goa in 2022 and Uttarakhand, West Bengal, and Kerala in 2030 (figure 5; appendix pp 42–43).

### Child wasting

Within child growth failure, the highest contribution to DALYs was from child wasting (19·0%, 95% UI 16·2–21·2; appendix p 40). The prevalence of child wasting was 15·7% (95% UI 15·6–15·9) in India in 2017. This prevalence did not have a significant correlation with the SDI of states (*r*=–0·30, p=0·097), but had a 3·1 times variation between the states (figure 3). The point estimate of annualised percentage reduction of wasting in India was highest during 2010–17 (1·23%, 95% UI 0·97–1·47), with substantial variation across the state SDI groups during the three periods (figure 4C; table). The annualised percentage decrease was similar across the state SDI groups during 1990–2000, was highest in the low SDI state group during 2000–10, and was higher in the middle and high SDI groups than the low SDI group during 2010–17 (figure 4C; table). Although wasting prevalence significantly declined in many states of India, the reduction was much lower than the 12·0% annualised reduction needed for the WHO and UNICEF 2030 target (table). The projected prevalence fro India of 13·4% in 2030, based on trends between 1990 and 2017, was 10·4% higher than the WHO and UNICEF target of wasting prevalence of less than 3% (figure 5; appendix p 43). No state met these targets.

### Child underweight

The prevalence of child underweight was 32·7% (95% UI 32·3–33·1) in India in 2017. This prevalence was inversely correlated with the SDI of the states (*r*=–0·76, p<0·0001), and varied 2·6 times between the states (figure 3). The annualised percentage reduction in underweight prevalence was seen in India in all the three periods, with higher reductions occurring in the last two periods than in 1990–2000 (figure 4D; table). The point estimate for annualised percentage reduction was higher in the high SDI state group compared with the low SDI group during 1990–2000 and 2000–10 but was higher in the low SDI group compared with the high SDI group during 2010–17 (figure 4D; table). The underweight prevalence reduced significantly in every state of India during 2010–17 (range 1·60%–5·37% annualised), but this decrease was less than the 7·0% annualised reduction needed to achieve the NNM 2022 target. The projected prevalence for India of 27·5% in 2022, based on trends between 1990 and 2017, was 4·8% more than the NNM target of 22·7%; this difference varied from 2·1% to 8·5% across the states (figure 5; appendix p 42).

### Child anaemia

The prevalence of child anaemia was 59·7% (95% UI 56·2–63·8) in India in 2017. This prevalence did not have a significant correlation with the SDI of the states (*r*=–0·25, p=0·17), but had a 3·5 times variation between the states (figure 3). The annualised percentage prevalence of child anaemia decreased in India during 2010–17 (1·81%, 95% UI 1·36–2·26), with no significant change during 2000–10 (figure 4E; table). The estimate of child anaemia prevalence decreased significantly in the high SDI state group during 2000–10 and decreased in all SDI groups during 2010–17. Although the prevalence of child anaemia decreased significantly in 16 states of India during 2010–17 (range 1·75%–8·35% annualised), none of these states, except Assam, Mizoram, and Nagaland, had the annualised reduction of 5·6% needed to achieve the NNM 2022 target (table). The projected prevalence of 56·4% in India in 2022, based on trends between 1990 and 2017, was 11·7% higher than the NNM target of 44·7%; this difference was more than 10% for most of the states (figure 5; appendix p 42).

### Anaemia in women

The prevalence of anaemia in women 15–49 years of age was 54·4% (95% UI 53·7–55·2) in India in 2017. This prevalence was inversely correlated with the SDI of the states (*r*=–0·40, p=0·027), and varied 2·3 times between the states (figure 3). The annualised percentage of anaemia prevalence decreased in India during 2010–17 (0·68%, 95% UI 0·44–0·89), with no change during 2000–10 (figure 4F; table). The point estimate of anaemia prevalence decreased in all the SDI state groups in all the three periods, except for the middle SDI group during 2000–10. The annualised percentage decrease was highest in the high SDI state group during 1990–2000, and in the low and middle SDI groups during 2010–17 (figure 4F; table). The prevalence of anaemia decreased significantly in 12 states of India during 2010–17 (range 1·09%–3·45% annualised), but none of the states had the annualised reduction of 6·2% needed to achieve the NNM 2022 target and 4·9% for the WHO and UNICEF 2030 target (table). The projected prevalence of 53·2% in 2022, based on trends between 1990 and 2017, was 13·8% higher than the NNM target of 39·4%, and the projected prevalence of 51·1% in 2030 was 22·8% higher than the WHO and UNICEF target of 28·3%; these gaps varied substantially across the states of India (figure 5; appendix pp 42–43).

### Exclusive breastfeeding

The prevalence of exclusive breastfeeding was 53·3% (95% UI 51·5–54·9) in India in 2017, with a moderate inverse correlation with the SDI of the states (*r*=–0·38, p=0·036). This prevalence varied 2·2 times between the states (figure 3). The annualised percentage increase in the prevalence of exclusive breastfeeding in India during 2010–17 (1·19%, 95% UI 0·22–2·16) was similar to 1990–2000 (1·04%, 0·26–1·82; figure 4G; table). Except for low SDI state group during 2000–10, the prevalence of exclusive breastfeeding increased in all the SDI groups in all the three periods, with relatively higher increase in the high SDI group (figure 4G; table). However, based on the modest increasing trends between 1990 and 2017, the projected prevalence for India was 59·3%, 10·7% less than the WHO and UNICEF 2030 target of at least 70%; only a few states met this target (figure 5; appendix p 43).

### Child overweight

The prevalence of overweight in children aged 2–4 years was 11·5% (95% UI 8·5–14·9) in India in 2017. This prevalence was positively correlated with the SDI of the states (*r*=0·79, p<0·0001), with 3·4 times variation between the states. The prevalence of child overweight increased significantly in India during 2010–17 (4·98%, 95% UI 2·18–7·78), with similar annualised percentage increase in the three state SDI groups (figure 4H; table). Significant annualised percentage increase occurred in the middle SDI and high SDI state groups during 2000–10 also. The projected child overweight prevalence of 17·5% in India in 2030, based on trends between 1990 and 2017, was 14·5% higher than the WHO and UNICEF 2030 target of less than 3% (figure 5; appendix p 43), and no state met these targets.

## Discussion

The findings in this report provide insights into the trends in child and maternal malnutrition burden and the key indicators that can inform further efforts to reduce this burden for every state of India. Although the burden of child and maternal malnutrition has declined in India since 1990, it remains the predominant risk factor for health loss in children younger than 5 years of age in every state of the country and the leading risk factor for health loss across all ages in the majority of states. The malnutrition DALY rate is highest in the low SDI states, with substantial variation between the states. The malnutrition DALYs in children younger than 5 years of age are predominantly due to premature mortality.

Low birthweight, the largest contributor to the malnutrition DALYs in India, had a prevalence of 21% in 2017, which showed a modest declining trend. Within child growth failure, the highest contribution to DALYs was from wasting, the prevalence of which declined only moderately in India during 2010–17. The prevalence of stunting and underweight has been decreasing, however, the prevalence has remained very high in India at 39% and 33%, respectively, in 2017. The prevalence of anaemia has been extremely high in India at 60% in children and 54% in women in 2017, with only moderate decline during 2010–17. However, the prevalence of child overweight has increased considerably in India in the past decade, with a prevalence of 12% in 2017. The prevalence of exclusive breastfeeding was 53% in India in 2017, with a moderate increase during 2010–17. Substantial state-level variations exist in the prevalence for each of the malnutrition indicators. The findings in this report indicate that, if the trends up to 2017 continue, the NNM 2022 and the WHO and UNICEF 2030 targets will not be achieved in most states of India, except for low birthweight and stunting in a few states and exclusive breastfeeding in several.

Because low birthweight was the largest contributor to child malnutrition DALYs in India, its slow decline should be addressed as a priority. South Asia, with India as its largest component, is estimated to have the highest prevalence of low birthweight for any region in the world.^39^ A major issue with tracking low birthweight is the poor quality of birthweight data in many low-income and middle-income countries, including India.^39^ Low birthweight adversely affects not only child health but also increases the risk of chronic diseases later in life.^40^, ^41^, ^42^, ^43^, ^44^, ^45^, ^46^, ^47^ Weight at birth is an intergenerational issue dependent on an interplay of various factors, including maternal undernutrition, intrauterine growth, gestation at birth, birth spacing and order, and maternal age. The higher proportion of underweight women in the reproductive age group in India compared with sub-Saharan Africa has been suggested to contribute to a higher prevalence of low birthweight in India, even though sub-Saharan Africa is poorer.^48^ Chronic energy deficiency in women of reproductive age is a manifestation of long-standing malnutrition reported to be common in India, which increases the risk of preterm births and infants with low birthweight.^1^, ^49^, ^50^, ^51^ Improving the nutritional status of girls in general and that of women in the preconception period and during pregnancy and provision of quality antenatal care, including the treatment of pregnancy complications, would positively affect low birthweight and extend the benefits to the next generation.^39^, ^52^, ^53^, ^54^ Aligned with the Global Every Newborn Action Plan, the India Newborn Action Plan launched in 2014 aims to reduce low birthweight through improved preconception and antenatal care, adolescent-specific health services, nutritional counselling, and micronutrient supplementation.^46^, ^55^

India has been trying to address child malnutrition for many decades through various policy initiatives, such as the Integrated Child Development Scheme launched in 1975, the National Nutrition Policy 1993, the Mid Day Meal Scheme for school children 1995, and the National Food Security Act 2013,^56^, ^57^ but the prevalence of stunting, wasting and underweight remains high. The prevalence of stunting, an indicator of chronic undernutrition, caused by a variety of social, environmental, and economic risk factors, is unsurprisingly highest in the less developed states. However, the prevalence of wasting, indicative of acute undernutrition, is highest in some of the more developed states. The decline in stunting is usually accompanied by a temporary increase or stagnancy in wasting; therefore, achieving a simultaneous reduction of stunting and wasting might be difficult.^58^ Women's status, birth order, son preference, and open defecation contribute to relatively higher rates of undernutrition among children in South Asian countries, including India, compared with sub-Saharan African countries with comparable or lower incomes.^59^, ^60^, ^61^ Alongside the nutrient-based interventions, more comprehensive and inclusive policies addressing all of the key determinants of child malnutrition are needed to accelerate reduction of child growth failure in India as also envisioned in the NNM.^15^

The high burden of anaemia in children and women, with only a modest decline since 1990, is a major public health issue in India. Anaemia increases the risk of adverse birth outcomes and mortality during and after child birth and leads to poor cognitive and physical development and mortality in children.^1^, ^62^, ^63^, ^64^, ^65^, ^66^ Interventions to improve nutrition of girls, including reduction of the prevalence of anaemia, starting at a young age, are needed for better pregnancy-related and early child health outcomes and for a beneficial long-term effect on future generations.^1^, ^53^, ^54^, ^67^, ^68^ India launched the National Iron Plus Initiative in 2013 to comprehensively address anaemia burden across the life cycle, through age-specific interventions with iron and folic acid supplementation and deworming.^69^ Other initiatives in India to address the developmental needs of adolescents in general, and the nutrition and reproductive health needs of adolescent girls in particular, include the National Adolescent Health Programme 2014 and the Scheme for Adolescent Girls.^70^, ^71^ As emphasised in the recent NNM of India, a set of interventions to optimise the health of adolescents and young women would be more effective than any single intervention addressing macronutrient or micronutrient deficiency.^15^

Many states in India will not be able to meet the WHO and UNICEF 2030 target of 70% exclusive breastfeeding if the slow rate of increase observed up to 2017 continues. Promotion of exclusive breastfeeding is essential to support optimal growth and development of the infant and address the burden of child growth failure and child overweight.^72^, ^73^ The efforts to increase exclusive breastfeeding in India include the Infant and Young Child Feeding Guidelines, government regulation on breast milk substitutes, and operational platforms to deliver interventions, such as the Integrated Child Development Scheme and the National Breastfeeding Promotion Programme.^74^, ^75^, ^76^, ^77^, ^78^ Challenges in further improving the rates of exclusive breastfeeding in India include societal beliefs that encourage mixed feeding practices, inadequate lactation support, and aggressive promotion of breast milk substitutes.^73^

The increasing prevalence of overweight in children in India is of concern, with adverse effects on health during childhood as well as long-term chronic effects persisting into adulthood.^1^ Interventions to reduce the burden of overweight children in India should focus on improving the modifiable risk factors, including appropriate child feeding practices, dietary intake, and physical activity.^79^ The draft of the Food Safety and Standards (Safe and Wholesome Food for School Children) Regulations 2018 is indicative of efforts to promote a balanced diet and reduce the availability of foods high in fat, salt, and sugar to school-aged children in India.^80^ However, a comprehensive approach to addressing child overweight needs to be developed in India.

Substantial improvements across the malnutrition indicators in the states of India would require an integrated nutrition policy to effectively address the broader determinants of undernutrition across the life cycle. These improvements include providing clean drinking water, reducing rates of open defecation, improving women's status, enhancing agricultural productivity and food security, promoting nutrition-sensitive agriculture, coupled with harmonisation of efforts across ministries and sectors, political will and good governance, and strategic investments in a multisectoral approach.^1^, ^59^, ^77^, ^81^, ^82^, ^83^, ^84^, ^85^ The Government of India launched a revamped NNM with a budget of US$1·3 billion to comprehensively address the challenge of persistent undernutrition.^15^, ^16^, ^86^ The goal of this Mission is to systematically synergise a variety of nutrition-related activities of various government ministries and stakeholders in order to strengthen many maternal and child health initiatives across the life cycle. This includes the supplementary nutrition component of Integrated Child Development Scheme, Maternity Benefit Programme, Mid Day Meal Scheme, dietary diversification to improve iron and folic acid intake, engaging the private sector in food fortification efforts, and placing emphasis on the broader social determinants of nutrition. This renewed focus on a multisectoral approach to address malnutrition is encouraging, and the targets set by the Mission could motivate the states to accelerate progress. Additionally, several ongoing initiatives under the Ministry of Women and Child Development to reduce gender inequality and empower women can also contribute to improvements in malnutrition.^87^ The major ongoing sanitation improvement drive in India under the Swachh Bharat Mission is also expected to contribute to the reduction in malnutrition. However, our findings suggest that the malnutrition indicator targets set by NNM for 2022 are aspirational, and the rate of improvement needed to achieve these targets is much higher than the rate observed in this study, which might be difficult to reach in a short period. This slow pace of improvement needs to be accelerated, so that future prevalence of the malnutrition indicators are better than our projections based on trends so far. Just as the WHO and UNICEF 2030 targets were set with the realisation that the SDG target of eliminating all forms of malnutrition by 2030 was not achievable, by use of the trends presented for each state in this report, the NNM could set bold but potentially achievable targets for 2030 for India. Another report has also estimated a substantial gap between the NNM 2022 target for stunting and the projected prevalence for India if the trends between National Family Health Survey 2005–06 and 2015–16 continue.^23^ Generally, low-income and middle-income countries would benefit from setting national and subnational targets for reducing malnutrition that are based on robust analysis of trends.

The general limitations of the GBD methods have been described elsewhere.^5^ Limitations specific to the findings in this report include relatively poor data on low birthweight in India. Birthweight is generally not recorded well or remembered by parents and is incorrectly documented in many instances in India, so relatively less reliable data on this indicator is obtained in household surveys; this situation needs to improve for better estimates of low birthweight.^39^, ^88^ There is also scope for improving the estimates of preterm births in India with more robust data. GBD defines child overweight at age 2–4 years using the International Obesity Task Force standards as more data from various countries are available for these ages, which generally leads to relatively higher child overweight prevalence as compared with the WHO child growth reference data for children younger than 5 years of age used by the WHO global nutrition target.^89^ We used periods to present the rate of change in trends for malnutrition indicators, because they are easy to understand. However, this approach could mask finer trends within these periods. The strengths of the findings in this report include the use of all accessible data sources in India and modelling them for best fits, which reduces the chance of erratic estimates that can be observed in individual surveys with variable data quality.^90^, ^91^, ^92^ The estimates of malnutrition burden and the trends in its indicators for every state of the country over a quarter of a century and their future trajectory produced using the standardised GBD methods, and the comprehensive inputs by leading experts in India on the analysis and interpretation of the findings, are other strengths of the findings presented in this report.

India has had increasing food self-sufficiency and food security with the Green Revolution that started in the late 1960s.^81^, ^93^, ^94^ Even with these improvements, India continues to have a high prevalence of undernutrition, combined with an increasing prevalence of overweight and obesity in a subset of the population. Addressing this persistent development problem requires India to ensure implementation of practical and effective policies and interventions across the life cycle that consider the subnational variations and the context of each state. The focus brought to malnutrition by the National Nutrition Mission effort is likely to build momentum towards more rapid reduction of malnutrition in India. The findings in this report provide a reference for monitoring the progress of malnutrition indicators in the coming years in each state of the country. Robust estimation of malnutrition indicators and their trends over time would also be needed at the district level to understand intra-state variations, especially in the large states of India. Comprehensive subnational assessment of the trends in malnutrition indicators, their projections, and their association with policy targets, as presented in this report, could also be useful in other countries to inform decision making to improve subnational disparities in nutritional status.

**This online publication has been corrected. The corrected version first appeared at thelancet.com/child-adolescent on September 30, 2019**

## Footnotes

* The National Nutrition Mission 2022 target for stunting and underweight is for children aged 0–6 years; for consistency with the global targets we estimated this for children younger than 5 years.

† The National Nutrition Mission 2022 target for child anaemia is for children aged 6–59 months; for consistency with the other targets we estimated this for children younger than 5 years.

‡ We estimated a relative reduction in the prevalence of stunting instead of the absolute numbers for consistency with other indicators, because all other targets are based on prevalence.
