# Correction to *Lancet Child Adolesc Health* 2019; 3: 855–70

**DOI:** 10.1016/S2352-4642(19)30320-7

**Published:** 2019-12

*India State-Level Disease Burden Initiative Malnutrition Collaborators. The burden of child and maternal malnutrition and trends in its indicators in the states of India: the Global Burden of Disease Study 1990–2017.* Lancet Child Adolesc Health *2019; **3:** 855–70*—The first sentence of the Exclusive breastfeeding section of the Results has been corrected to read: “…with a moderate inverse correlation…”. These corrections have been made to the online version as of Sept 30, 2019, and the printed version is correct.

