# Supplementary material for: The burden of child and maternal malnutrition and trends in its indicators in the states of India: the Global Burden of Disease Study 1990–2017
Source: Lancet Child Adolesc Health. 2019 Dec;3(12):855–70. doi: 10.1016/S2352-4642(19)30273-1 (PMC6839043; doi:10.1016/S2352-4642(19)30273-1)
Supplement: Supplementary appendix [file mmc1.pdf]

THE LANCET
Child & Adolescent Health

Supplementary appendix

This appendix formed part of the original submission and has been peer reviewed. We post it as supplied by the authors.

Supplement to: India State-Level Disease Burden Initiative Malnutrition Collaborators. The burden of child and maternal malnutrition and trends in its indicators in the states of India: the Global Burden of Disease Study 1990–2017. *Lancet Child Adolesc Health* 2019; published online September 18. https://doi.org/10.1016/S2352-4642(19)30273-1.

The burden of child and maternal malnutrition and the trends in its indicators in the states of India: the Global Burden of Disease Study 1990–2017

India State-Level Disease Burden Initiative Malnutrition Collaborators

Web Appendix

Correspondence to: Prof. Lalit Dandona, lalit.dandona@phfi.org

Table of Contents

1. GBD 2017 malnutrition estimation methods 3

2. GBD 2017 India data inputs for child and maternal malnutrition exposure, covariates, and the disease burden and deaths attributable to malnutrition..25

3. Grouping of the states of India based on Socio-demographic Index, 2017 38

4. Death and DALY rates attributable to child and maternal malnutrition in under-five children in the states of India, 2017 39

5. DALY rates attributable to child and maternal malnutrition in all-age groups combined together in the states of India, 2017 39

6. Percentage contribution of DALYs and deaths attributable to child and maternal malnutrition and its components by sex in India, 2017...41

7. Projected prevalence and the NNM targets in 2022 for states of India 42

8. Projected prevalence and the WHO/UNICEF targets in 2030 for states of India 43

9. Diagnostic test for robustness of 1000 draws to estimate the 95% uncertainty intervals for low birth weight, stunting, and wasting 44

1. GBD 2017 malnutrition estimation methods

The materials presented here are adapted from the following sources:

- GBD 2017 Risk Factors Collaborators. Global, regional, and national comparative risk assessment of 84 behavioural, environmental and occupational, and metabolic risks or clusters of risks for 195 countries and territories, 1990–2017: a systematic analysis for the Global Burden of Disease Study 2017. Lancet 2018; 392: 1923-94.

- GBD 2017 Disease and Injury Incidence and Prevalence Collaborators. Global, regional, and national incidence, prevalence, and years lived with disability for 354 diseases and injuries for 195 countries and territories, 1990–2017: a systematic analysis for the Global Burden of Disease Study 2017. Lancet 2018; 392: 1789-858.

- GBD 2017 SDG Collaborators. Measuring progress from 1990 to 2017 and projecting attainment to 2030 of the health-related Sustainable Development Goals for 195 countries and territories: a systematic analysis for the Global Burden of Disease Study 2017. Lancet 2018; 392: 2091-138.

A. GBD estimation process of risk factors including child and maternal malnutrition

The analytical approach used in GBD 2017 for comparative risk assessment to estimate population attributable fractions for risk factors is shown in the following flowchart.

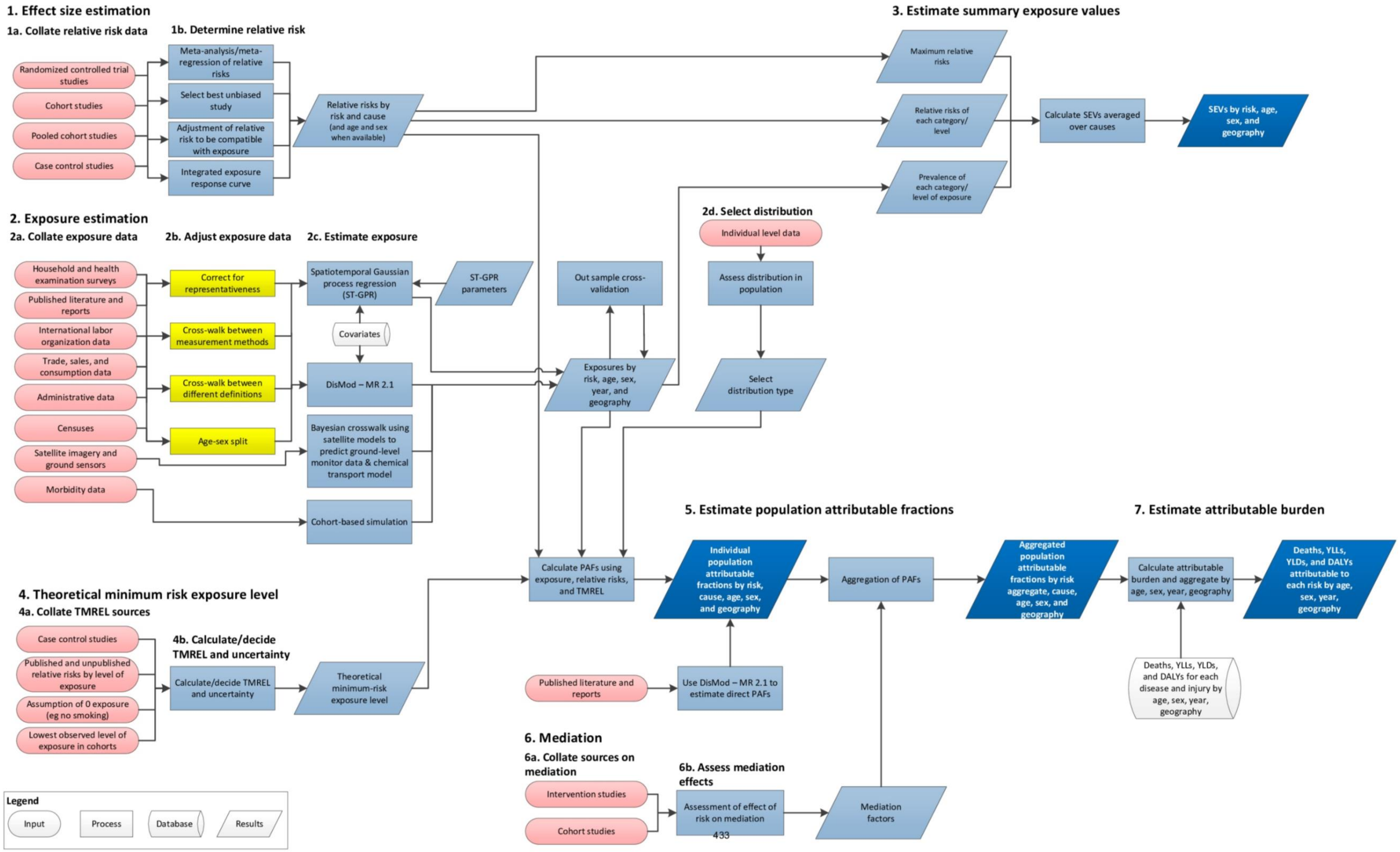

GBD is Global Burden of Disease. SEV is summary exposure value. TMREL is theoretical minimum-risk exposure level. PAF is population attributable fraction. YLL is years of life lost. YLD is years lived with disability. DALY is disability-adjusted life-years. Ovals represent data inputs, rectangular boxes represent analytical steps, cylinders represent databases, and parallelograms represent intermediate and final results.

The components of malnutrition risk factors in GBD 2017 are summarised below:

Risk factor	Level
**Child and maternal malnutrition**	**2**
Low birth weight and short gestation	3
Short gestation for birth weight	4
Low birth weight for gestation	4
Child undernutrition	3
Child stunting	4
Child wasting	4
Child underweight	4
Suboptimal breastfeeding	3
Non-exclusive breastfeeding	4
Discontinued breastfeeding	4
Iron deficiency	3
Vitamin A deficiency	3
Zinc deficiency	3

The components of child and maternal malnutrition in GBD include low birth weight and short gestation, child growth failure, suboptimal breastfeeding, and micronutrient deficiencies. Low birth weight and short gestation includes low birth weight for gestation and short gestation for birth weight, child growth failure includes child stunting, child wasting, and child underweight, suboptimal breastfeeding includes non-exclusive breastfeeding and discontinued breastfeeding, and micronutrient deficiencies include iron, vitamin A, and zinc deficiencies. We describe in detail the methods used to estimate the eight malnutrition indicators that are reported in this paper, i.e. low birth weight, child stunting, child wasting, child underweight, anaemia in children aged 0-4 years, anaemia in women aged 15-49 years, exclusive breastfeeding, overweight in children aged 2-4 years.

A.1. Low birth weight and short gestation

Low birth weight for gestation and short gestation for birth weight are separate risk factors, however the exposures and relative risks for both were estimated jointly through the low birth weight and short gestation parent risk factor. The meaning of "low birth weight" and "short gestation" in GBD have subtle definitional differences compared to other usages of "low birth weight" and "short gestation" in the literature. The term "low birth weight" has historically been used to refer to birth weight less than 2,500 grams. However, because the goal of the GBD risk factors analysis was to quantify the entirety of attributable burden due to each risk factor, the GBD definition of "low birth weight" therefore refers to all birth weight below the theoretical minimum-risk exposure level (TMREL) for birth weight. Likewise, new-borns were typically classified into gestational age categories of "extremely preterm" (<28 weeks of gestation), "very preterm" (28-<32 weeks of gestation), and "moderate to late preterm" (32-<37 weeks of gestation). "Short gestation" refers to gestational age below the gestational age TMREL. Exposures and relative risks for the GBD low birth weight and short gestation risk factors were categorised into different combinations of joint 500-gram birth weight and 2-week gestational age. The lowest risk overall 500-gram/2-week bin was the overall TMREL. The univariate TMRELs vary with gestational age and birth weight. The lowest risk gestational age varies by birth weight category and the lowest risk birth weight vary with gestational age category. The latter were used to quantify univariate attributable risk. Under this framework, all attributable burden under the joint TMREL were referred to jointly as burden of low birth weight and short gestation. All attributable burden to birth weights under the TMREL for each gestational age category were, on aggregate, "low birth weight", and all attributable burden to gestational ages under the TMREL for each birth weight category were, on aggregate, "short gestation." Each combination of 500-grams and 2-weeks was associated with a relative risk for mortality by neonatal period (early and late neonatal) and by the causes, and relative to the joint TMREL.

The steps in the estimation of low birth weight and short gestation are shown in the following flowchart:

Low birth weight and Short gestation Risk Factors

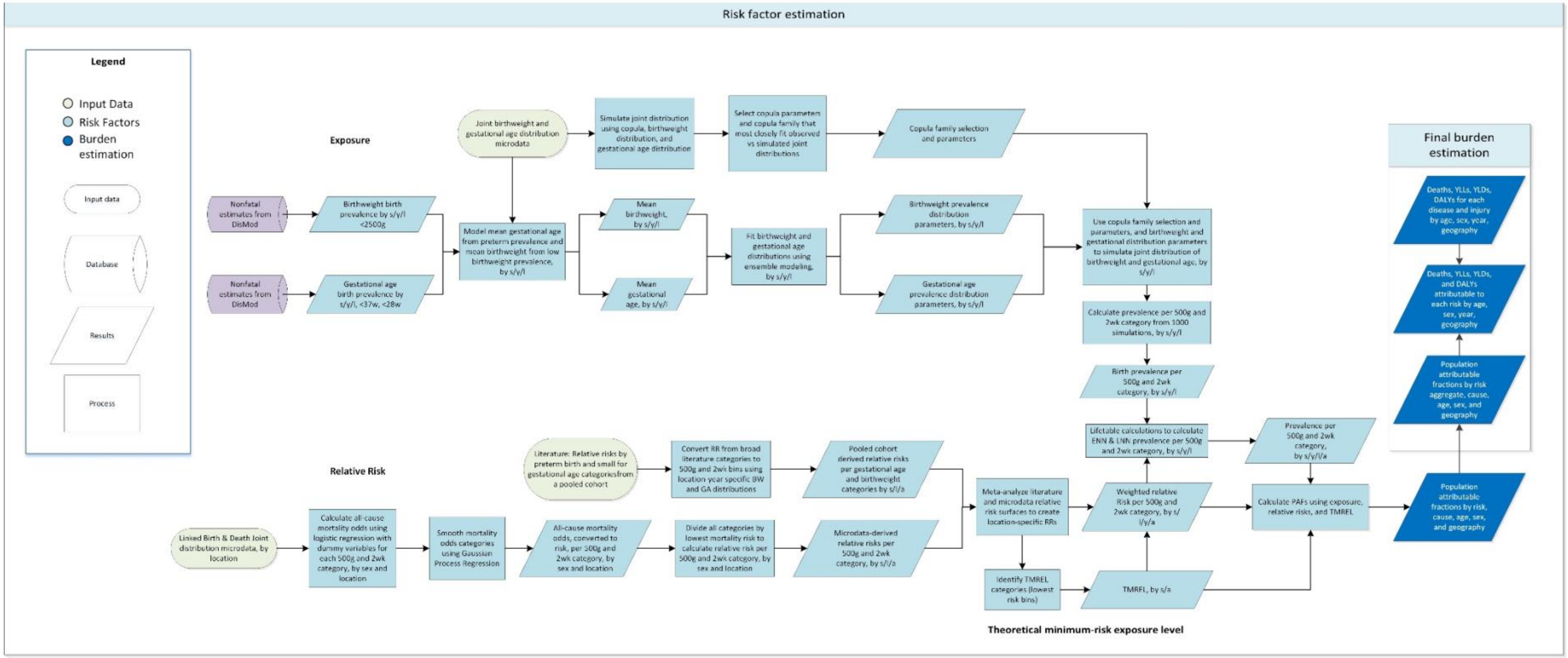

Data

To model the joint distribution of exposure of low birth weight and short gestation for each location, year, and sex estimated in GBD 2017, three types of information were used:

- Distribution of birth weight for each location, year, and sex
- Distribution of gestational age for each location, year, and sex
- Copula family and parameters, specifying correlation between gestational age and birth weight distributions

Major data inputs for India were national surveys such as National Family Health Surveys, District Level Household Surveys, and Annual Health Surveys.

Modelling

To model the joint distribution of birth weight and gestational age for every location-year-sex, ensemble model methods standard to GBD risk factors, were used to create separate distributions of birth weight and gestational age for every location-year-sex. Microdata is the most ideal data source for modelling distributions; however, microdata is not widely available for birth weight and is even more scarce for gestational age. Much more readily available, and from a wider range of locations and years, is categorical prevalence data for low birth weight (<2,500 grams), extremely preterm (<28 weeks of gestation), very preterm (28-32 weeks of gestation), moderate to late preterm (32-37 weeks of gestation), and preterm birth (<37 weeks of gestation). The full distributions at birth were modelled for gestational age and birth weight for all GBD locations, estimation years, and both sexes. The gestational age and birth weight distributions were then aggregated into the categorical estimates of <28 weeks, 28-<32 weeks, 32-<37 weeks gestation, and <2,500 grams birth weight.

Ensemble model methods standard to GBD were used to model the distribution at birth of gestational age and birth weight. Gestational age ensemble distribution models used the prevalence of <37 weeks gestation, the prevalence of <28 weeks gestation, and mean gestational age per each location-year-sex as inputs into the model. Birth weight distribution models used the prevalence of <2,500 grams birth weight and mean birth weight for each location-year-sex. Prevalence of <37 weeks gestation and of <2,500 grams birth weight was estimated for all location-year-sex using spatiotemporal regression and Gaussian Process Regression (ST-GPR) modelling process standard to GBD.

Low birth weight (<2,500 grams) data were extracted from surveys, vital registration systems, and the literature. The missing data for low birth weight in the Demographic and Health Surveys (DHS) was imputed using Amelia package in R from the following variables: urbanicity, sex, birthweight recorded on card, birth order, maternal education, paternal education, child age, child weight, child height, mother's age at birth, mother's weight, shared toilet facility, and household water treated.

Global ensemble weights for gestational age were derived by using a 3 million sample of all available microdata to select the ensemble weights. Of the exponential, gamma, inverse gamma, Weibull, log normal, and normal distributions, the three distribution families that received the highest weights were the Weibull (87%), normal (4%), and inverse gamma (4%) distributions. Global ensemble weights for birth weight were derived using a 3 million sample of all available microdata, in addition to birth weight microdata available primarily through the DHS and Multiple Indicator Cluster Surveys (MICS). Of the exponential, gamma, inverse gamma, Weibull, log normal, and normal distributions, the three distribution families that received the highest weights were the log normal (38%), normal (32%), and Weibull (20%) distributions.

Ordinary-least square was used to model mean gestational age for all location-year-sex by regressing mean gestational age on prevalence of <37 weeks gestation per location-year. All available microdata were used to fit the model. Ordinary-least square was also used to model mean birth weight by regressing prevalence of <2,500 grams birth weight per location-year. All available joint microdata, as well as additional birth weight microdata extracted primarily through DHS and MICS, were used to fit the model. As estimates of prevalence of <37 weeks gestation and prevalence of <2,500 grams birth weight was available for all location-year-sex through ST-GPR models, mean gestational age and mean birth weight were predicted for all location-year-sex.

Copula optimisation

Copula modelling is used to model joint distributions between the birth weight and gestational age marginal distributions. In order to model the joint distribution of birth weight and gestational age from separate distributions, information is needed about the correlation between the two distributions. The Spearman correlation for each country where joint microdata was available, pooling across all years of data available, ranged from 0.25-0.49 indicating that the distributions of birth weight and gestational age are not independent. The overall Spearman correlation was 0.38, pooling across all countries in the dataset. The Copula and VineCopula packages in R were used to select the optimal copula family and copula parameters to model the joint distribution, using joint microdata from the country-year. The copula family selected from the microdata was "Survival BB8", with theta parameter set to 1.75 and delta parameter set to 1.

The joint distribution of birth weight and gestational age per location-year-sex was modelled using the global copula family and parameters selected, and the location-year-sex gestational age and birth weight distributions. The joint distribution was simulated 100 times to capture uncertainty. Each simulation consisted of 100,000 simulated joint birth weight and gestational age data points. Each joint distribution was divided into 500-gram by 2-week bins to match the categorical bins of the relative risk surface. Birth prevalence was then calculated for each 500-gram by 2-week bin.

Estimating early neonatal prevalence and late neonatal prevalence from birth cohorts

Early neonatal and late neonatal prevalence was estimated using life table approaches for each 500-gram and 2-week bin. Using the all-cause early neonatal mortality rate for each location-year-sex, births per location-year-sex-bin, and the relative risks for each location-year-sex bin in the early neonatal period, the all-cause early neonatal mortality rate was calculated for each location-year-sex bin. The early neonatal mortality rate per bin was used to calculate the number of survivors at 7 days and prevalence in the early neonatal period. Using the same process, the all-cause late neonatal mortality rate for each location-year-sex was paired with the number of survivors at 7 days and late neonatal relative risks per bin to calculate late neonatal prevalence and survivors at 28 days.

Relative risks and theoretical minimum-risk exposure level

The available data for deriving relative risk was only for all-cause mortality. The relative risk of all-cause mortality across all available sources and selected outcomes was analysed based on criteria of biologic plausibility. Some causes, most notably congenital birth defects, haemoglobinopathies, malaria, and HIV/AIDS, were excluded based on the criteria that reverse causality could not be excluded. The outcomes included in calculating the attributable burden for low birth weight and short gestation were: diarrheal diseases, lower respiratory infections, upper respiratory infections, otitis media, pneumococcal meningitis, H influenza type B meningitis, meningococcal meningitis, other meningitis, encephalitis, neonatal preterm birth complications, neonatal encephalopathy due to birth asphyxia and trauma, neonatal sepsis and other neonatal infections, hemolytic disease and other neonatal jaundice, other neonatal disorders, and sudden infant death syndrome.

GBD has so far attributed disease burden to low birth weight and short gestation only for the neonatal period. Further analytical work is planned to able to use the available evidence to estimate disease burden that may be attributable in the post-neonatal period as well.

Epidemiological evidence for relative risk of various disease outcomes attributable to low birth weight for gestation was obtained from the following study:

Katz J, Lee AC, Kozuki N, et al. Mortality risk in preterm and small-for-gestational-age infants in low-income and middle-income countries: a pooled country analysis. Lancet 2013; 382: 417–25.

For each location, data were pooled across years, and the risk of all-cause mortality at the early neonatal period and late neonatal period at joint birth weight and gestational age combinations was calculated. To calculate relative risk at each 500-gram and 2-week combination, logistic regression was first used to calculate mortality odds for each joint 2-week gestational age and 500-gram birth weight category. Mortality odds were smoothed with Gaussian Process Regression, with the independent distributions of mortality odds by birth weight and mortality odds by gestational age serving as priors in the regression.

A pooled country analysis[1] of mortality risk in the early neonatal period and late neonatal period by short gestational age category in developing countries in Asia, the Americas, and Sub-Saharan Africa were also converted into 500-gram and 2-week bin mortality odds surfaces. Location-specific relative risk surfaces, derived from location-specific estimates of with-condition mortality of preterm birth, were converted into 500-gram and 2-week bin mortality odds. The US, Japan, Singapore, pooled country analysis, and location-specific with-condition mortality surfaces were meta-analysed, resulting in a meta-analysed mortality odds surface for each location. The meta-analysed mortality odds surface for each location was smoothed using Gaussian Process Regression and then converted into mortality risk. To calculate mortality relative risks, the risk of each joint 2-week gestational age and 500-gram birth weight category were divided by the risk of mortality in the joint gestational age and birth weight category with the lowest mortality risk.

For each of the country-derived relative risk surfaces, the 500-gram and 2-week gestational age joint bin with the lowest risk was identified. This bin differed within each country dataset. To identify the universal 500-gram and 2-week gestational age category that would serve as the universal TMREL for our analysis, we chose the bins that was identified to be the TMREL in each country dataset to contribute to the universal TMREL. Therefore, the joint categories that served as our universal TMREL for the low birth weight and short gestation risk factor were "38-40 weeks of gestation and 3,500-4,000 grams", "38-40 weeks of gestation and 4,000-4,500 grams", and "40-42 weeks of gestation and 4,000-4,500 grams". As the joint TMREL, all three categories were assigned to a relative risk equal to 1.

Population attributable fraction calculations

The total population attributable fraction (PAF) for the low birth weight and short gestation joint risk factor was calculated by summing the PAF calculated from each 500-gram and 2-week category, with the lowest risk category among all the 500-gram and 2-week categories serving as the TMREL. The equation for calculating PAF for each 500-gram and 2-week category is:

$$PAF_{joasgt} = \frac{\sum_{x=1}^{u} RR_{joast}(x)P_{jasgt}(x) - RR_{joasg}(TMRE_{jas})}{\sum_{x=1}^{u} RR_{joas}(x)P_{jasgt}(x)}$$

To calculate the overall PAF for the short gestation for birth weight risk factor, PAF was once again calculated for each joint 500-gram and 2-week category. Unlike the joint PAF calculation, which used only one TMREL for all 500-gram and 2-week categories, the joint 500-gram and 2-week category with the lowest risk for each 500-gram birth weight grouping served as the TMREL for that 500-gram birth weight grouping. For example, the [3,000, 3,500] gram birth weight grouping contains five joint categories: [34, 36] weeks and [3,000, 3,500] grams; [36, 37] weeks and [3,000, 3,500] grams; [37, 38] weeks and [3,000, 3,500] grams; [38, 40] weeks and [3,000, 3,500] grams; and [40, 42] weeks and [3,000, 3,500] grams. The [40, 42] weeks and [3,000, 3,500] grams joint category has the lowest risk, and so it serves as the TMREL for the [3,000, 3,500] gram birth weight grouping. In the relative risk surface figures, a birth weight grouping was one “column” of the birth weight and gestational age matrix.

The overall PAF for the short gestation for birth weight risk factor was then calculated for all the joint 500-gram and 2-week categories using the formula below:

$$PAF_{1..i} = 1 - \prod_{i=1}^{n}(1 - PAF_i)$$

The same methodology was applied to calculate the total PAF for the low birth weight for gestation risk factor, using 2-week gestational age categories (each “row” of the matrix) instead of 500-gram birth weight categories. For example, the [24, 26] weeks gestational age grouping contains three joint categories: [0, 500] grams and [24, 26] weeks; [500, 1,000] grams and [24, 26] weeks; and [1,000, 1,500] grams and [24, 26] weeks. The [1,000, 1,500] grams and [24, 26] weeks joint category has the lowest risk, and so it serves as the TMREL for the [24, 26] weeks gestational age grouping.

After the short gestation for birth weight PAF and low birth weight for gestational age PAF were calculated, they were then scaled so that the sum of the short gestation for birth weight PAF and low birth weight for gestation PAF equal the low birth weight and short gestation parent PAF calculated for each location-year-sex-age group.

A.2. Child growth failure

Child growth failure was estimated using three indicators stunting, wasting, and underweight all of which are based on categorical definitions using the World Health Organisation (WHO) 2006 growth standards for children 0-59 months. Definitions were based on z-scores from the growth standards, which were derived from an international reference population. Mild, moderate, and severe categorical prevalences were estimated for each of the three indicators.

TMREL for stunting, wasting, and underweight was assigned to be greater than or equal to -1 standard deviation (SD) of the WHO 2006 standard height-for-age, weight-for-height, and weight-for-age curves, respectively.

The steps in the estimation of child growth failure are shown in the following flowchart:

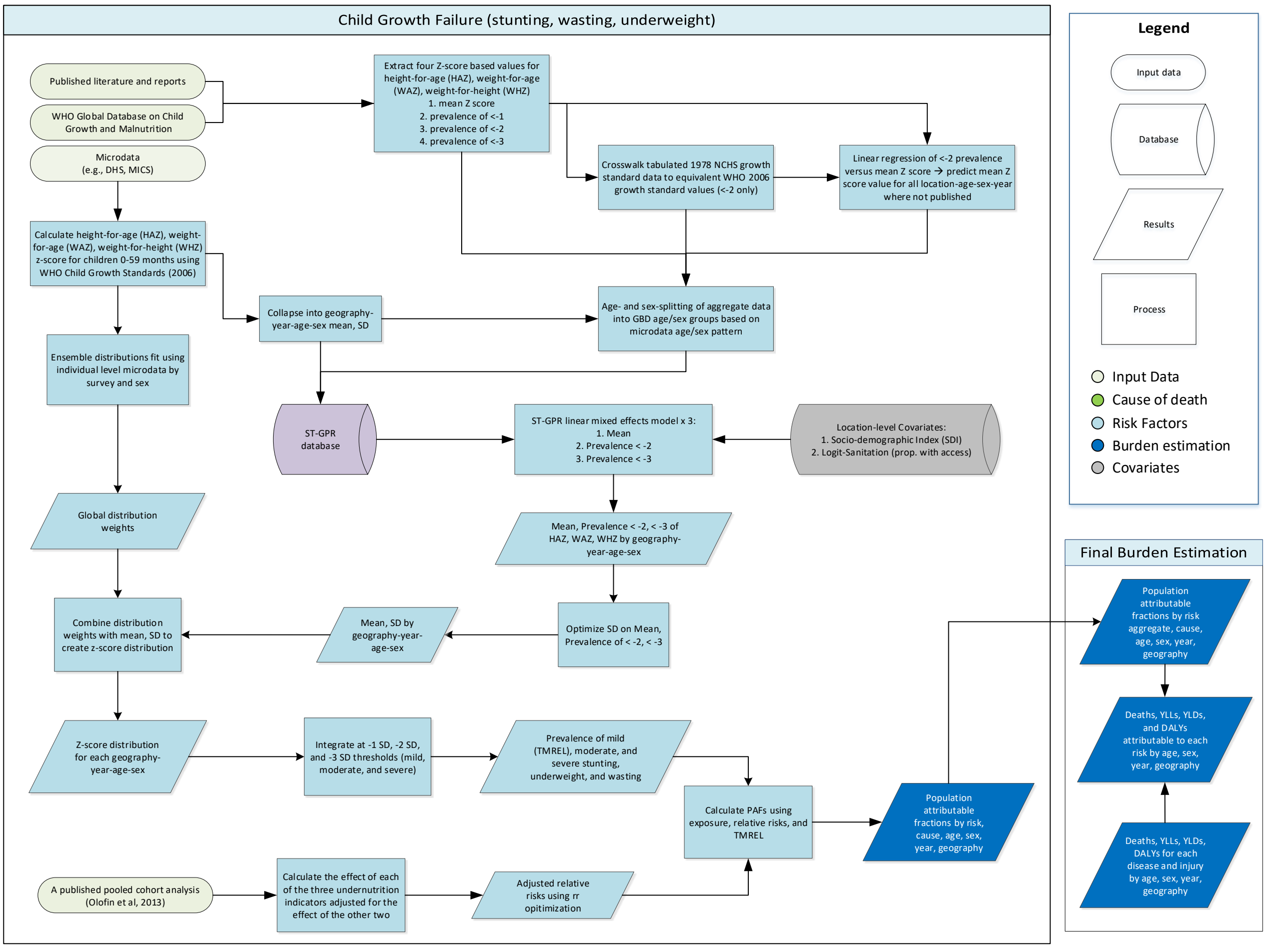

Data

The three main inputs for the GBD child growth failure models were: microdata from population-based surveys including anthropometric surveys, tabulated data from reports and published literature, and the WHO Global Database on Child Growth and Malnutrition.[2] Population surveys include a variety of multi-country and country-specific survey series such as DHS, MICS, Living Standards Measurement Surveys, and the China Health and Nutrition Survey, as well as other one-time country-specific surveys. These microdata contain information about each individual child's age (from which age in weeks and age in months are calculated), as well as height and/or weight. From that information, a height-for-age z-score (HAZ), weight-for-height z-score (WHZ), and weight-for-age z-score (WAZ) were calculated using the WHO 2006 Child Growth Standards and the LMS method.[3]

All available data from the WHO Global Database on Child Growth and Malnutrition were extracted – majority of which was from published studies. Four metrics that were sought from all sources with tabulated data were: mean z-score, prevalence <-1 z-score (mild), prevalence <-2 z-score (moderate), and prevalence <-3 z-score (severe). All

data for each metric was extracted for each of stunting (height-for-age z-score; HAZ), wasting (weight-for-height z-score; WHZ), and underweight (weight-for-age z-score; WAZ).

To maximise internal-consistency and comprehensiveness of the modelling dataset, three data transformations were performed. Firstly, any data that were reported using the National Center for Health Statistics (NCHS) 1978 growth standards were crosswalked to corresponding values on the WHO 2006 Growth Standards curves based on a study that evaluated growth standard concordance.[4] Crosswalks from 1978 to 2006 growth standards were performed only on <-2 z-score (i.e. moderate) prevalence data as that was where the concordance was most consistent. Secondly, for any study that lacked a measure of mean z-score for any of stunting, wasting, or underweight, a mean value was predicted for that study based on an ordinary-least squares regression of mean z-score versus <-2 z-score prevalence for that metric from all sources where both were available. Thirdly, any data that were presented as both sexes combined for 0-59 months, the age- and sex-pattern were used from all data sources that included that detail to split these data into corresponding age-and sex-specific data.

The major data sources from India were national surveys including National Family Health Surveys, District Level Household Surveys, Annual Health Survey, National Nutrition Monitoring Bureau Diet and Nutritional Surveys, and Rapid Survey on Children.

Modelling

The following three-step modelling process was used to estimate stunting, wasting, and underweight.

First, all microdata was fit using an ensemble modelling process. A series of 12 individual distributions (normal, log normal, log logistic, exponential, gamma, mirror gamma, inverse gamma, gumbel, mirror gumbel, Weibull, inverse Weibull, and beta) were fit to the entire set of microdata (approximately 2.5 million individual z-scores) at the individual survey level. A weighting algorithm combined each distribution to find the optimal combination of these distributions for each survey, minimising the absolute prediction error across the entire distribution. Ensemble weights for each survey were then averaged across all surveys to produce a single set of global weights of the ensemble distributions. Weights were different for each sex, but invariant across geography, time, and age group. All component distributions that were used to derive weights were parameterised using “method of moments,” meaning that each corresponding probability density function could be described as a function of the mean and variance of the quantity of interest.

Second, models were developed for mean z-scores and prevalence of moderate and severe growth failure. Individual level microdata were collapsed to calculate three metrics: mean z-score, moderate prevalence, and severe prevalence. These data were combined with that derived from literature, GHDx review, and the WHO Global Database on Child Growth and Malnutrition. For those sources where moderate prevalence was reported without a corresponding mean, a predicted mean was calculated using an ordinary-least square regression from those sources where both metrics were present. Each of the three metrics were then modelled using ST-GPR, generating estimates for each location, year, age group, and sex.

Third, estimates of mean and prevalence (moderate and severe) were combined with ensemble weights in an optimisation framework in order to derive the variance that would best correspond to the predicted mean and prevalence. This variance was then paired with the mean, and using the method of moments equation for each of the component distributions of the ensemble, probability density function of the distribution of z-scores were calculated for each location, year, age group, and sex. Probability density functions were integrated to determine the prevalence between -1 and -2 z-scores (mild), between -2 and -3 z-scores (moderate), and below -3 z-scores (severe). These were categorical exposures used for subsequent attributable risk analysis.

All models were run with the complete dataset. Data plausibility inspection began with examination of time-trends in stunting. If a given datum was judged to have led to a change in the prevalence of moderate stunting in 1-4 year olds of 50% or greater in 5 years or fewer, and was inconsistent with data prior to and after that year (a change considered implausible), the offending datum were outliered and the model was re-run. The results of moderate stunting, wasting, and underweight were further visually-inspected in parallel to look for location-year-age-sex where the results were not internally-consistent (e.g. underweight rapidly increasing, and stunting and wasting decreasing). This inspection revealed very few inconsistent data.

Relative risks were derived from a pooled cohort analysis.[5] The final list of outcomes paired with child growth failure risks included lower respiratory infections, diarrhoea, measles, and protein energy malnutrition as shown in the below table.

Epidemiological evidence of association of stunting, wasting, and underweight, with diarrheal diseases, lower respiratory infections and measles was obtained from the following study:

Olofin I, McDonald CM, Ezzati M, et al. Associations of suboptimal growth with all-cause and cause-specific mortality in children under five years: a pooled analysis of ten prospective studies. *PLoS ONE* 2013; 8: e64636.

There is a high degree of correlation between stunting, wasting, and underweight. Failing to account for their covariance and assuming independence would significantly overestimate the total burden. A method developed by Olofin and colleagues was used to adjust observed relative risks by simulating the joint distribution of the three indicators using the distribution of each indicator and covariance between indicators in the countries included in the meta-analysis (extracted from DHS micro-data).[6] Based on the analysis done by McDonald and colleagues, it was assumed that there is an interaction between the three indicators, and the interaction terms were extracted from the corresponding analysis. The adjusted relative risks were calculated by minimising the error between observed crude relative risks (from meta-analysis) and expected crude relative risks derived from adjusted relative risks.

The relative risks were adjusted using an optimisation algorithm that takes into account covariance between the three child growth failure indicators. Of historical note, upper respiratory infections and otitis media were previously included as outcomes, based on the “analogy” causal criterion, assuming there is similar pathway as lower respiratory infections outcome. However, closer review did not find sufficient evidence to support their inclusion and they were excluded. 100% of protein energy malnutrition were attributed to childhood wasting and underweight but not stunting. The final list of relative risk outcomes paired with child growth failure risks included lower respiratory infections, diarrhoea, measles, and protein energy malnutrition as shown in the below table.

Adjusted RRs for each risk-outcome pair for child growth failure

Outcome	Stunting	Wasting	Underweight
Diarrhea	<-1: 1.111 (1.023-1.273)<br><-2: 1.222 (1.067-1.5)<br><-3: 1.851 (1.28-2.699)	<-1: 6.601 (2.158-11.243)<br><-2: 23.261 (9.02-35.845)<br><-3: 105.759 (42.198-157.813)	<-1: 1.088 (1.046-1.134)<br><-2: 1.23 (1.163-1.314)<br><-3: 2.332 (2.076-2.802)
Lower respiratory infections	<-1: 1.125 (0.998-1.655)<br><-2: 1.318 (1.014-2.165)<br><-3: 2.355 (1.15-5.114)	<-1: 5.941 (1.972-11.992)<br><-2: 20.455 (70.84-37.929)<br><-3: 47.67 (15.923-94.874)	<-1: 1.145 (1.044-1.364)<br><-2: 1.365 (1.215-1.755)<br><-3: 2.593 (1.908-4.39)
Measles	<-1: 1.103 (0.861-1.719)<br><-2: 1.540 (1.029-3.222)<br><-3: 2.487 (1.129-6.528)	<-1: 1.833 (0.569-8.965)<br><-2: 8.477 (1.33-42.777)<br><-3: 37.936 (5.088-199.126)	<-1: 0.995 (0.5-1.726)<br><-2: 2.458 (1.26-5.118)<br><-3: 5.668 (1.767-12.414)
Protein-energy malnutrition	0% PAF	100% PAF	100% PAF

<-1, <-2, and <-3 refer to categories of stunting, wasting, and underweight that are less than -1, -2, and -3 standard deviations of the median in the WHO 2006 standard curve.

A.3. Anaemia impairment

The prevalence of anaemia was defined using the following WHO thresholds for haemoglobin.

	Severity of anaemia		
	**Mild**	**Moderate**	**Severe**
**Age < 1 month**			
Males	130 - 149 g/L	90 - 129 g/L	< 90 g/L
Females	130 - 149 g/L	90 - 129 g/L	< 90 g/L
**Age 1 month-4 years**			
Males	100 - 109 g/L	70 - 99 g/L	< 70 g/L
Females	100 - 109 g/L	70 - 99 g/L	< 70 g/L
**Age 5–14 years**			
Males	110 - 114 g/L	70 - 99 g/L	< 70 g/L
Females	110 - 114 g/L	70 - 99 g/L	< 70 g/L
**Age 15+ years**			
Males	110 – 129 g/L	80 - 109 g/L	< 80 g/L
Females, non-pregnant	110 - 119 g/L	80 - 109 g/L	< 80 g/L
Females, pregnant	100 – 109 g/L	70 - 99 g/L	< 70 g/L

The steps in the estimation of non-fatal anaemia burden or morbidity are shown in the following flowchart:

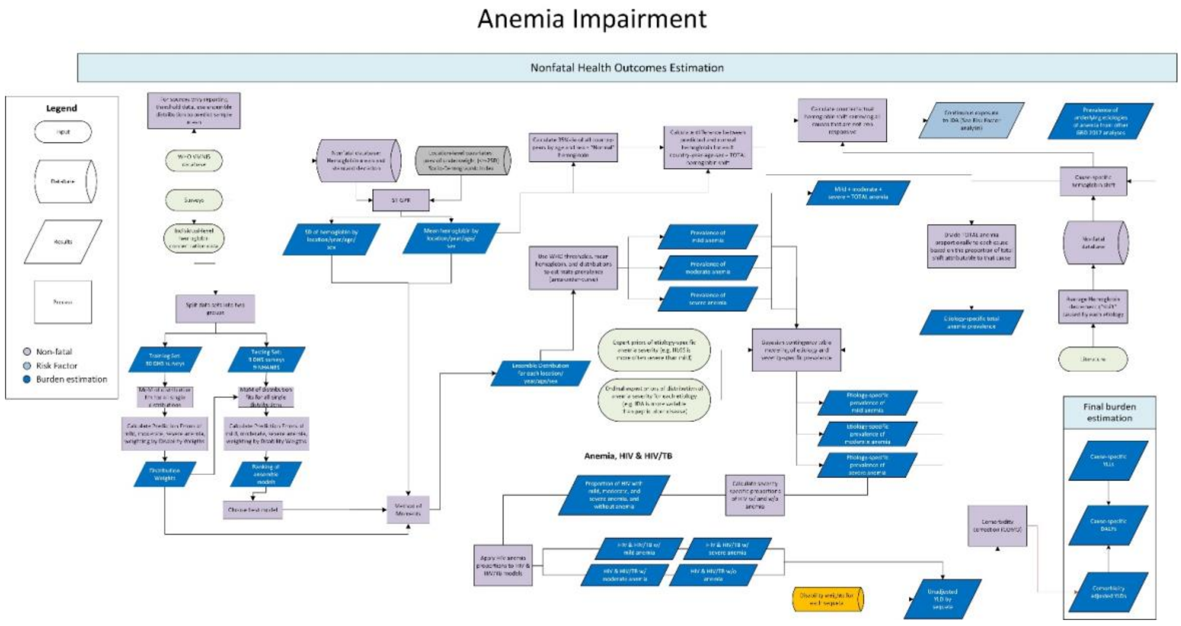

Data

The envelope approach to anaemia impairment utilises data from a variety of sources. Population based surveys of haemoglobin concentration were the primary input to the analytic dataset. Examples include the DHS and MICS series, along with other national and sub-national surveys that completed haemoglobin testing. We supplemented with pertinent sources downloaded from the WHO Vitamin and Mineral Nutrition Information System (VMNIS) available at http://www.who.int/vmnis/database/anaemia/countries/en/. Most used a HemoCue test, adjusted for altitude, and excluded those with terminal or acute medical conditions. Inclusion, exclusion, and diagnostic criteria for other studies were similar and can be found in each study. Any data that were presented as both sexes combined or an age range encompassing more than one GBD age group were split by age and/or sex prior to modelling.

The major data sources from India were national surveys including National Family Health Surveys, District Level Household Surveys, Annual Health Surveys, Medical Certification of Cause of Death data, Sample Registration System Verbal Autopsy data, Micronutrient Deficiency Survey, Study on Causes of Death by Verbal Autopsy, and WHO global database on anaemia, nutrition landscape information system.

Modelling of anaemia impairment envelope

1. Estimation of population mean and standard deviation of haemoglobin

   Two Dismod-MR models were run – one for mean haemoglobin and one for SD of haemoglobin. In both models, we included fixed effects on underweight (proportion of children under-five <2SD weight-for-age), and Socio-demographic index (SDI). Mean haemoglobin was used as a fixed effect in the SD model. The models were run with the following parameters:

	**Haemoglobin**	**Haemoglobin SD**
predict_re	0	0
custom_hyperparameters	1	0
density_cutoffs	5, 10, 15	
st_lambdaa	2, 2, 1.5, 1	
st_omega	2, 2 ,2, 2	
st_zeta	0.7, 0.8, 0.9, 0.99	
gpr_scale	15, 15, 15, 10	
amp_factor	1	1
level_4_to_3_agg	6, 130, 163, 179, 180	6, 130, 163, 179, 180
level_5_to_4_agg	6, 163, 180	6, 163, 180
add_nsv	1	1

2. Estimation of prevalence of anaemia by severity

The full distribution of haemoglobin were modelled for each population (location/year/age/sex), from which we applied the WHO thresholds to calculate prevalence of each severity of anaemia. In GBD 2015, a Weibull distribution was fit using shape and scale parameters estimated from mean haemoglobin. Similar to GBD 2016, multiple two-parameter distributions were combined to create a more precise and unbiased ensemble distribution.

First, a training and testing set of individual-level haemoglobin measurements were created. The training set consisted of 90 DHS surveys, providing 290 group-specific samples of microdata from children under-five years, males 15-45 years, pregnant females 15-45 years, and non-pregnant females 15-45 years (not all groups were sampled in each DHS). A set of two-parameter distributions (gamma, mirror gamma, Weibull, mirror lognormal, and mirror gumbel) were fit to the sample's haemoglobin mean and variance. These distributions were combined using weights optimised by a loss function of severity-specific prediction error weighted by the ratio of the severity's disability weight (DW) to mild anaemia DW. Weights were constrained to be positive and sum to 1, so that the resultant ensemble distribution is a proper probability density function. All permutations of the 5 distributions were tested (ie, we optimised weights for both a mix of all 5 distributions as well as a gamma-Weibull two-way combination).

The loss function is

$$\sum_{i=1}^{n_i}\sum_{j=1}^{n_j}\sum_{k=1}^{n_k} r_j\,|p_{ijk} - \hat{p}_{ijk}|$$

Where

$$\hat{p}_{ijk} = \sum_{z=1}^{n_z} w_z \int_{t1_{jk}}^{t2_{jk}} PDF_{ijz}$$

n_i is a list of surveys (in either the training or testing set); nj is the list of groups: children under-five years, males 15-45 years, pregnant females 15-45 years, non-pregnant females 15-45 years, males >45 years, and females >45 years; n_k is the list of severities (mild, moderate, severe); n_z is the list of distributions (gamma, mirror gamma, Weibull, mirror lognormal, and mirror gumbel); r is the ratio of the severity j DW to that of mild anaemia; r_k = 13 for moderate and r_k = 40 for severe; PDF is a probability density function fit to the sample mean and variance; t1 and t2 are the lower and upper bounds to the WHO anaemia definition for the group; w is the set of distribution weights (each constrained to be positive) such that

$$\sum_{z=1}^{n_z} w_z = 1 \text{ and all } w_z > 0$$

Therefore, $\sum_{z=1}^{n_z} w_z * PDF_z$ describes the ensemble probability density function that can be integrated to calculate prevalence for any severity.

The testing set consisted of 9 NHANES and 9 DHS surveys not included in the training data. Inclusion of NHANES as half the testing set ensured out of sample predictive validity by challenging the global weights, as it provided the ensemble distribution with high-income data (DHS is from LMIC countries) and data from adults >45 years (DHS did not take blood tests from the elderly). We selected the combination of distributions (including all individual component distributions) that minimised the loss function.

With a set of component distributions and global weights, the distribution of haemoglobin in each location/year/age/sex were then modelled by fitting each component distribution using modelled mean and SD, then weighting to create the ensemble distribution $\sum_{z=1}^{n_z} w_z * PDF_z$. The area under the $\sum_{z=1}^{n_z} w_z * PDF_z$ curve for each group-specific WHO threshold to calculate prevalence of anaemia by severity was integrated.

Because anaemia thresholds depend on pregnancy, the distribution of pregnant and non-pregnant females was modelled separately. The method for fitting the ensemble distribution to pregnant women was identical to that of non-pregnant, but used the mean and variance from the two DisMod models adjusted by the estimated beta on

the pregnancy status fixed effect. The prevalence of anaemia in pregnant and non-pregnant women were weighted by the pregnancy rate and combined to estimate population prevalence of anaemia. The pregnancy rate for each age was estimated as

$$pregnancy = (ASFR + SB) * 46/52$$

Where ASFR is the location- and age-specific fertility rate, and SB is the location-specific stillbirth rate.

Due to instability in the prevalence estimates in early life, the prevalence estimates for the post-neonatal age group were copied over the early and late neonatal estimates for each location, year, and sex.

Causal attribution

The cause-specific attribution on the anaemia envelope were performed using information on cause-specific prevalence and haemoglobin shift, and a number of causes and updates to haemoglobin shifts for inputs to causal attribution. Total "haemoglobin shift" was determined as the difference between the normal and predicted mean haemoglobin levels for each population group. The normal haemoglobin level was denoted as the global 95th percentile of the distribution of mean-haemoglobin within each age group, sex, and year. A total shift for each country in the corresponding age group, sex, and year was determined by finding the difference between the global "normal" and the country-specific predicted mean haemoglobin. The model of attribution followed that, because the shift was a disease state experienced by 100% of the population, the sum of cause-specific haemoglobin shifts times the prevalence of each contributing cause should add up to the total. The shift times prevalence estimates were summed from all causes, compared to the total predicted haemoglobin shift, and proportionally assigned. The residual envelope was distributed among seven remaining causes.

Of note, our iron-deficiency anaemia estimates included acute and chronic hemorrhagic states for which supplementation may be helpful, but poor nutritional intake was not the only underlying problem. A few causes in this category – hookworm, schistosomiasis, upper gastrointestinal bleeding, and gynecologic diseases – were considered separately from iron-deficiency anaemia because there was enough data from GBD prevalence estimation processes to do so. Distribution of anaemia burden to iron-deficiency anaemia only after assignment to "known" causes avoided double counting of these cases. Most other causes of anaemia not specifically considered were included in the "other" categories.

For all causes with population-specific prevalence estimates, a condition was enforced where the sum of mild, moderate, and severe anaemia would not exceed the total prevalence within each population. Additionally, because inherent in the method of determining "normal" haemoglobin is the fact that 5% of population groups will have zero, or negative total shift, we assigned a minimum of 10% of all anaemia to residual causes based on review of findings from National Health and Nutrition Examination Survey in the United states.[8,9]

The Bayesian contingency table modelling methods were used to disaggregate marginal estimates of anaemia severity and aetiology into a complete set of prevalence estimates for aetiology/severity pairs. Marginal estimates of column sums (total anaemia prevalence by severity [mild, moderate, severe]) and row sums (total aetiology prevalence for each cause) were paired with priors on the etiology-specific haemoglobin shifts (the same as were used for overall etiologic attribution) and rank order of variation of severity (e.g. malaria-induced anaemia severity is highly variable while that due to homozygous sickle cell disease is so less). Nonlinear optimisation methods were then used to populate a complete matrix of aetiology-severity estimates from the marginal estimates and distribution priors. The maximum a posteriori (MAP) point estimate were found for 5 samples from estimated posterior distributions independently for each population group, then scaled the results to ensure row sums were non-zero and column sums matched the original draws. The mean of the scaled posteriors was taken for each population group. To estimate uncertainty for each scaled posterior mean, first the ratio of each draw to the mean of all draws for the anaemia envelope were calculated. For non-residual causes, the ratio of each draws were also calculated to the mean of all draws. The scaled posterior mean was then multiplied by these ratios.

The anaemia causal attribution process produced estimates for mild, moderate, and severe anaemia due to HIV. Using these estimates, we calculated proportions of HIV with mild, moderate, severe, and no anaemia for each demographic group. GBD produces estimates for seven HIV sub-causes: early HIV, symptomatic HIV, AIDS with antiretroviral treatment, AIDS without antiretroviral treatment, drug-sensitive HIV/AIDS – Tuberculosis,

multidrug-resistant HIV/AIDS – Tuberculosis, and extensively drug-resistant HIV/AIDS –Tuberculosis. It was assumed that the anaemia severity proportions were equivalent across the seven sub-causes, and the anaemia severity levels were estimated for each by multiplying the HIV sub-causes by the anaemia proportions.

For GBD 2017, several updates were made to the anaemia cause list. In 2017, "other gynaecological disorders" were removed from the cause list, and instead "menstrual disorders" were added, which is a directly modelled non-residual cause. Cirrhosis, Crohn's disease, ulcerative colitis, and Vitamin A deficiency were also added to the list. Because reviewed studies provided insufficient evidence of vitamin A deficiency causing any haemoglobin shift in adults,[10] the vitamin A deficiency input model was age-restricted, such that the haemoglobin shift was only applicable to age groups under 15 years.

Priors: Largest

**Anaemia subtype**	**Expected largest***
P. falciparum parasitaemia without clinical malaria	2
P. vivax parasitaemia without clinical malaria	2
Clinical malaria	2
Schistosomiasis	0
Hookworm disease	0
Other neglected tropical diseases	0
Maternal haemorrhage	1
Iron deficiency	0
Vitamin A deficiency	0
Other infectious diseases	0
Peptic ulcer disease	2
Gastritis	2
Stage III chronic kidney disease due to diabetes mellitus type 1	1
Stage IV chronic kidney disease due to diabetes mellitus type 1	1
Stage V chronic kidney disease due to diabetes mellitus type 1	2
Stage III chronic kidney disease due to diabetes mellitus type 2	1
Stage IV chronic kidney disease due to diabetes mellitus type 2	1
Stage V chronic kidney disease due to diabetes mellitus type 2	2
Stage III chronic kidney disease due to hypertension	1
Stage IV chronic kidney disease due to hypertension	1
Stage V chronic kidney disease due to hypertension	2
Stage III chronic kidney disease due to glomerulonephritis	1
Stage IV chronic kidney disease due to glomerulonephritis	1
Stage V chronic kidney disease due to glomerulonephritis	2
Stage III chronic kidney disease due to other and unspecified causes	1
Stage IV chronic kidney disease due to other and unspecified causes	1
Stage V chronic kidney disease due to other and unspecified causes	2
Uterine fibroids	0
Menstrual disorders	0
Other haemoglobinopathies and haemolytic anemias	1
Other endocrine, nutrition, blood, and immune disorders	0
G6PD deficiency	1
Hemizygous G6PD deficiency	1
Beta-thalassaemia major	2
Beta-thalassaemia trait	1
Haemoglobin E trait	0
Haemoglobin E/beta-thalassaemia	2
Haemoglobin H disease	2
Homozygous sickle cell and severe sickle cell/beta-thalassaemia parent	2
Hemoglobin SC disease	2
Mild sickle cell/beta-thalassaemia	2
Sickle cell trait	0
HIV	0
Cirrhosis and other chronic liver diseases, decompensated	1
Ulcerative colitis	0
Crohn's disease	0

*0 = mild anaemia; 1 = moderate anaemia; 2 = severe anaemia

Priors: Severity

Anaemia subtype	Expected variation
P. falciparum parasitaemia without clinical malaria	1
P. vivax parasitaemia without clinical malaria	2
Clinical malaria	3
Gastritis	4
Peptic ulcer disease	5
Crohn’s disease	6
Ulcerative colitis	7
Maternal haemorrhage	8
Other endocrine, blood, and immune disorders	9
Menstrual disorders	10
Uterine fibroids	11
Iron deficiency	12
Vitamin A deficiency	13
Other infectious diseases	14
Other neglected tropical diseases	15
HIV	16
Stage III chronic kidney disease due to other and unspecified causes	17
Stage III chronic kidney disease due to hypertension	18
Stage III chronic kidney disease due to diabetes mellitus type 1	19
Stage III chronic kidney disease due to diabetes mellitus type 2	20
Stage III chronic kidney disease due to glomerulonephritis	21
Stage IV chronic kidney disease due to other and unspecified causes	22
Stage IV chronic kidney disease due to hypertension	23
Stage IV chronic kidney disease due to diabetes mellitus type 1	24
Stage IV chronic kidney disease due to diabetes mellitus type 2	25
Stage IV chronic kidney disease due to glomerulonephritis	26
Cirrhosis and other chronic liver diseases, decompensated	27
Stage V chronic kidney disease due to other and unspecified causes	28
Stage V chronic kidney disease due to hypertension	29
Stage V chronic kidney disease due to diabetes mellitus type 1	30
Stage V chronic kidney disease due to diabetes mellitus type 2	31
Stage V chronic kidney disease due to glomerulonephritis	32
Haemoglobin E trait	33
Sickle cell trait	34
Beta-thalassaemia trait	35
G6PD deficiency	36
Hemizygous G6PD deficiency	37
Haemoglobin SC disease	38
Schistosomiasis	39
Haemoglobin H disease	40
Other haemoglobinopathies and haemolytic anaemias	41
Mild sickle cell/beta-thalassaemia	42
Hookworm disease	43
Haemoglobin E/beta-thalassaemia	44
Homozygous sickle cell and severe sickle cell/beta-thalassaemia	45
Beta-thalassaemia major	46

Causes for which allocation of residual anaemia envelope was based on fixed proportion redistribution methods*:

1. Iron-deficiency anaemia
2. Other infectious diseases
3. Other neglected tropical diseases
4. Other endocrine, nutrition, blood and immune disorders
5. Other haemoglobinopathies and hemolytic anaemias

*A minimum of 10% of all anaemia was assigned to residual categories based on the analysis of NHANES-III data from the United States.

A.4. Suboptimal breastfeeding

Exposure to suboptimal breastfeeding is composed of two distinct categories: nonexclusive breastfeeding and discontinued breastfeeding.

Non-exclusive breastfeeding is defined as the proportion of children under 6 months of age who are not exclusively breastfed. Those not exclusively breastfed are then divided into 3 categories – predominant, partial, and no breastfeeding. Exclusive breastfeeding is defined as the proportion of children who receive no other food or drink except breast milk (allowing for ORS, drops, or syrups containing vitamins, minerals, or medicines). Predominant breastfeeding is the proportion of children whose predominant source of nourishment is breast milk, but also receive other liquids. Partial breastfeeding refers to those infants who receive breastmilk as well as food and liquids, including non-human milk and formula. No breastfeeding refers to infants who do not receive breast milk as a source of nourishment. Discontinued breastfeeding is defined as the proportion of children between 6-23 months who received no breast milk.

For non-exclusive breastfeeding, those children that received no source of nourishment other than breast milk (“exclusively breastfed”) were considered to be at the lowest risk of any of the disease outcomes. For discontinued breastfeeding, the children aged 6-23 months that received any breast milk as a source of nourishment were assumed to be at the lowest risk of disease outcome.

The steps in the estimation of suboptimal breastfeeding are shown in the following flowchart:

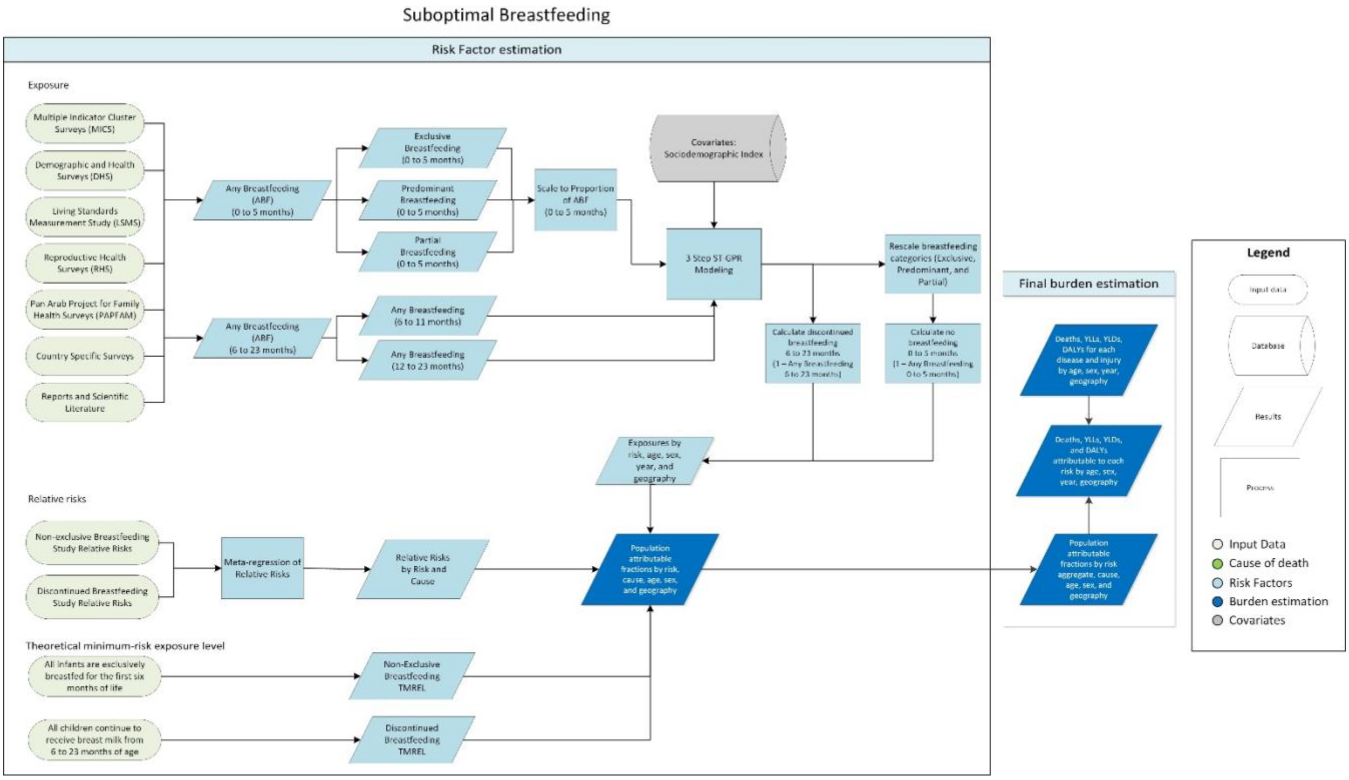

Data

There were substantial exposure data updates made for suboptimal breastfeeding for GBD 2017, including extracting identified surveys not included in previous rounds and re-extracting all surveys for new GBD 2017 subnational locations. The data used in the analysis consists mostly of processed individual-level microdata from surveys; in the cases where microdata was unavailable, the reported tabulated data were used from survey reports and scientific literature. Data used to categorise type of non-exclusive breastfeeding (predominant, partial, and none) come from surveys with 24-hour dietary logs based on maternal recall.

The major data sources for India were National Family Health Surveys and MICS.

Modelling

Using the processed microdata and tabulated data from reports, a complete time series was generated from 1980–2017 for the prevalence of breastfeeding patterns for children 0-5 months and 6-23 months using a three-step ST-GPR modelling process.

First, a robust linear regression was estimated using each geography's socio-demographic index as a covariate. The following linear model was used for the estimation of breastfeeding indicators:

$$\text{Logit}(P_{x,c,t}) = \beta_0 + \beta_1 SDI_{c,t} + \alpha_c + \gamma_{R[c]} + \omega_{SR[c]} + \varepsilon_{c,t}$$

where $P_{x,c,t}$ is prevalence for breastfeeding category x in country c and year t; $SDI_{c,t}$ is value of the socio-demographic Index for country c and year t; α_c, $\gamma_{R[c]}$, and $\omega_{SR[c]}$ are country, state, region, and super-region random intercepts, respectively.

Following which, a spatio-temporal regression was done using the residuals of the predictions from the linear regression to perform a locally-weighted regression that provides a greater weighting factor to those nearer in space and time. The predicted residuals from this step were then added to those created in the linear regression step.

Finally, a Gaussian Process Regression was done that incorporates the variance of the input data as well as the variance of the model predictions. It used predictions from the spatio-temporal regression as the mean function, and generated draws from a multinomial distribution (based on the data uncertainty in the prior) to generate the final prevalence estimates and their confidence intervals.

Six models were estimated to produce each of the categories: the proportion of currently breastfeeding infants 0-5 months of age, the ratio of infants exclusively breastfed to breastfed infants 0-5 months of age, the ratio of infants predominantly breastfed to breastfed infants 0-5 months of age, the ratio of infants partially breastfed to breastfed infants 0-5 months of age, the proportion of currently breastfeeding infants 6-11 months of age, and the proportion of currently breastfeeding infants 12-23 months of age. The ratios of exclusive, predominant, and partial breastfeeding were converted to the total category prevalence proportions by multiplying each ratio by the estimates of any breastfeeding among infants aged 0-5 months. This ensures that these categories sum correctly to "any breastfeeding 0-5 months" envelope. The proportion of infants receiving no breast milk 0-5 months of age were calculated by subtracting the estimates of current breastfeeding from 1. The same operation was performed to estimate discontinued breastfeeding in the 6-11 months and 12-23 months categories.

The outcomes were included based on the strength of available evidence supporting a causal relationship. Relative risks used for suboptimal breastfeeding were generated based on published review by the WHO.[7] Non-exclusive breastfeeding exposure was paired with diarrhoea and lower respiratory infections as disease outcomes. Discontinued breastfeeding was paired with diarrhoea only.

The standard GBD PAF equation were used to calculate PAFs for non-exclusive breastfeeding and discontinued breastfeeding, and each of their paired outcomes using exposure estimates and relative risks.

Epidemiological evidence for relative risk of various disease outcomes attributable to suboptimal breastfeeding was obtained from the following studies:

Horta BL, Victora CG. Short-term effects of breastfeeding: a systematic review on the benefits of breastfeeding on diarrhoea and pneumonia mortality. World Health Organisation 2013. http://www.who.int/iris/handle/10665/95585.

Olofin I, McDonald CM, Ezzati M, et al. Associations of suboptimal growth with all-cause and cause-specific mortality in children under five years: a pooled analysis of ten prospective studies. *PLoS ONE* 2013; 8: e64636.

Genser B, Strina A, Santos LA, et al. Impact of a city-wide sanitation intervention in a large urban centre on social, environmental and behavioural determinants of childhood diarrhoea: analysis of two cohort studies. *Int J Epidemiol* 2008; 37(4): 831–40.

A.5. High body mass index in children

GBD computes high body-mass index (BMI) in children for age up to 19 years, defining this as being overweight or obese based on Internal Obesity Task Force (IOTF) cut-offs. For the purpose of attributing disease burden to BMI, the TMREL for BMI in children (age up to 19 years), was based on IOTF cut-offs for normal weight. The IOTF cut-offs were preferred over WHO standards because the IOTF cut-offs provide consistent child-specific standards for ages 2-18 years derived from surveys covering multiple countries and were more commonly used in scientific literature covering childhood obesity.

The steps in the estimation of the high body-mass index risk factor in children are shown in the following flowchart:

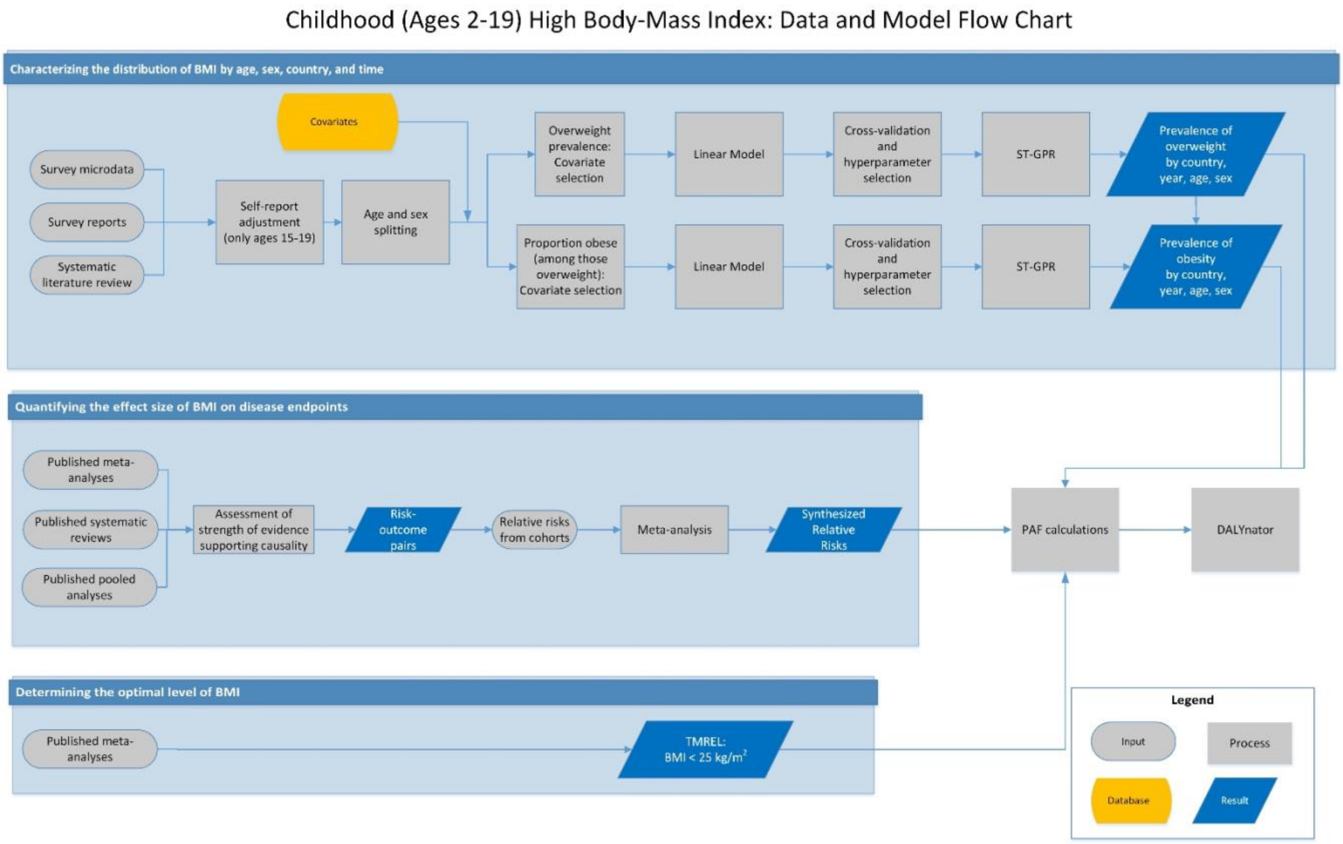

Data

Nationally or sub-nationally representative estimates of overweight prevalence, obesity prevalence, or mean BMI were included from the published literature. The representative studies providing data on mean BMI or prevalence of overweight or obesity among children were also included. For children (2-18 years), studies were included if they used IOTF standards to define overweight and obesity thresholds. Studies were excluded if they used non-random samples (e.g. case-control studies or convenience samples), conducted among specific subpopulations (e.g. pregnant women, racial or ethnic minorities, immigrants, or individuals with specific diseases), used alternative methods to assess adiposity (e.g. waist-circumference, skin-fold thickness, or hydrodensitometry), had sample sizes of less than 20 per age-sex group, or provided inadequate information on any of the inclusion criteria.

Where individual-level survey data were available, mean BMI were computed using weight and height. BMI was then used to determine the prevalence of overweight and obesity. For individuals aged 2-18 years, monthly IOTF cut-offs[2] were used to determine overweight and obese status when age in months was available. When only age in years was available, cut-off for the midpoint of that year were used. Obese individuals were also considered to be overweight. The studies using the WHO standards or country-specific cut-offs to define childhood overweight and obesity were excluded. At the individual-level, BMI<10 kg/m^2 and BMI>70 kg/m^2 were considered to be biologically implausible and these observations were excluded.

Data were extracted from reports and literature on mean BMI, prevalence of overweight, and prevalence of obesity, measures of uncertainty for each, and sample size, by the most granular age and sex-groups available. Additionally, the same study-level covariates were extracted as were extracted from microdata (measurement, urbanicity, and representativeness), as well as location and year.

In addition to the primary indicators described above, relevant survey-design variables, including primary sampling unit, strata, and survey weights were extracted, which were used to tabulate individual-level microdata and produce accurate measures of uncertainty. Three study-level covariates were extracted: 1) whether height and weight data were measured or self-reported; 2) whether the study was predominantly conducted in an urban area, rural area or both; and 3) the level of representativeness of the study (national or sub-national).

Finally, relevant demographic indicators were extracted, including location, year, age, and sex. The standard error of the mean from individual-level data were estimated, where available, and the reported standard error of the mean were used for published data. When multiple data sources were available for the same country, all of them were included in the analysis. If data from the same data source were available in multiple formats such as individual-level data and tabulated data, individual-level data were used.

Both measured and self-reported data were included. The bias was tested in self-reported data compared to measured data, which is considered to be the gold-standard. There was no clear direction of bias for children aged 2-14 years, so for these age groups only measured data were included. For individuals aged 15 years and above, self-reported data were adjusted for in overweight prevalence, obesity prevalence, and mean BMI using the following nested hierarchical mixed-effects regression models fit using restricted maximum likelihood separately by sex:

$$\text{logit}(\text{overweight})_{c,a,t} = \beta_0 + \beta_1 m + \sum_{k=2}^{19} \beta_k I_{A[a]} + \sum_{l=20}^{55} \beta_l I_{A[a]} I_{M[m]} + \alpha_s + \alpha_s m + \alpha_r + \alpha_r m + \alpha_c + \alpha_c m + \alpha_t + \alpha_t m + \epsilon_{c,a,t}$$

$$\text{logit}(\text{obesity})_{c,a,t} = \beta_0 + \beta_1 m + \sum_{k=2}^{19} \beta_k I_{A[a]} + \sum_{l=20}^{55} \beta_l I_{A[a]} I_{M[m]} + \alpha_s + \alpha_s m + \alpha_r + \alpha_r m + \alpha_c + \alpha_c m + \alpha_t + \alpha_t m + \epsilon_{c,a,t}$$

$$\log(\text{BMI})_{c,a,t} = \beta_0 + \beta_1 m + \sum_{k=2}^{19} \beta_k I_{A[a]} + \sum_{l=20}^{55} \beta_l I_{A[a]} I_{M[m]} + \alpha_s + \alpha_s m + \alpha_r + \alpha_r m + \alpha_c + \alpha_c m + \alpha_t + \alpha_t m + \epsilon_{c,a,t}$$

Where m is a fixed effect on measurement (binary, either measured (1) or self-report (0)), $I_{A[a]}$ is an indicator variable for specific age group A, $I_{A[a]} I_{M[m]}$ is an interaction term between age and measurement, α_s, α_r, and α_c are random effects at the super region, region, country, and state, respectively, and α_t is a random effect by time-period (1980–1989, 1990–1999, 2000–2009, and 2010–2017). Random effects at the country-level and time-period level were used to fit the models, but were taken as noise and were not used in adjustment of self-reported data. The uncertainty was propagated in the self-reported adjustment model by adding the variance of each of the regression coefficients used in adjustment to the data variance in delta-transformed space. After adjustment, regressions confirmed that self-reported data was no longer significantly different from measured data.

Any report or literature data provided in age groups wider than the standard 5-year age groups or as both sexes combined were split using the approach used by Ng et al.[12] Briefly, age-sex patterns were identified using sources with data on multiple age-sex groups, and these patterns were applied to split aggregated report and literature data. Uncertainty in the age-sex split was propagated by multiplying the standard error of the data by the square root of the number of splits performed. The uncertainty was not propagated in the age-pattern and sex-pattern used to split the data as they seemed to have small effect.

The major data sources from India were national surveys including National Family Health Surveys, District Level Household Surveys, Annual Health Survey, National Nutrition Monitoring Bureau Diet and Nutritional Surveys, and Rapid Survey on Children.

Modelling strategy

After adjusting for self-report bias and splitting aggregated data into 5-year age-sex groups, ST-GPR were used to estimate the prevalence of overweight and obesity. The linear model, which when added to the smoothed residuals forms the mean prior for GPR is as follows:

$$\mathrm{logit}(\mathrm{overweight})_{c,a,t} = \beta_0 + \beta_1 \mathrm{energy}_{c,t} + \beta_2 \mathrm{SDI}_{c,t} + \beta_3 \mathrm{vehicles}_{c,t} + \beta_4 \mathrm{agriculture}_{c,t} + \sum_{k=5}^{21} \beta_k \mathrm{I}_{A[a]} + \alpha_s + \alpha_r + \alpha_c$$

$$\mathrm{logit}(\mathrm{obesity}/\mathrm{overweight})_{c,a,t} = \beta_0 + \beta_1 \mathrm{energy}_{c,t} + \beta_2 \mathrm{SDI}_{c,t} + \beta_3 \mathrm{vehicles}_{c,t} + \sum_{k=4}^{21} \beta_k \mathrm{I}_{A[a]} + \alpha_s + \alpha_r + \alpha_c$$

where energy is ten-year lag-distributed energy consumption per capita, SDI is a composite index of development including lag-distributed income per capita, education, and fertility, $\mathrm{vehicles}$ is the number of two or four-wheel vehicles per capita, and $\mathrm{agriculture}$ is the proportion of the population working in agriculture. $\mathrm{I}_{A[a]}$ is a dummy variable indicating specific age group A that the prevalence point captures, and α_s, α_r, and α_c are super region, region, country, and state, random intercepts, respectively. Random effects were used in model fitting but were not used in prediction.

All combinations were tested for the following covariates to see which performed best in terms of in-sample AIC for the overweight linear model and the obesity as a proportion of overweight linear model: ten-year lag distributed energy per capita, proportion of the population living in urban areas, SDI, lag distributed income per capita, educational attainment (years) per capita, proportion of the population working in agriculture, grams of sugar adjusted for energy per capita, grams of sugar not adjusted for energy per capita, and the number of two or four-wheeled vehicles per capita. These candidate covariates were selected based on theory as well as reviewing covariates used in other publications. The final linear model was selected based on: 1) if the direction of covariates matched what is expected from theory, 2) all the included covariates were significant, and 3) minimising in-sample AIC. The covariate selection process was performed using the dredge package in R.

The ST-GPR incorporates information about data density into the process for smoothing over space and time. Estimates in areas/years with few observations had more weight on regional observations. To specify the distribution of time weights and space weights, values of lambda=0.2 and zeta=0.05, were used, respectively. The value of omega=1.0 was used for the distribution of age weights. The GPR scale parameter was set to 20, and used the default global cut-off setting for amplitude.

The ensemble distribution approach which fit ensemble weights by source and sex, with source- and sex-specific weights averaged across all sources included to produce the final global weights were used. The ensemble weights were fit on measured microdata. The final ensemble weights were: exponential = 0.002, gamma = 0.028, inverse gamma = 0.085, log-logistic = 0.187, Gumbel = 0.220, Weibull = 0.011, log-normal = 0.058, normal = 0.012, beta = 0.136, mirror gamma = 0.008, and mirror Gumbel = 0.113.

One thousand draws of BMI distributions estimated for each location, year, age group, and sex were produced by fitting an ensemble distribution using 1,000 draws of estimated mean BMI, 1,000 draws of estimated SD, and the ensemble weights. Estimated SD was produced by optimising a SD to fit estimated overweight prevalence draws and estimated obesity prevalence draws.

The relative risk per 5-unit change in BMI for each disease endpoint was obtained from meta-analyses, and where available, pooled analyses of prospective observational studies. In cases where a relative risk per 5-unit change in BMI was not available dose-response meta-analysis were computed using two-step generalised-least squares for time trends estimation methods.

For childhood outcomes (2-19 years), categorical relative risks were computed for overweight and obesity using a random effects meta-analysis.

Risk-outcome pairs were defined based on strength of available evidence supporting a causal effect. The epidemiological evidence on the relative risk outcome-pair from the following study suggests that high body-mass index in children cause asthma:

Mebrahtu TF, Feltbower RG, Greenwood DC, Parslow RC. Childhood body mass index and wheezing disorders: a systematic review and meta-analysis. *Pediatr Allergy Immunol* 2015; 26: 62–72.

B. Projections up to 2030

GBD 2017 produced projections for the health-related Sustainable Development Goal (SDG) indicators up to 2030 based on past trends, using a new advanced modelling framework.[13] The steps used to produce projections for the child and maternal malnutrition indicators are as follows.

For each malnutrition indicator, the annual change from the previous year was first calculated from 1990 to 2017 using the logit of the prevalence for each year. The weight for each year was calculated using this formula:

$$weight_{year} = (t - 1990 + 1)^{\omega}$$

where ω is the weight function, the value of which denotes how much higher impact recent years would have compared with the past years when calculating the annual rate of change for the projection. To determine the appropriate value of ω for each indicator, an out-of-sample predictive validity test was done using data from 1990 to 2007 to predicted values for the years from 2008 to 2017. Assuming a range of values, in the increments of 0.25, from 0 to 10 for *ω*, the best predicted value for the period 2008 to 2017 was tested for each indicator. The final value for the weight function (ω) specific to each indicator for projection was chosen that minimised the root mean squared error in the 2008–2017 projections based on the 1990–2007 data. The weight functions computed for the malnutrition indicators reported in this paper are as follows:

**Malnutrition indicators**	**Weight function**
Low birth weight and short gestation	1.5
Child stunting	1.7
Child wasting	1.9
Child underweight	1.8
Exclusive breastfeeding	0.8
Child overweight	1.7
Anaemia in children 0-4 years	1.0
Anemia in women of reproductive ages	1.4

The inverse of the weighted logit mean of the annualized rate of change from 1991 to 2017 was then applied to the years 2018 onward to estimate the prevalence of each malnutrition indicator up to 2030, which takes into account the trends observed so far.

C. Uncertainty intervals

Within the GBD analytic framework, uncertainty is captured and propagated by sampling 1000 times from the posterior distribution of each estimation step, even in situations where the actual model is run far in excess of 1000 times. The approaches for ensuring convergence of model results was arrived at through iterative testing. GBD has assessed the number of iterations across a wide set of outcomes, and 1000 iterations were found to be quite sufficient to ensure stability across the variables. 1000 samples of each distribution were found to be a sufficient number to allow for accurate propagation of uncertainty into subsequent modelling steps, generating aggregate results, and facilitating secondary analyses, as demonstrated in the results of the diagnostic tests for low birth weight, stunting, and wasting shown on pages 43-48 of this document.

Point estimates for each quantity of interest were derived from the mean of the draws, while 95% (uncertainty intervals (UIs) were derived from the 2.5th and 97.5th percentiles of the 1,000 draw level values. Uncertainty in the estimation is attributable to sample size variability within data sources, different availability of data by age, sex, year, or location, and cause-specific model specifications. The UIs were determined for components of cause-specific estimation based on 1,000 draws from the posterior distribution of cause specific mortality by age, sex, and location for each year included in the GBD 2017 analysis. Similarly, for non-fatal estimates if there was a change in disease estimates between locations or over time that was in the same direction in more than 950 of the 1,000 samples we report it as significant. With this approach, uncertainty could be quantified and propagated into the final quantities of interest.

D. References

1. Katz J, Lee AC, Kozuki N, et al. Mortality risk in preterm and small-for-gestational-age infants in low-income and middle-income countries: a pooled country analysis. *Lancet* 2013; 382: 417–25.
2. World Health Organization. WHO global database on child growth and malnutrition. WHO. http://www.who.int/nutgrowthdb/en/.
3. Wang Y, Chen H-J. Use of Percentiles and Z-scores in Anthropometry. In: Preedy VR, ed. Handbook of anthropometry. New York: Springer, 2012: 29–48.
4. Uribe MCA, Gaviria AL, Restrepo AE. Concordance between Z-scores from WHO 2006 and the NCHS 1978 growth standards of children younger than five. Antioquia-Colombia. *Perspect Nut Hum* 2008; 10: 177–87.
5. Olofin I, McDonald CM, Ezzati M, et al. Associations of suboptimal growth with all-cause and cause-specific mortality in children under five years: a pooled analysis of ten prospective studies. *PLoS One* 2013; 8: e64636.
6. McDonald CM, Olofin I, Flaxman S, et al. The effect of multiple anthropometric deficits on child mortality: meta-analysis of individual data in 10 prospective studies from developing countries. *Am J Clin Nutr* 2013; 97: 896–901.
7. Horta BL, Victora CG. Short-term effects of breastfeeding: a systematic review on the benefits of breastfeeding on diarrhoea and pneumonia mortality. World Health Organization 2013. http://www.who.int/iris/handle/10665/95585
8. Centers for Disease Control and Prevention. Iron deficiency–United States, 1999–2000. *Morb Mortal Wkly Rep* 2002; 51: 897–9.
9. Looker AC, Dallman PR, Carroll MD, Gunter EW, Johnson CL. Prevalence of iron deficiency in the united states. *JAMA* 1997; 277: 973–6.
10. Semba RD, Bloem MW. The anemia of vitamin A deficiency: epidemiology and pathogenesis. *Eur J Clin Nutr* 2002; 56: 271–81.
11. Cole TJ, Lobstein T. Extended international (IOTF) body mass index cut-offs for thinness, overweight and obesity. *Pediatr Obes* 2012; 7: 284–94.
12. Ng M, Fleming T, Robinson M, et al. Global, regional, and national prevalence of overweight and obesity in children and adults during 1980–2013: a systematic analysis for the Global Burden of Disease Study 2013. *Lancet* 2014; 384: 766–81.
13. Foreman KJ, Marquez N, Dolgert A, et al. Forecasting life expectancy, years of life lost, all-cause and cause-specific mortality for 250 causes of death: reference and alternative scenarios for 2016–2040 for 195 countries and territories. *Lancet* 2018; 392: 2052–90.

2. GBD 2017 India data inputs for child and maternal malnutrition exposure, covariates, and the disease burden and deaths attributable to malnutrition

Abel R, Rajaratnam J, Gnanasekaran VJ, Jayaraman P. Prevalence of anaemia and iron deficiency in three trimesters in Rural Vellore district, South India. Trop Doct. 2001; 31:86-9.
Abel R, Rajaratnam J, Kalaimani A, Kirubakaran S. Can iron status be improved in each of the three trimesters? A community-based study. Eur J Clin Nutr. 2000; 54:490-3.
Abel R, Sampathkumar V. Tamil Nadu nutritional survey comparing children aged 0-3 years with the NCHS/CDC reference population. Indian J Pediatr. 1998; 65:565-72.
Agarwal AK, Sen AK, Kalra NK, Gupta N. Prevalence of anaemia during pregnancy in district Burdwan, West Bengal. Indian J Public Health. 1999; 43:26-31.
Agarwal D, Pandey C, Agarwal K. Vitamin A administration and preschool child mortality. Nutr Res. 1995; 15:669–80. as it appears in Imdad A, Herzer K, Mayo-Wilson E, Yakoob MY, Bhutta ZA. Vitamin A supplementation for preventing morbidity and mortality in children from 6 months to 5 years of age. Cochrane Database Syst Rev. 2010; CD008524.
Agarwal DK, Agarwal KN, Tripathi AM. Nutritional status in rural pregnant women of Bihar and Uttar Pradesh. Indian Pediatr. 1987; 24:119-25.
Agarwal DK, Bhardwaj B, Singla PN, Tripathi AM, Agarwal KN. Etiology of maternal and early childhood deficiency anemia. Indian J Pediatr. 1986; 53:389-95.
Agarwal KN, Agarwal DK, Mishra KP. Impact of anaemia prophylaxis in pregnancy on maternal haemoglobin, serum ferritin & birth weight. Indian J Med Res. 1991; 277-80.
Agarwal KN, Gomber S, Bisht H, Som M. Anemia prophylaxis in adolescent school girls by weekly or daily iron-folate supplementation. Indian Pediatr. 2003; 40:296-301.
Ahankari A, Bapat S, Myles P, Fogarty A, Tata L. Factors associated with preterm delivery and low birth weight: a study from rural Maharashtra, India. F1000Res. 2017; 6:72.
Arya S, Chellani H, Pandey J. Evaluation of safety of oral vitamin "A" megadose co-administered with measles vaccination. Indian Pediatr. 2000; 37:1341–7. as it appears in Imdad A, Herzer K, Mayo-Wilson E, Yakoob MY, Bhutta ZA. Vitamin A supplementation for preventing morbidity and mortality in children from 6 months to 5 years of age. Cochrane Database Syst Rev. 2010; CD008524.
Awasthi S, Das R, Verma T, Vir S. Anemia and undernutrition among preschool children in Uttar Pradesh, India. Indian Pediatr. 2003; 40:985-90.
Awasthi S, Pande VK. Cause-specific mortality in under fives in the urban slums of Lucknow, north India. J Trop Pediatr. 1998; 44:358-61.
Awasthi S, Peto R, Read S, Clark S, Pande V, Bundy D, DEVTA (Deworming and Enhanced Vitamin A) team. Vitamin A supplementation every 6 months with retinol in 1 million pre-school children in north India: DEVTA, a cluster-randomised trial. Lancet. 2013; 381:1469-77.
Bahl R, Frost C, Kirkwood BR, Edmond K, Martines J, Bhandari N, Arthur P. Infant feeding patterns and risks of death and hospitalization in the first half of infancy: multicentre cohort study. Bull World Health Organ. 2005; 83:418-26.
Bains K, Mann SK. Physical Fitness in Relation to Energy and Iron Status of Female College Students. Food Nutr Bull. 2000; 21:305–10.
Bamji MS, V S Murthy PV, Williams L, Vardhana Rao MV. Maternal nutritional status & practices & perinatal, neonatal mortality in rural Andhra Pradesh, India. Indian J Med Res. 2008; 127:44-51.
Bang AT, Bang RA, Baitule SB, Reddy MH, Deshmukh MD. Effect of home-based neonatal care and management of sepsis on neonatal mortality: field trial in rural India. Lancet. 1999; 354:1955-61.
Bang AT, Bang RA, Reddy HM, Deshmukh MD, Baitule SB. Reduced incidence of neonatal morbidities: effect of home-based neonatal care in rural Gadchiroli, India. J Perinatol. 2005; 25 (Suppl 1): S51-61.
Baruah J, Kusre G, Bora R. Pattern of Gross Congenital Malformations in a Tertiary Referral Hospital in Northeast India. Indian J Pediatr. 2015; 82:917–22.
Basavanagowdappa H, Guruswamy M, Ravi MD, Mahesh PA. JSS Hospital inpatient data 2014-2017. JSS Hospital, Mysuru, Karnataka, India.
Bhandari N, Mazumder S, Taneja S, Sommerfelt H, Strand TA. Effect of implementation of Integrated Management of Neonatal and Childhood Illness (IMNCI) programme on neonatal and infant mortality: cluster randomised controlled trial. BMJ. 2012; 344: e1634.
Bharati P, Ghosh R, Gupta R. Socioeconomic Condition and Anaemia among the Mahishya Population of Southern West Bengal, India. Malays J Nutr. 2004; 10:23-30.

Bhargava M, Iyer PU, Kumar R, Ramji S, Kapani V, Bhargava SK. Relationship of maternal serum ferritin with foetal serum ferritin, birth weight and gestation. J Trop Pediatr. 1991; 37:149-52.

Bhargava SK, Singh KK, Saxena BN, India Council of Medical Research (ICMR). ICMR Task Force National Collaborative Study on Identification of High Risk Families, Mothers and Outcome of their Off-springs with particular reference to the problem of maternal nutrition, low birth weight, perinatal and infant morbidity and mortality in rural and urban slum communities. Summary, conclusions and recommendations. 1991; 28:1473-80.

Bisai S, Mahalanabis D, Sen A, Bose K, Datta N. Maternal early second trimester pregnancy weight in relation to birth outcome among Bengalee Hindus of Kolkata, India. Ann Hum Biol. 2007; 34:91-101.

Biswas R, Biswas AB, Manna B, Bhattacharya SK, Dey R, Sarkar S. Effect of vitamin A supplementation on diarrhoea and acute respiratory tract infection in children. A double blind placebo controlled trial in a Calcutta slum community. Eur J Epidemiol. 1994; 10: 57–61. as it appears in Imdad A, Herzer K, Mayo-Wilson E, Yakoob MY, Bhutta ZA. Vitamin A supplementation for preventing morbidity and mortality in children from 6 months to 5 years of age. Cochrane Database Syst Rev. 2010; CD008524.

Blencowe H, Cousens S, Jassir FB, Say L, Chou D, Mathers C, Hogan D, Shiekh S, Qureshi ZU, You D, Lawn JE; Lancet Stillbirth Epidemiology Investigator Group. National, regional, and worldwide estimates of stillbirth rates in 2015, with trends from 2000: a systematic analysis. Lancet Glob Health. 2016; 4:e98-108.

Brabin L, Nicholas S, Gogate A, Gogate S, Karande A. High prevalence of anaemia among women in Mumbai, India. Food Nutr Bull. 1998; 19:205-9.

Brahmam G, Madhavan NK, Laxmaiah A, Reddy C, Ranganathan S, Vishnuvardhana Rao M, Nadamuni Naidu A, Vijayaraghavan K, Sivakumar B, Kamala Krishnaswamy, Gowarinth Sastry J, Mohan Ram M, Praldad Rao N, Vinodini R. Community Trials with Iron and Iodine Fortified Salt (Double Fortified Salt). In: Geertman RM, ed. Proceedings of the 8th World Salt Symposium. Amsterdam, The Netherlands: Elsevier; 2000. 955-60.

Center for Vaccine Development, University of Maryland, Centers for Disease Control and Prevention, Department of Medical Microbiology and Immunology, Göteborg University, International Vaccine Institute, National Institute of Cholera and Enteric Diseases, Perry Point Cooperative Studies Program Coordinating Center, U.S. Department of Veterans Affairs, School of Medicine, University of Virginia, University of Chile. India - Kolkata Global Enteric Multicenter Study 2007-2011. Baltimore, MD, United States: Center for Vaccine Development, University of Maryland.

Center for Vaccine Development, University of Maryland, Centers for Disease Control and Prevention, Department of Medical Microbiology and Immunology, Göteborg University, International Vaccine Institute, National Institute of Cholera and Enteric Diseases, Perry Point Cooperative Studies Program Coordinating Center, U.S. Department of Veterans Affairs, School of Medicine, University of Virginia, University of Chile. India - Kolkata Global Enteric Multicenter Study 2011-2013. Baltimore, United States: Center for Vaccine Development, University of Maryland.

Centre for Research on the Epidemiology of Disasters (CRED), Catholic University of Leuven. EM-DAT: The OFDA/CRED International Disaster Database. Brussels, Belgium: Catholic University of Leuven.

Chakraborty B, Bhattacharya AR. Xerophthalmia in under-five children in West Bengal. Indian Pediatr. 1987; 24:600-2.

Chaturvedi S, Kapil U, Gnanasekaran N, Sachdev HP, Pandey RM, Bhanti T. Nutrient intake amongst adolescent girls belonging to poor socioeconomic group of rural area of Rajasthan. Indian Pediatr. 1996; 33:197-201.

Chiplonkar S, Agte V, Mengale S. Relative importance of micronutrient deficiencies in iron deficiency anemia. Nutr Res. 2003; 23:1355-67.

Chowdhury S, Kumar R, Ganguly NK, Kumar L, Walia BNS. Effect of vitamin A supplementation on childhood morbidity and mortality. Indian J Med Sci. 2002; 56:259-64. as it appears in Imdad A, Herzer K, Mayo-Wilson E, Yakoob MY, Bhutta ZA. Vitamin A supplementation for preventing morbidity and mortality in children from 6 months to 5 years of age. Cochrane Database Syst Rev. 2010; CD008524.

Christian Medical College - Vellore, Fogarty International Center, National Institutes of Health, Foundation for the National Institutes of Health. India - Vellore Malnutrition and Enteric Disease (MAL-ED) Study 2009-2014. [Data shared for this analysis]

Dahiya S, Kapoor AC. Anaemia in women in a selected rural area of Haryana: effect of dietary intake level. Indian J Nutr Diet. 1995; 224-8.

Das S, Bapat U, More NS, Alcock G, Fernandez A, Osrin D. Nutritional status of young children in Mumbai slums: a follow-up anthropometric study. Open Nutr J. 2012; 11:100.

Department of Women and Child Development, Ministry of Human Resource Development, Government of India, United Nations Children's Fund (UNICEF) - India Country Office. India Summary Report on the Multiple Indicator Cluster Survey 2000. New Delhi, India: UNICEF India Country Office; 2000.

Department of Women and Child Development, Ministry of Human Resource Development, Government of India, United Nations Children's Fund (UNICEF) - India Country Office. India Summary Report on the Multiple Indicator Cluster Survey 2000. New Delhi, India: UNICEF India Country Office; 2000.

Department of Women and Child Development, Ministry of Human Resource Development, Government of India. India Nutrition Profile Survey 1995-1996. New Delhi, India: Ministry of Human Resource Development.

Desai S, Reeve V. National Council of Applied Economic Research, University of Michigan. India Human Development Survey 2005. Ann Arbor, Michigan: Inter-University Consortium for Political and Social Research.
Desai S, Reeve V. National Council of Applied Economic Research, University of Michigan. India Human Development Survey 2011-12. Ann Arbor, Michigan: Inter-University Consortium for Political and Social Research.
Deshmukh V, Lahariya C, Krishnamurthy S, Das MK, Pandey RM, Arora NK. Taken to Health Care Provider or Not, Under-Five Children Die of Preventable Causes: Findings from Cross-Sectional Survey and Social Autopsy in Rural India. Indian J Community Med. 2016; 41:108–19.
Directorate of Economics & Statistics and Office of Chief Registrar (Births & Deaths), Government of National Capital Territory of Delhi. Report on Medical Certification of Cause of Deaths in Delhi-2013. New Delhi, India: Directorate of Economics & Statistics and Office of Chief Registrar (Births & Deaths); 2014.
Fakhir S, Srivastava I, Ahmad P, Hasan SS. Prevalence of xerophthalmia in pre-school children in an urban slum. Indian Pediatr. 1993; 30:668-70.
Fall CHD, Sachdev HS, Osmond C, Lakshmy R, Biswas SD, Prabhakaran D, Tandon N, Ramji S, Reddy KS, Barker DJP, Bhargava SK. Adult metabolic syndrome and impaired glucose tolerance are associated with different patterns of BMI gain during infancy: Data from the New Delhi Birth Cohort. Diabetes Care. 2008; 31:2349-56.
FAO Supply Utilization Accounts 1961-2013. Personal Correspondence with Dr. Josef Schmidhuber, 2016. [Data shared for this analysis]
Feldon K, Bahl S, Bhatnagar P, Wenger J. Severe vitamin A deficiency in India during pulse polio immunization. Indian J Med Res. 2005; 122:265-7.
Food and Agriculture Organization of the United Nations (FAO). FAOSTAT Food Balance Sheets, October 2014. Rome, Italy: FAO.
Ford Foundation, Nutrition Foundation of India. India Profiles of Undernutrition and Underdevelopment: Studies of Poor Communities in Seven Regions of the Country. New Delhi, India: Nutrition Foundation of India. (Scientific Report Series No. 8).
Genser B, Strina A, Santos LA, et al. Impact of a city-wide sanitation intervention in a large urban centre on social, environmental and behavioural determinants of childhood diarrhoea: analysis of two cohort studies. Int J Epidemiol 2008; 37:831–40.
George K, Prasad J, Singh D, Minz S, Albert DS, Muliyil J, Joseph KS, Jayaraman J, Kramer MS. Perinatal outcomes in a South Asian setting with high rates of low birth weight. BMC Pregnancy Childbirth. 2009 Feb; 9:5.
Ghosh R, Bharati P. Haemoglobin status of adult women of two ethnic groups living in a peri-urban area of Kolkata city, India: a micro-level study. Asia Pac J Clin Nutr. 2003; 12:451-9.
Ghosh SK, Yadav RS, Das BS, Sharma VP. Influence of nutritional and haemoglobin status on malaria infection in children. Indian J Pediatr. 1995; 62:321-6.
Global Burden of Disease Health Financing Collaborator Network, Institute for Health Metrics and Evaluation (IHME). Global Development Assistance for Health, Government, Prepaid Private, and Out-of-Pocket Health Spending 1995-2014. Seattle, United States: IHME; 2017.
Gomber S, Kumar S, Rusia U, Gupta P, Agarwal KN, Sharma S. Prevalence & etiology of nutritional anaemias in early childhood in an urban slum. Indian J Med Res. 1998; 107: 269-73.
Goudar SS, Goco N, Somannavar MS, Vernekar SS, Mallapur AA, Moore JL, Wallace DD, Sloan NL, Patel A, Hibberd PL, Koso-Thomas M, McClure EM, Goldenberg RL. Institutional deliveries and perinatal and neonatal mortality in Southern and Central India. Reprod Health. 2015; 12(Suppl 2): S13.
Government of Orissa, Micronutritent Initiative, United Nations Children's Fund, World Health Organization.Orissa Impact of Vitamin A Supplementation Delivered with Oral Polio Vaccine as Part of Immunization Campaign [Draft final report]. Bhubheshwar, India: Government of Orissa; 2001.
Gupta PC, Subramoney S, Sreevidya S. Smokeless tobacco use, birth weight, and gestational age: population based, prospective cohort study of 1217 women in Mumbai, India. BMJ. 2004; 328:1538.
Gupta V, Shukla K. Epidemiology of anaemia in pre-school children from a rural and a slum community of Varanasi. Indian J Prev Soc Med. 1985; 85-9.
Gupte SC, Shaw AN, Shah KC. Hematological findings and severity of G6PD deficiency in Vataliya Prajapati subjects. J Assoc Physicians India. 2005; 53: 1027–30.
Health Management Information System (HMIS), National Health Mission, Ministry of Health and Family Welfare, Government of India. India Performance Related to Immunisation 2007-2009. New Delhi, India: Ministry of Health and Family Welfare.
Health Management Information System (HMIS), National Health Mission, Ministry of Health and Family Welfare, Government of India. India Performance Related to Immunisation 2008-2010. New Delhi, India: Ministry of Health and Family Welfare.
Health Management Information System (HMIS), National Health Mission, Ministry of Health and Family Welfare, Government of India. India Performance Related to Immunisation 2009-2011. New Delhi, India: Ministry of Health and Family Welfare.

Health Management Information System (HMIS), National Health Mission, Ministry of Health and Family Welfare, Government of India. India Performance Related to Immunisation 2010-2012. New Delhi, India: Ministry of Health and Family Welfare.
Health Management Information System (HMIS), National Health Mission, Ministry of Health and Family Welfare, Government of India. India Performance Related to Immunisation 2011-2013. New Delhi, India: Ministry of Health and Family Welfare.
Health Management Information System (HMIS), National Health Mission, Ministry of Health and Family Welfare, Government of India. India Performance Related to Immunisation 2012-2014. New Delhi, India: Ministry of Health and Family Welfare.
Health Management Information System (HMIS), National Health Mission, Ministry of Health and Family Welfare, Government of India. India Performance Related to Immunisation 2013-2015. New Delhi, India: Ministry of Health and Family Welfare.
Health Management Information System (HMIS), National Health Mission, Ministry of Health and Family Welfare, Government of India. India Performance Related to Immunisation 2014-2016. New Delhi, India: Ministry of Health and Family Welfare.
Health Management Information System (HMIS), National Health Mission, Ministry of Health and Family Welfare, Government of India. India Performance Related to Immunisation 2015-2017. New Delhi, India: Ministry of Health and Family Welfare.
Hebert JR, Gupta PC, Bhonsle RB, Sinor PN, Mehta H, Mehta FS. Development and testing of a quantitative food frequency questionnaire for use in Gujarat, India. Public Health Nutr. 1999; 2:39–50.
Hirve S, Ganatra B. A prospective cohort study on the survival experience of under five children in rural western India. Indian Pediatr. 1997; 34:995-1001.
Horta BL, Victora CG, Organization WH. Short-term effects of breastfeeding: a systematic review on the benefits of breastfeeding on diarrhoea and pneumonia mortality. World Health Organization; 2013 https://apps.who.int/iris/handle/10665/95585 (accessed April 15, 2019)
India Neonatal Preterm Birth Complications Data 1995 as provided by the Global Burden of Disease 2010 Neonatal Conditions Expert Group. [Data shared for this analysis]
Indian Council of Medical Research (ICMR). India Study on Causes of Death by Verbal Autopsy 2003. New Delhi, India: ICMR. [Data shared for this analysis]
International Institute for Population Sciences (IIPS), Ministry of Health and Family Welfare, Government of India, ICF. India National Family Health Survey (NFHS-4) 2015-2016: National and State-level Factsheets. Mumbai, India: IIPS.
International Institute for Population Sciences (IIPS), Ministry of Health and Family Welfare, Government of India, Macro International. India National Family Health Survey (NFHS-1) 1992-1993: National Report. Mumbai, India: IIPS.
International Institute for Population Sciences (IIPS), Ministry of Health and Family Welfare, Government of India, Macro International. India National Family Health Survey (NFHS-3) 2005-2006: National and State Reports. Mumbai, India: IIPS.
International Institute for Population Sciences (IIPS), Ministry of Health and Family Welfare, Government of India, Macro International. India National Family Health Survey Data (NFHS-1) 1992-1993. Mumbai, India: IIPS.
International Institute for Population Sciences (IIPS), Ministry of Health and Family Welfare, Government of India, Macro International. India National Family Health Survey Data (NFHS-3) 2005-2006. Mumbai, India: IIPS.
International Institute for Population Sciences (IIPS), Ministry of Health and Family Welfare, Government of India, ORC Macro. India National Family Health Survey (NFHS-2) 1998-1999: National and State Reports. Mumbai, India: IIPS.
International Institute for Population Sciences (IIPS), Ministry of Health and Family Welfare, Government of India, ORC Macro. India National Family Health Survey Data (NFHS-2) 1998-1999. Mumbai, India: IIPS.
International Institute for Population Sciences (IIPS), Ministry of Health and Family Welfare, Government of India. India District Level Household and Facility Survey (DLHS-3) 2007-2008: National and State Reports. Mumbai, India: IIPS.
International Institute for Population Sciences (IIPS), Ministry of Health and Family Welfare, Government of India. India District Level Household and Facility Survey Data (DLHS-3) 2007-2008. Mumbai, India: IIPS.
International Institute for Population Sciences (IIPS), Ministry of Health and Family Welfare, Government of India. India District Level Household and Facility Survey Data (DLHS-4) 2012-2013: State Reports. Mumbai, India: IIPS.
International Institute for Population Sciences (IIPS), Ministry of Health and Family Welfare, Government of India. India District Level Household Survey (DLHS-1) 1998-1999: National Report. Mumbai, India: IIPS.
International Institute for Population Sciences (IIPS), Ministry of Health and Family Welfare, Government of India. India District Level Household Survey (DLHS-2) 2002-2004: National and State Reports. Mumbai, India: IIPS.
International Institute for Population Sciences (IIPS), Ministry of Health and Family Welfare, Government of India. India District Level Household Survey Data (DLHS-1) 1998-1999. Mumbai, India: IIPS.

International Institute for Population Sciences (IIPS), Ministry of Health and Family Welfare, Government of India. India District Level Household Survey Data (DLHS-2) 2002-2004. Mumbai, India: IIPS.

International Institute for Population Sciences, Ministry of Health and Family Welfare, Government of India. India District Level Household and Facility Survey Data (DLHS-4) 2012-2013. Mumbai, India: IIPS.

International Institute for Population Sciences, World Health Organization. India World Health Survey 2003.

International Social Survey Programme (ISSP), GESIS - Leibniz Institute of Social Sciences. ISSP: Family and Changing Gender Roles IV, ZA5900 (Version 4.0.0). Mannheim, Germany: GESIS - Leibniz Institute of Social Sciences; 2012.

Jain DL, Sarathi V, Upadhye D, Gulhane R, Nadkarni AH, Ghosh K, Colah RB. Newborn screening shows a high incidence of sickle cell anemia in Central India. Hemoglobin. 2012; 36:316–22.

Jain S, Chopra H, Garg SK, Bhatnagar M, Singh JV. Anemia in children: early iron supplementation. Indian J Pediatr. 2000; 67:19-21.

Kameswaran C, Bhatia BD, Bhat BV, Oumachigui A. Perinatal mortality: a hospital based study. Indian Pediatr. 1993; 30:997-1001.

Kapil U, Pathak P, Tandon M, Singh C, Pradhan R, Dwivedi SN. Micronutrient deficiency disorders amongst pregnant women in three urban slum communities of Delhi. Indian Pediatr. 1999; 36:983-9.

Kapoor D, Agarwal KN, Sharma S, Kela K, Kaur I. Iron status of children aged 9-36 months in an urban slum Integrated Child Development Services project in Delhi. Indian Pediatr. 2002; 39:136-44.

Kapoor G, Aneja S. Nutritional disorders in adolescent girls. Indian Pediatr. 1992; 29:969-73.

Katz J, Lee AC, Kozuki N, et al. Mortality risk in preterm and small-for-gestational-age infants in low-income and middle-income countries: a pooled country analysis. Lancet 2013; 382: 417–25.

Kaushik SL, Parmar VR, Grover N, Kaushik R. Neonatal mortality rate: relationship to birth weight and gestational age. Indian J Pediatr. 1998; 65:429-33.

Khandait DW, Ambadekar NN, Zodpey SP, Vasudeo ND. Maternal age as a risk factor for stillbirth. Indian J Public Health. 2000; 44: 28-30.

King George's Medical University. King George's Medical University pediatric inpatient data 2017. King George's Medical University, Lucknow, Uttar Pradesh, India.

Kodkany BS, Derman RJ, Honnungar NV, Tyagi NK, Goudar SS, Mastiholi SC, Moore JL, McClure EM, Sloan N, Goldenberg RL. Establishment of a Maternal Newborn Health Registry in the Belgaum District of Karnataka, India. Reprod Health. 2015; 12 Suppl 2: S3.

Koen M, Lemson M, Sampathkumar V, Abel R. Prevalence of anaemia among pregnant mothers in a rural South Indian population. J Obstet Gynaecol India. 1992; 42:283-7.

Kumar CSV, Anand Kumar H, Sunita V, Kapur I. Prevalence of anemia and worm infestation in school going girls at Gulbargha, Karnataka. Indian Pediatr. 2003; 40:70-2.

Kumar V, Datta N, Wadhwa SS, Singhi S. Morbidity and mortality in diarrhea in rural Haryana Indian. Indian J Pediatr. 1985; 52:455-61.

Luwang N, Gupta V. Anaemia in pregnancy in a rural community-influence of dietary intake in the multifactorial aetiology. Indian J Nutr Diet. 1980; 17:414-7.

Mahapatra A, Geddam JJ, Marai N, Murmu B, Mallick G, Bulliyya G, Acharya AS, Satyanarayana K. Nutritional status of preschool children in the drought affected Kalahandi district of Orissa. Indian J Med Res. 2000; 111: 90-4.

Mahavarkar SH, Madhu CK, Mule VD. A comparative study of teenage pregnancy. J Obstet Gynaecol. 2008; 28:604-7.

Mathad V, Metgud C, Mallapur MD. Nutritional status of under-fives in rural area of South India. Indian J Med Sci. 2011; 65:151-6.

Max Planck Institute for Demographic Research, Vienna Institute of Demography. Human Fertility Collection - Total Fertility Rate and Mean Age at Birth. Rostock, Germany: Max Planck Institute for Demographic Research.

McClure EM, Pasha O, Goudar SS, Chomba E, Garces A, Tshefu A, Althabe F, Esamai F, Patel A, Wright LL, Moore J, Kodkany BS, Belizan JM, Saleem S, Derman RJ, Carlo WA, Hambidge KM, Buekens P, Liechty EA, Bose C, Koso-Thomas M, Jobe AH, Goldenberg RL; Global Network Investigators. Epidemiology of stillbirth in low-middle income countries: A Global Network Study. Acta Obstet Gynecol Scand. 2011; 90:1379-85.

Mebrahtu TF, Feltbower RG, Greenwood DC, Parslow RC. Childhood body mass index and wheezing disorders: a systematic review and meta-analysis. Pediatr Allergy Immunol 2015; 26: 62–72.

Mehrotra SK, Mathur JS, Maheshwari BB. Epidemiological aspects of nutritional anaemia in children below five years. Indian J Pediatr. 1976; 43:132-5.

Meshram II, Arlappa N, Balakrishna N, Laxmaiah A, Mallikarjun Rao K, Gal Reddy C, Ravindranath M, Sharad Kumar S, Brahmam GNV. Prevalence and determinants of undernutrition and its trends among pre-school tribal children of Maharashtra State, India. J Trop Pediatr. 2012; 58:125-32.

Meshram II, Arlappa N, Balakrishna N, Mallikharjuna Rao K, Laxmaiah A, Brahmam GN. Trends in the prevalence of undernutrition, nutrient & food intake and predictors of undernutrition among under five year tribal children in India. Asia Pac J Clin Nutr. 2012; 21: 568-76.

Meshram II, Balakrishna N, Arlappa N, Rao KM, Laxmaiah A, Brahmam GNV. Prevalence of Undernutrition, Its Determinants, and Seasonal Variation Among Tribal Preschool Children of Odisha State, India. Asia Pac J Public Health. 2014; 26:470-80.

Meshram II, Kodavanti MR, Chitty GR, Manchala R, Kumar S, Kakani SK, Kodavalla V, Avula L, Ginnela Narsimhachary Veera B. Influence of Feeding Practices and Associated Factors on the Nutritional Status of Infants in Rural Areas of Madhya Pradesh State, India. Asia Pac J Public Health. 2015; 27:NP1345-61.

Meshram II, Laxmaiah A, Venkaiah K, Brahmam GNV. Impact of feeding and breastfeeding practices on the nutritional status of infants in a district of Andhra Pradesh, India. Natl Med J India. 2012; 25:2016.

Ministry of Health and Family Welfare, Government of India, United Nations Children's Fund. India Coverage Evaluation Survey 2005.

Ministry of Health and Family Welfare, Government of India, United Nations Children's Fund. India Coverage Evaluation Survey 2007.

Ministry of Health and Family Welfare, Government of India, United Nations Children's Fund. India Coverage Evaluation Survey Data 2009-2010. [Data shared for this analysis]

Ministry of Statistics and Programme Implementation, Government of India. Common Property Resources, Sanitation & Hygiene Services, National Sample Survey Round 54, January - June 1998. New Delhi, India: Ministry of Statistics and Programme Implementation.

Ministry of Statistics and Programme Implementation, Government of India. Consumer Expenditure Survey, National Sample Survey Round 60, January-June 2004. New Delhi, India: Ministry of Statistics and Programme Implementation.

Ministry of Statistics and Programme Implementation, Government of India. Consumer Expenditure Survey, National Sample Survey Round 64, July 2007- June 2008. New Delhi, India: Ministry of Statistics and Programme Implementation.

Ministry of Statistics and Programme Implementation, Government of India. Employment and Unemployment Survey, India National Sample Survey Round 55, July 1999-June 2000. New Delhi, India: Ministry of Statistics and Programme Implementation.

Ministry of Statistics and Programme Implementation, Government of India. Employment and Unemployment Survey, National Sample Survey Round 38, January-December 1983. New Delhi, India: Ministry of Statistics and Programme Implementation.

Ministry of Statistics and Programme Implementation, Government of India. Employment and Unemployment Survey, National Sample Survey Round 43, July 1987- June 1988. New Delhi, India: Ministry of Statistics and Programme Implementation.

Ministry of Statistics and Programme Implementation, Government of India. Employment and Unemployment Survey, National Sample Survey Round 50, July 1993- June 1994. New Delhi, India: Ministry of Statistics and Programme Implementation.

Ministry of Statistics and Programme Implementation, Government of India. Employment and Unemployment Survey, National Sample Survey Round 61, July 2004- June 2005. New Delhi, India: Ministry of Statistics and Programme Implementation.

Ministry of Statistics and Programme Implementation, Government of India. Household expenditure on services and durable goods survey, National Sample Survey Round 72, July 2014 - June 2015. New Delhi, India: Ministry of Statistics and Programme Implementation.

Ministry of Statistics and Programme Implementation, Government of India. India State Series: Gross State Domestic Product at Factor Cost by Industry of Origin Tables 1980-81 to 1993-94. New Delhi, India: Ministry of Statistics and Programme Implementation.

Ministry of Statistics and Programme Implementation, Government of India. India State Series: Gross State Domestic Product at Factor Cost by Industry of Origin Tables 1993-94 to 2004-05. New Delhi, India: Ministry of Statistics and Programme Implementation.

Ministry of Statistics and Programme Implementation, Government of India. India State Series: Gross State Domestic Product at Factor Cost by Industry of Origin Tables 2004-05 to 2013-14. New Delhi, India: Ministry of Statistics and Programme Implementation.

Ministry of Statistics and Programme Implementation, Government of India. Survey on Healthcare, National Sample Survey Round 52, July 1995-June 1996. New Delhi, India: Ministry of Statistics and Programme Implementation.

Ministry of Statistics and Programme Implementation, Government of India. Survey on Literacy and Culture, National Sample Survey Round 47, July - December 1991. New Delhi, India: Ministry of Statistics and Programme Implementation.

Ministry of Statistics and Programme Implementation, Government of India. Survey on Participation in Education, National Sample Survey Round 42, July 1986- June 1987. New Delhi, India: Ministry of Statistics and Programme Implementation.

Ministry of Women and Child Development, Government of India, United Nations Children's Fund. India Rapid Survey on Children 2013-2014. New Delhi, India: Ministry of Women and Child Development.

Mony PK, Varghese B, Thomas T. Estimation of perinatal mortality rate for institutional births in Rajasthan state, India, using capture-recapture technique. BMJ Open. 2015; 5:e005966.

Nadiger HA, Krishnamachari KA, Naidu AN, Rao BS, Srikantia SG. The use of common salt (sodium chloride) fortified with iron to control anaemia: results of a preliminary study. Br J Nutr. 1980; 43:45-51.

Nallam NR, Paul I, Gnanamani G. Anemia and hypoalbuminia as an adjunct to soil-transmitted helminthiasis among slum school children in Visakhapatnam, South India. Asia Pac J Clin Nutr. 1998; 7:164–9.

National Nutrition Monitoring Bureau, National Institute of Nutrition (NIN), Indian Council of Medical Research. India Micronutrient Deficiency Survey Data 2003-2006. [Data shared for this analysis]

National Nutrition Monitoring Bureau, National Institute of Nutrition (NIN), Indian Council of Medical Research. India Micronutrient Deficiency Survey Report 2003-2006. Hyderabad, India: NIN.

National Nutrition Monitoring Bureau, National Institute of Nutrition (NIN), Indian Council of Medical Research. India Rural First Repeat Survey of Diet and Nutritional Status Data 1988-1990. [Data shared for this analysis]

National Nutrition Monitoring Bureau, National Institute of Nutrition (NIN), Indian Council of Medical Research. India Rural First Repeat Survey of Diet and Nutritional Status Report 1988-1990. Hyderabad, India: NIN.

National Nutrition Monitoring Bureau, National Institute of Nutrition (NIN), Indian Council of Medical Research. India Rural Second Repeat Survey of Diet and Nutritional Status Data 1996-1997. [Data shared for this analysis]

National Nutrition Monitoring Bureau, National Institute of Nutrition (NIN), Indian Council of Medical Research. India Rural Second Repeat Survey of Diet and Nutritional Status Report 1996-1997. Hyderabad, India: NIN.

National Nutrition Monitoring Bureau, National Institute of Nutrition (NIN), Indian Council of Medical Research. India Rural Survey of Diet and Nutritional Status Data 1994-1995. [Data shared for this analysis]

National Nutrition Monitoring Bureau, National Institute of Nutrition (NIN), Indian Council of Medical Research. India Rural Survey of Diet and Nutritional Status Data 2000-2001. [Data shared for this analysis]

National Nutrition Monitoring Bureau, National Institute of Nutrition (NIN), Indian Council of Medical Research. India Rural Survey of Diet and Nutritional Status Data 2004-2006. [Data shared for this analysis]

National Nutrition Monitoring Bureau, National Institute of Nutrition (NIN), Indian Council of Medical Research. India Rural Survey of Diet and Nutritional Status Report 1994-1995. Hyderabad, India: NIN.

National Nutrition Monitoring Bureau, National Institute of Nutrition (NIN), Indian Council of Medical Research. India Rural Survey of Diet and Nutritional Status Report 2000-2001. Hyderabad, India: NIN.

National Nutrition Monitoring Bureau, National Institute of Nutrition (NIN), Indian Council of Medical Research. India Rural Survey of Diet and Nutritional Status Report 2004-2006. Hyderabad, India: NIN.

National Nutrition Monitoring Bureau, National Institute of Nutrition (NIN), Indian Council of Medical Research. India Rural Third Repeat Survey of Diet and Nutritional Status Data 2011-2012. [Data shared for this analysis]

National Nutrition Monitoring Bureau, National Institute of Nutrition (NIN), Indian Council of Medical Research. India Rural Third Repeat Survey of Diet and Nutritional Status Report 2011-2012. Hyderabad, India: NIN.

National Nutrition Monitoring Bureau, National Institute of Nutrition (NIN), Indian Council of Medical Research. India Survey of Diet and Nutritional Status Data 1990-1992. [Data shared for this analysis]

National Nutrition Monitoring Bureau, National Institute of Nutrition (NIN), Indian Council of Medical Research. India Survey of Diet and Nutritional Status Data 1991-1992. [Data shared for this analysis]

National Nutrition Monitoring Bureau, National Institute of Nutrition (NIN), Indian Council of Medical Research. India Survey of Diet and Nutritional Status Report 1990-1992. Hyderabad, India: NIN.

National Nutrition Monitoring Bureau, National Institute of Nutrition (NIN), Indian Council of Medical Research. India Survey of Diet and Nutritional Status Report 1991-1992. Hyderabad, India: NIN.

National Nutrition Monitoring Bureau, National Institute of Nutrition (NIN), Indian Council of Medical Research. India Tribal First Repeat Survey of Diet and Nutritional Status Data 1998-1999. [Data shared for this analysis]

National Nutrition Monitoring Bureau, National Institute of Nutrition (NIN), Indian Council of Medical Research. India Tribal First Repeat Survey of Diet and Nutritional Status Report 1998-1999. Hyderabad, India: NIN.

National Nutrition Monitoring Bureau, National Institute of Nutrition (NIN), Indian Council of Medical Research. India Tribal Second Repeat Survey of Diet and Nutritional Status Data 2007-2008. [Data shared for this analysis]

National Nutrition Monitoring Bureau, National Institute of Nutrition (NIN), Indian Council of Medical Research. India Tribal Second Repeat Survey of Diet and Nutritional Status Report 2007-2008. Hyderabad, India: NIN.
National Nutrition Monitoring Bureau, National Institute of Nutrition (NIN), Indian Council of Medical Research. India Urban Slums Survey of Diet and Nutritional Status Data 1993-1994. [Data shared for this analysis]
National Nutrition Monitoring Bureau, National Institute of Nutrition (NIN), Indian Council of Medical Research. India Urban Slums Survey of Diet and Nutritional Status Report 1993-1994. Hyderabad, India: NIN.
National Nutrition Monitoring Bureau, National Institute of Nutrition, Indian Council of Medical Research. India National Nutrition Monitoring Bureau Eight States Pooled Data 1991-1992. [Data shared for this analysis]
Nazareth Hospital. Inpatient and Discharge Data 2014. Shillong, Meghalaya: Nazareth Hospital. [Data shared for this analysis]
Office of the Registrar General & Census Commissioner, Ministry of Home Affairs, Government of India. India Annual Health Survey Data 2010-2011. New Delhi, India: Office of the Registrar General & Census Commissioner.
Office of the Registrar General & Census Commissioner, Ministry of Home Affairs, Government of India. India Annual Health Survey Data 2010-2013. New Delhi, India: Office of the Registrar General & Census Commissioner.
Office of the Registrar General & Census Commissioner, Ministry of Home Affairs, Government of India. India Annual Health Survey Data 2011-2012. New Delhi, India: Office of the Registrar General & Census Commissioner.
Office of the Registrar General & Census Commissioner, Ministry of Home Affairs, Government of India. India Annual Health Survey Data 2012-2013. New Delhi, India: Office of the Registrar General & Census Commissioner.
Office of the Registrar General & Census Commissioner, Ministry of Home Affairs, Government of India. India Annual Health Survey Report 2010-2011. New Delhi, India: Office of the Registrar General & Census Commissioner.
Office of the Registrar General & Census Commissioner, Ministry of Home Affairs, Government of India. India Annual Health Survey Report 2010-2013. New Delhi, India: Office of the Registrar General & Census Commissioner.
Office of the Registrar General & Census Commissioner, Ministry of Home Affairs, Government of India. India Annual Health Survey Report 2011-2012. New Delhi, India: Office of the Registrar General & Census Commissioner.
Office of the Registrar General & Census Commissioner, Ministry of Home Affairs, Government of India. India Annual Health Survey Report 2012-2013. New Delhi, India: Office of the Registrar General & Census Commissioner.
Office of the Registrar General & Census Commissioner, Ministry of Home Affairs, Government of India. India Census 1981. New Delhi, India: Office of the Registrar General & Census Commissioner.
Office of the Registrar General & Census Commissioner, Ministry of Home Affairs, Government of India. India Medical Certification of Cause of Death Report 2005. New Delhi, India: Office of the Registrar General & Census Commissioner.
Office of the Registrar General & Census Commissioner, Ministry of Home Affairs, Government of India. India Medical Certification of Cause of Death Report 2006. New Delhi, India: Office of the Registrar General & Census Commissioner.
Office of the Registrar General & Census Commissioner, Ministry of Home Affairs, Government of India. India Medical Certification of Cause of Death Report 2008. New Delhi, India: Office of the Registrar General & Census Commissioner.
Office of the Registrar General & Census Commissioner, Ministry of Home Affairs, Government of India. India Medical Certification of Cause of Death Report 2009. New Delhi, India: Office of the Registrar General & Census Commissioner; 2014.
Office of the Registrar General & Census Commissioner, Ministry of Home Affairs, Government of India. India Medical Certification of Cause of Death Report 2010. New Delhi, India: Office of the Registrar General & Census Commissioner; 2014.
Office of the Registrar General & Census Commissioner, Ministry of Home Affairs, Government of India. India Medical Certification of Cause of Death State-Level Tabulations 2000. New Delhi, India: Office of the Registrar General & Census Commissioner.
Office of the Registrar General & Census Commissioner, Ministry of Home Affairs, Government of India. India Medical Certification of Cause of Death State-Level Tabulations 2001. New Delhi, India: Office of the Registrar General & Census Commissioner.
Office of the Registrar General & Census Commissioner, Ministry of Home Affairs, Government of India. India Medical Certification of Cause of Death State-Level Tabulations 2002. New Delhi, India: Office of the Registrar General & Census Commissioner.
Office of the Registrar General & Census Commissioner, Ministry of Home Affairs, Government of India. India Medical Certification of Cause of Death State-Level Tabulations 2003. New Delhi, India: Office of the Registrar General & Census Commissioner.
Office of the Registrar General & Census Commissioner, Ministry of Home Affairs, Government of India. India Medical Certification of Cause of Death State-Level Tabulations 2004. New Delhi, India: Office of the Registrar General & Census Commissioner.

Office of the Registrar General & Census Commissioner, Ministry of Home Affairs, Government of India. India Population and Housing Census 2001. New Delhi, India: Office of the Registrar General & Census Commissioner.
Office of the Registrar General & Census Commissioner, Ministry of Home Affairs, Government of India. India Population and Housing Census 2011. New Delhi, India: Office of the Registrar General & Census Commissioner; 2012.
Office of the Registrar General & Census Commissioner, Ministry of Home Affairs, Government of India. India SRS Statistical Report 1999. New Delhi, India: Office of the Registrar General & Census Commissioner.
Office of the Registrar General & Census Commissioner, Ministry of Home Affairs, Government of India. India SRS Statistical Report 2000. New Delhi, India: Office of the Registrar General & Census Commissioner.
Office of the Registrar General & Census Commissioner, Ministry of Home Affairs, Government of India. India SRS Statistical Report 2001. New Delhi, India: Office of the Registrar General & Census Commissioner; 2005.
Office of the Registrar General & Census Commissioner, Ministry of Home Affairs, Government of India. India SRS Statistical Report 2002. New Delhi, India: Office of the Registrar General & Census Commissioner; 2005.
Office of the Registrar General & Census Commissioner, Ministry of Home Affairs, Government of India. India SRS Statistical Report 2003. New Delhi, India: Office of the Registrar General and Census Commissioner; 2005.
Office of the Registrar General & Census Commissioner, Ministry of Home Affairs, Government of India. India SRS Statistical Report 2004. New Delhi, India: Office of the Registrar General and Census Commissioner; 2006.
Office of the Registrar General & Census Commissioner, Ministry of Home Affairs, Government of India. India SRS Statistical Report 2005. New Delhi, India: Office of the Registrar General and Census Commissioner; 2006.
Office of the Registrar General & Census Commissioner, Ministry of Home Affairs, Government of India. India SRS Statistical Report 2006. New Delhi, India: Office of the Registrar General and Census Commissioner; 2008.
Office of the Registrar General & Census Commissioner, Ministry of Home Affairs, Government of India. India SRS Statistical Report 2007. New Delhi, India: Office of the Registrar General and Census Commissioner; 2008.
Office of the Registrar General & Census Commissioner, Ministry of Home Affairs, Government of India. India SRS Statistical Report 2008. New Delhi, India: Office of the Registrar General and Census Commissioner; 2010.
Office of the Registrar General & Census Commissioner, Ministry of Home Affairs, Government of India. India SRS Statistical Report 2009. New Delhi, India: Office of the Registrar General and Census Commissioner.
Office of the Registrar General & Census Commissioner, Ministry of Home Affairs, Government of India. India SRS Statistical Report 2010. New Delhi, India: Office of the Registrar General and Census Commissioner; 2012.
Office of the Registrar General & Census Commissioner, Ministry of Home Affairs, Government of India. India SRS Statistical Report 2011. New Delhi, India: Office of the Registrar General and Census Commissioner; 2013.
Office of the Registrar General & Census Commissioner, Ministry of Home Affairs, Government of India. India SRS Statistical Report 2012. New Delhi, India: Office of the Registrar General and Census Commissioner; 2013.
Office of the Registrar General & Census Commissioner, Ministry of Home Affairs, Government of India. India SRS Statistical Report 2013. New Delhi, India: Office of the Registrar General and Census Commissioner; 2014.
Office of the Registrar General & Census Commissioner, Ministry of Home Affairs, Government of India. India SRS Statistical Report 2014. New Delhi, India: Office of the Registrar General and Census Commissioner.
Office of the Registrar General & Census Commissioner, Ministry of Home Affairs, Government of India. India SRS Verbal Autopsy 2004-2006. New Delhi, India: Office of the Registrar General and Census Commissioner. [Data shared for this analysis]
Office of the Registrar General & Census Commissioner, Ministry of Home Affairs, Government of India. India SRS Verbal Autopsy 2007-2009. New Delhi, India: Office of the Registrar General and Census Commissioner. [Data shared for this analysis]
Office of the Registrar General & Census Commissioner, Ministry of Home Affairs, Government of India. India SRS Verbal Autopsy 2010-2013. New Delhi, India: Office of the Registrar General and Census Commissioner. [Data shared for this analysis]
Office of the Registrar General & Census Commissioner, Ministry of Home Affairs, Government of India. India SRS Verbal Autopsy Report 2004-2006. New Delhi, India: Office of the Registrar General and Census Commissioner.
Office of the Registrar General & Census Commissioner, Ministry of Home Affairs, Government of India. India SRS Verbal Autopsy Report 2007-2009. New Delhi, India: Office of the Registrar General and Census Commissioner.
Office of the Registrar General & Census Commissioner, Ministry of Home Affairs, Government of India. India SRS Verbal Autopsy Report 2010-2013. New Delhi, India: Office of the Registrar General and Census Commissioner.

Office of the Registrar General and Census Commissioner, Ministry of Health and Family Welfare, Government of India, National Institute of Health and Family Welfare, Nutrition Foundation of India, National Institute of Nutrition, Indian Council of Medical Research. India Clinical, Anthropometric and Bio-chemical (CAB) Survey Data 2014 [Biomarker Component of Annual Health Survey]. New Delhi, India: Office of the Registrar General and Census Commissioner.
Office of the Registrar General and Census Commissioner, Ministry of Health and Family Welfare, Government of India, National Institute of Health and Family Welfare, Nutrition Foundation of India, National Institute of Nutrition, Indian Council of Medical Research. India Clinical, Anthropometric and Bio-chemical (CAB) Survey Report 2014 [Biomarker Component of Annual Health Survey]. New Delhi, India: Office of the Registrar General and Census Commissioner.
Olofin I, McDonald CM, Ezzati M, Flaxman S, Black RE, Fawzi WW, Caulfield LE, Danaei G; Nutrition Impact Model Study (anthropometry cohort pooling). Associations of suboptimal growth with all-cause and cause-specific mortality in children under five years: a pooled analysis of ten prospective studies. PLoS One. 2013; 8:e64636.
Panigrahi S, Patra PK, Khodiar PK. Neonatal screening of sickle cell anemia: a preliminary report. Indian J Pediatr. 2012; 79:747–50.
Pasricha S-R, Black J, Muthayya S, Shet A, Bhat V, Nagaraj S, Prashanth NS, Sudarshan H, Biggs B-A, Shet AS. Determinants of anemia among young children in rural India. Pediatrics. 2010; 126:e140-9.
Pathak P, Singh P, Kapil U, Raghuvanshi RS. Prevalence of iron, vitamin A, and iodine deficiencies amongst adolescent pregnant mothers. Indian J Pediatr. 2003; 70:299-301.
Pathak P, Tandon M, Kapil U, Singh C. Prevalence of iron deficiency anemia amongst pregnant women in urban slum communities of Delhi. Indian Pediatr. 1999; 36:322-3.
Prasad KN, Rao RS, Sujatha A. Birth weight pattern in Karnataka. Indian Pediatr. 1994; 31:836-9.
Prinja S, Thakur JS, Bhatia SS. Pilot testing of WHO child growth standards in Chandigarh: implications for India's child health programmes. Bull World Health Organ. 2009; 87:116-22.
Purwar M, Ughade S, Bhagat B, Agarwal V, Kulkarni H. Bacterial vaginosis in early pregnancy and adverse pregnancy outcome. J Obstet Gynaecol Res. 2001; 27:175-81.
Rahmathullah L, Tielsch JM, Thulasiraj RD, Katz J, Coles C, Devi S, John R, Prakash K, Sadanand AV, Edwin N, Kamaraj C. Impact of supplementing newborn infants with vitamin A on early infant mortality: community based randomised trial in southern India. BMJ. 2003; 327:254.
Rahmathullah L, Underwood BA, Thulasiraj RD, Milton RC, Ramaswamy K, Rahmathullah R, Babu G. Reduced mortality among children in southern India receiving a small weekly dose of vitamin A. N Engl J Med. 1990; 323:929-35. as it appears in Imdad A, Herzer K, Mayo-Wilson E, Yakoob MY, Bhutta ZA. Vitamin A supplementation for preventing morbidity and mortality in children from 6 months to 5 years of age. Cochrane Database Syst Rev. 2010; CD008524.
Rajaratnam J, Abel R, Asokan JS, Jonathan P. Prevalence of anemia among adolescent girls of rural Tamilnadu. Indian Pediatr. 2000; 37:532-6.
Rajaratnam J, Abel R, Ganesan C, Jayaseelan SA. Maternal anaemia: a persistent problem in rural Tamil Nadu. Natl Med J India. 2000; 13:242-5.
Ramakrishnan U, Latham MC, Abel R, Frongillo EA Jr. Vitamin A supplementation and morbidity among preschool children in south India. Am J Clin Nutr. 1995; 61:1295-303.
Raman L, Subbalaxmi P, Vasumathi N, Rawal A, Vasanthi G, Parvathi C, Adinarayana K, Pawashe A, Rao K. Iron and folic acid nutritional status of women in slum. Nutr Report Int. 1989; 73-81.
Randomised trial to assess benefits and safety of vitamin A supplementation linked to immunisation in early infancy. WHO/CHD Immunisation-Linked Vitamin A Supplementation Study Group.Lancet. 1998; 352: 1257-63.
Rao KV, Radhaiah G, Raju SV. Association of growth status and the prevalence of anaemia in preschool children. Indian J Med Res. 1980;71: 237-46.
Rawat CM, Garg SK, Singh JV, Bhatnagar M, Chopra H, Bajpai SK. Prevalence of anaemia among adolescent girls in rural area of District Meerut, U.P. Indian J Public Health. 2001; 45:24-6.
Ray SK, Mallick S, Kumar S, Biswas B. A rapid assessment of anaemia in pregnancy in West Bengal with special reference to care seeking behaviour of mothers. Indian J Public Health. 2000; 44:58-64.
Reddaiah VP, Kapoor SK. Socio-biological factors in underfive deaths in a rural area. Indian J Pediatr. 1992; 59 567-71.
Reddaiah VP, Raj PP, Ramachandran K, Nath LM, Sood SK, Madan N, Rusia U. Supplementary iron dose in pregnancy anemia prophylaxis. Indian J Pediatr. 1989; 56:109-14.
Rusia U, Gupta S, Agarwal N, Singh K, Sikka M, Madan N. Efficacy of the new program of iron supplementation in pregnancy in India. Indian J Hematol Blood Transfus. 1999; 17:87-91.

Rusia U, Madan N, Agarwal N, Sikka M, Sood SK. Effect of maternal iron deficiency anaemia on foetal outcome. Indian J Pathol Microbiol. 1995; 38:273-9.

Sachdev HPS, Osmond C, Fall CHD, Lakshmy R, Ramji S, Dey Biswas SK, Prabhakaran D, Tandon N, Reddy KS, Barker DJP, Bhargava SK. Predicting adult metabolic syndrome from childhood body mass index: follow-up of the New Delhi birth cohort. Arch Dis Child. 2009; 94:768-74.

Sachdeva S, Alam S, Beig FK, Khan Z, Khalique N. Determinants of vitamin A deficiency amongst children in Aligarh District, Uttar Pradesh. Indian Pediatr. 2011; 48:861-6.

Saleem S, McClure EM, Goudar SS, Patel A, Esamai F, Garces A, Chomba E, Althabe F, Moore J, Kodkany B, Pasha O, Belizan J, Mayansyan A, Derman RJ, Hibberd PL, Liechty EA, Krebs NF, Hambidge KM, Buekens P, Carlo WA, Wright LL, Koso-Thomas M, Jobe AH, Goldenberg RL, on behalf of the Global Network Maternal Newborn Health Registry Study Investigators. A prospective study of maternal, fetal and neonatal deaths in low- and middle-income countries. Bull World Health Organ. 2014; 92:605-12.

Sarin A. Severe anemia of pregnancy, recent experience. Int J Gynaecol Obstet. 1995; 50(Suppl 2): 45-49.

Saxena N, Nayar D, Kapil U. Prevalence of underweight, stunting and wasting. Indian Pediatr. 1997; 34:627-31.

Scott W, Mathew NT. A Development Monitoring Service at the Local Level: Monitoring Change in Kerala: The First Five Years. Geneva, Switzerland: United Nations Research Institute for Social Development; 1985.

Sehgal A, Telang S, Passah SM, Jyothi MC. Maternal and Neonatal Profile and Immediate Outcome in Extremely Low Birth Weight Babies in Delhi. Trop Doct. 2004; 34:165-8.

Sethi V, Goindi G, Kapil U. Prevalence of anemia amongst primary school age children (6-11 years) in National Capital Territory of Delhi. Indian J Pediatr. 2003; 70:519-20.

Shah SN, Bakash A, Rauf A, Muzzafar A, Zuthshi ML. Incidence of iron deficiency anaemia in rural population of Kashmir. Indian J Public Health. 1982; 26:144-54.

Sharma A, Prasad K, Rao KV. Identification of an appropriate strategy to control anemia in adolescent girls of poor communities. Indian Pediatr. 2000; 37:261-7.

Sharma JB, Soni D, Murthy NS, Malhotra M. Effect of dietary habits on prevalence of anemia in pregnant women of Delhi. J Obstet Gynaecol Res. 2003; 29:73-8.

Shobha S, Sharada D. Efficacy of twice weekly iron supplementation in anemic adolescent girls. Indian Pediatr. 2003; 40:1186-90.

Sidhu S, Kumari K, Uppal M. Prevalence of anemia in Schedule Caste preschool children of Punjab. Indian J Med Sci. 2002; 56:218-21.

Sidhu S. Incidence of anaemia among scheduled caste pre-school children of Punjab. Indian J Matern Child Health. 1996; 7:76-7.

Singhal PK, Mathur GP, Mathur S, Singh YD. Mortality patterns in under six children in I.C.D.S. urban slum. Indian Pediatr. 1986; 23: 617-22.

Singla PN, Agarwal KN. Studies on normal hemoglobin and hematocrit values in healthy children based on hematinic supplementation. Indian Pediatr. 1981; 18:821-5.

Sivakumar B, Brahmam GN, Madhavan Nair K, Ranganathan S, Vishnuvardhan Rao M, Vijayaraghavan K, Krishnaswamy K. Prospects of fortification of salt with iron and iodine. Br J Nutr. 2001; 167-173.

Smith JL, Brooker S. Impact of hookworm infection and deworming on anaemia in non-pregnant populations: a systematic review. Trop Med Int Health. 2010; 15:776–95.

Sood SK, Ramachandran K, Rani K, Ramalingaswami V, Mathan VI, Ponniah J, Baker SJ. WHO sponsored collaborative studies on nutritional anaemia in India. The effect of parenteral iron administration in the control of anaemia of pregnancy. Br J Nutr. 1979; 42: 399-406.

Spector JM, Agrawal P, Kodkany B, Lipsitz S, Lashoher A, Dziekan G, Bahl R, Merialdi M, Mathai M, Lemer C, Gawande A. Improving Quality of Care for Maternal and Newborn Health: Prospective Pilot Study of the WHO Safe Childbirth Checklist Program. PLoS One. 2012; 7:e35151.

State Bureau of Health Intelligence & Vital Statistics, Directorate of Health Services, Department of Health & Family Welfare, Government of Odisha. India - Odisha Medical Certification of Cause of Death Data 2009. Odisha, India: Department of Health & Family Welfare, Government of Odisha.

State Bureau of Health Intelligence & Vital Statistics, Directorate of Health Services, Department of Health & Family Welfare, Government of Odisha. India - Odisha Medical Certification of Cause of Death Data 2010. Odisha, India: Department of Health & Family Welfare, Government of Odisha

State Bureau of Health Intelligence & Vital Statistics, Directorate of Health Services, Department of Health & Family Welfare, Government of Odisha. India - Odisha Medical Certification of Cause of Death Data 2011. Odisha, India: Department of Health & Family Welfare, Government of Odisha.

State Bureau of Health Intelligence & Vital Statistics, Directorate of Health Services, Department of Health & Family Welfare, Government of Odisha. India - Odisha Medical Certification of Cause of Death Data 2012. Odisha, India: Department of Health & Family Welfare, Government of Odisha.

State Bureau of Health Intelligence & Vital Statistics, Directorate of Health Services, Department of Health & Family Welfare, Government of Odisha. India - Odisha Medical Certification of Cause of Death Data 2013. Odisha, India: Department of Health & Family Welfare, Government of Odisha.

Straughn HK, Goldenberg RL, Tolosa JE, Daly S, de Codes J, Festin MR, Limpongsanurak S, Lumbiganon P, Paul VK, Peedicayil A, Purwar M, Sabogal JC, Shenoy S. Birthweight-specific neonatal mortality in developing countries and obstetric practices. Int J Gynaecol Obstet. 2003; 80:71-8.

Swain S, Singh S, Bhatia BD, Pandey S, Krishna M. Maternal hemoglobin and serum albumin and fetal growth. Indian Pediatr. 1994; 31: 777-82.

Swaminathan M, Apte S, Someswara Rao K. Nutrition of the people of Ankola Taluk N. Kanara. Indian J Med Res. 1960; 762-74.

Talsania NJ, Lala MK. Evaluation of antenatal risk scoring in a preterm birth prevention and perinatal loss. Indian J Matern Child Health. 1994; 5:5-9.

Thangaleela T, Vijayalakshmi P. Prevalence of Anaemia in Pregnancy. Indian J Nutr Diet.1994 ; 31:26–9.

Thilothammal N, Sujaritha R, Banu K, Ratnam S, Ezhlarasi. The nutritional status of south Indian women in child bearing age and its influence on birth weight and maturity of offspring. J Obstet Gynaecol India. 1993; 871-7.

Thomas SV, Sindhu K, Ajaykumar B, Sulekha Devi PB, Sujamol J. Maternal and obstetric outcome of women with epilepsy. Seizure. 2009; 18:163-6.

Tielsch JM, Rahmathullah L, Thulasiraj RD, Katz J, Coles C, Sheeladevi S, John R, Prakash K. Newborn vitamin A dosing reduces the case fatality but not incidence of common childhood morbidities in South India. J Nutr. 2007; 137:2470-4.

Trivedi SS, Pasrija S. Teenage pregnancies and their obstetric outcomes. Trop Doct. 2007; 37:85-8.

Uberoi IS, DeSweemer C, Taylor CE. A study of anemia among rural Punjabi children. Indian J Med Res. 1972; 60:793-9.

Upadhyay RP, Rai SK, Krishnan A. Using three delays model to understand the social factors responsible for neonatal deaths in rural Haryana, India. J Trop Pediatr. 2013; 59:100-5.

Venkatarao T, Ramakrishnan R, Nair NG, Radhakrishnan S, Sundaramoorthy L, Koya PK, Kumar SK. Effect of vitamin A supplementation to mother and infant on morbidity in infancy. Indian Pediatr. 1996; 33:279-86. as it appears in Imdad A, Herzer K, Mayo-Wilson E, Yakoob MY, Bhutta ZA. Vitamin A supplementation for preventing morbidity and mortality in children from 6 months to 5 years of age. Cochrane Database Syst Rev. 2010; CD008524.

Verma M, Chhatwal J, Kaur G. Prevalence of anemia among urban school children of Punjab. Indian Pediatr. 1998; 35:1181-6.

Vijayaraghavan K, Rao DH. Diet & nutrition situation in rural India. Indian J Med Res. 1998; 108:243-53.

Vogel JP, Souza JP, Mori R, Morisaki N, Lumbiganon P, Laopaiboon M, Ortiz-Panozo E, Hernandez B, Pérez-Cuevas R, Roy M, Mittal S, Cecatti JG, Tunçalp Ö, Gülmezoglu AM; WHO Multicountry Survey on Maternal and Newborn Health Research Network. Maternal complications and perinatal mortality: findings of the World Health Organization Multicountry Survey on Maternal and Newborn Health. BJOG. 2014; 121(Supp 1): 76-88.

Working Group on Fortification of Salt with Iron. Use of common salt fortified with iron in the control and prevention of anemia--a collaborative study. Report of the Working Group on Fortification of Salt with Iron. Am J Clin Nutr. 1982; 35:1442-51.

World Bank. Survey of Living Conditions 1997-1998, Uttar Pradesh and Bihar. Washington D.C., United States: World Bank.

World Bank. World Development Indicators - Vitamin A Supplementation Coverage Rate. Washington D.C., United States: World Bank.

World Health Organization (WHO), United Nations Children's Fund (UNICEF). WHO and UNICEF Reported Administrative Data for Immunization Coverage Time Series. Available from: http://www.who.int/immunization/monitoring_surveillance/data/en/

World Health Organization (WHO). Global Database on Child Growth and Malnutrition - Historical. Geneva, Switzerland: WHO.

World Health Organization (WHO). WHO Global Database on Anemia, Nutrition Landscape Information System. Geneva, Switzerland: WHO.

World Health Organization (WHO). WHO Global Database on Child Growth and Malnutrition. Geneva, Switzerland: WHO.

World Health Organization (WHO). WHO Global Database on Vitamin A Deficiency. Geneva, Switzerland: WHO.

World Health Organization (WHO). WHO Global Survey on Maternal and Perinatal Health 2004-2008. Geneva, Switzerland: WHO.

World Health Organization. Study on Global Ageing and Adult Health (SAGE) Pilot Study 2005 Data from the Data Archive of Social Research on Aging. Los Altos, United States: Sociometrics Corporation. Available from: http://home.socio.com/age2728.php

Yusufji, Mathan VI, Baker SJ. Iron, folate, and vitamin B 12 nutrition in pregnancy: a study of 1000 women from southern India. Bull World Health Organ. 1973; 48:15-22.

3. Grouping of the states of India based on Socio-demographic Index, 2017

State group (population in 2017)	States of India*	SDI in 2017
**Low SDI states (675 million)**	Bihar	0.43
	Madhya Pradesh	0.49
	Jharkhand	0.49
	Uttar Pradesh	0.49
	Rajasthan	0.49
	Chhattisgarh	0.51
	Odisha	0.52
	Assam	0.53
**Middle SDI states (387 million)**	Andhra Pradesh	0.54
	West Bengal	0.54
	Tripura	0.54
	Arunachal Pradesh	0.56
	Meghalaya	0.56
	Karnataka	0.57
	Telangana	0.58
	Gujarat	0.58
	Manipur	0.59
	Jammu and Kashmir†	0.59
	Haryana	0.60
**High SDI states (318 million)**	Uttarakhand	0.61
	Tamil Nadu	0.62
	Mizoram	0.62
	Maharashtra	0.62
	Punjab	0.62
	Sikkim	0.63
	Nagaland	0.63
	Himachal Pradesh	0.63
	Union territories other than Delhi	0.65
	Kerala	0.66
	Delhi	0.72
	Goa	0.74

SDI=Socio-demographic Index
SDI as computed by GBD in 2017 as described elsewhere (Lancet 2018; 392: 1995-2051).
*The states are listed in increasing order of Socio-demographic Index in 2017.
†The state of Jammu and Kashmir was divided into two union territories in August 2019; as we are reporting findings up to 2017, we report findings for the state of Jammu and Kashmir.

4. Death and DALY rates attributable to child and maternal malnutrition in under-five children in the states of India, 2017

States*	Death rate per 100,000 in under-5 attributable to malnutrition (95% uncertainty interval)	Percentage of total deaths in under-5 attributable to malnutrition (95% uncertainty interval)	Ranking of malnutrition among all risk factors for under-5	DALY rate per 100,000 in under-5 attributable to malnutrition (95% uncertainty interval)	Percentage of total DALYs in under-5 attributable to malnutrition (95% uncertainty interval)	Ranking of malnutrition among all risk factors for under-5
**India**	546 (510 to 587)	68.2 (65.8 to 70.7)	1	50,627 (47,301 to 54,199)	67.1 (64.9 to 69.4)	1
**Low SDI**	**706 (648 to 768)**	**69.2 (63.5 to 75.4)**	**1**	**64,615 (59,490 to 70,209)**	**68.3 (65.8 to 70.9)**	**1**
Bihar	730 (622 to 873)	72.7 (69.0 to 76.0)	1	66,673 (57,080 to 79,222)	71.5 (68.1 to 74.6)	1
Madhya Pradesh	577 (493 to 681)	67.8 (64.1 to 71.0)	1	53,699 (46,059 to 62,838)	66.9 (63.5 to 69.9)	1
Jharkhand	472 (397 to 567)	67.7 (62.7 to 72.2)	1	44,597 (38,220 to 52,817)	66.6 (62.2 to 70.6)	1
Uttar Pradesh	821 (697 to 968)	68.6 (64.7 to 72.2)	1	74,782 (63,945 to 87,383)	67.8 (64.1 to 71.1)	1
Rajasthan	669 (572 to 782)	72.2 (69.2 to 75.0)	1	61,574 (53,244 to 71,713)	71.1 (68.2 to 73.8)	1
Chhattisgarh	590 (504 to 696)	71.6 (67.0 to 75.3)	1	53,956 (46,325 to 63,247)	70.2 (65.9 to 73.7)	1
Odisha	588 (491 to 711)	62.1 (57.2 to 67.0)	1	53,850 (45,498 to 64,541)	61.5 (56.8 to 66.2)	1
Assam	705 (589 to 856)	65.1 (61.2 to 68.6)	1	63,493 (53,226 to 76,591)	64.2 (60.4 to 67.7)	1
**Middle SDI**	**383 (350 to 417)**	**66.0 (60.5 to 72.0)**	**1**	**36,374 (33,490 to 39,393)**	**64.9 (62.6 to 67.1)**	**1**
Andhra Pradesh	360 (273 to 478)	67.5 (64.1 to 70.5)	1	34,493 (26,737 to 44,824)	66.0 (62.9 to 68.8)	1
West Bengal	385 (324 to 465)	67.2 (63.7 to 70.3)	1	36,107 (30,547 to 43,198)	65.6 (62.6 to 68.6)	1
Tripura	605 (490 to 756)	67.6 (63.8 to 71.0)	1	55,403 (45,300 to 68,753)	66.6 (63.0 to 69.8)	1
Arunachal Pradesh	315 (255 to 383)	65.7 (60.2 to 70.6)	1	30,293 (25,248 to 36,253)	64.2 (59.6 to 68.6)	1
Meghalaya	495 (411 to 597)	59.1 (53.1 to 65.7)	1	45,392 (37,953 to 54,088)	58.4 (52.9 to 64.7)	1
Karnataka	336 (287 to 394)	63.1 (59.8 to 66.0)	1	32,315 (27,822 to 37,466)	62.2 (59.3 to 64.8)	1
Telangana	307 (234 to 408)	67.9 (64.4 to 71.1)	1	30,197 (23,538 to 39,180)	66.4 (63.1 to 69.2)	1
Gujarat	430 (367 to 502)	66.2 (62.8 to 69.4)	1	40,913 (35,374 to 47,709)	65.4 (62.4 to 68.4)	1
Manipur	374 (305 to 463)	61.8 (56.8 to 66.1)	1	34,126 (27,902 to 41,901)	60.2 (55.7 to 64.3)	1
Jammu and Kashmir†	425 (360 to 506)	66.2 (63.2 to 68.8)	1	39,653 (33,873 to 46,800)	65.0 (62.2 to 67.4)	1
Haryana	427 (365 to 497)	65.4 (62.3 to 68.3)	1	40,608 (34,969 to 46,810)	64.5 (61.6 to 67.2)	1
**High SDI**	**272 (249 to 299)**	**64.2 (58.9 to 70.7)**	**1**	**26,495 (24,489 to 28,984)**	**63.0 (60.5 to 65.4)**	**1**
Uttarakhand	351 (295 to 416)	64.4 (60.4 to 68.2)	1	33,570 (28,513 to 39,332)	63.4 (59.7 to 66.8)	1
Tamil Nadu	168 (143 to 198)	59.8 (56.1 to 63.0)	1	17,275 (14,876 to 20,013)	58.6 (55.6 to 61.5)	1
Mizoram	481 (397 to 581)	60.8 (56.4 to 65.3)	1	43,715 (36,406 to 52,457)	59.8 (55.8 to 64.1)	1
Maharashtra	307 (260 to 363)	66.9 (63.1 to 70.5)	1	29,742 (25,415 to 34,710)	65.7 (62.3 to 68.9)	1
Punjab	382 (326 to 450)	63.5 (59.8 to 66.9)	1	35,894 (30,999 to 41,709)	62.5 (59.1 to 65.5)	1
Sikkim	237 (188 to 304)	62.7 (57.9 to 67.6)	1	22,732 (18,378 to 28,648)	60.8 (56.5 to 65.0)	1
Nagaland	593 (467 to 757)	61.8 (56.5 to 66.5)	1	53,572 (42,526 to 67,873)	60.9 (55.9 to 65.4)	1
Himachal Pradesh	361 (295 to 443)	64.8 (61.3 to 68.0)	1	34,226 (28,423 to 41,319)	63.8 (60.6 to 66.7)	1
Union Territories other than Delhi	414 (339 to 507)	63.2 (59.6 to 66.8)	1	39,095 (32,524 to 47,363)	62.6 (59.3 to 66.0)	1
Kerala	100 (83 to 121)	50.8 (46.0 to 56.6)	1	11,002 (9,436 to 12,935)	50.7 (46.5 to 55.3)	1
Delhi	419 (346 to 507)	68.5 (63.5 to 72.5)	1	40,029 (33,503 to 48,064)	67.4 (63.0 to 71.2)	1
Goa	267 (191 to 370)	60.8 (56.1 to 64.9)	1	25,764 (19,100 to 34,841)	60.0 (55.8 to 63.8)	1

DALY=disability-adjusted life-year. SDI=Socio-demographic Index.
*The states are listed in increasing order of Socio-demographic Index in 2017.
†The state of Jammu and Kashmir was divided into two union territories in August 2019; as we are reporting findings up to 2017, we report findings for the state of Jammu and Kashmir.

5. DALY rates attributable to child and maternal malnutrition in all age groups combined together in the states of India, 2017

States*	DALY rate per 100,000 attributable to malnutrition (95% uncertainty interval)	Percentage of total DALYs attributable to malnutrition (95% uncertainty interval)	Ranking of malnutrition among all risk factors
**India**	6,011 (5,478 to 6,608)	17.3 (16.3 to 18.2)	1
**Low SDI**	**8,393 (7,672 to 9,170)**	**22.4 (20.9 to 23.9)**	**1**
Bihar	9,113 (7,957 to 10,595)	26.4 (23.9 to 29.4)	1
Madhya Pradesh	7,318 (6,345 to 8,465)	19.8 (18.0 to 22.0)	1
Jharkhand	6,161 (5,293 to 7,161)	18.8 (17.0 to 20.9)	1
Uttar Pradesh	9,707 (8,397 to 11,216)	23.9 (21.5 to 26.6)	1
Rajasthan	7,976 (6,949 to 9,192)	22.7 (20.5 to 25.4)	1
Chhattisgarh	6,902 (6,031 to 7,986)	16.7 (14.9 to 18.7)	1
Odisha	5,935 (5,058 to 7,003)	15.9 (14.0 to 18.2)	1
Assam	7,737 (6,645 to 9,133)	20.7 (18.2 to 23.4)	1
**Middle SDI**	**4,203 (3,728 to 4,760)**	**12.9 (12.1 to 13.7)**	**1**
Andhra Pradesh	3,808 (3,071 to 4,742)	11.3 (10.4 to 12.3)	2
West Bengal	3,890 (3,333 to 4,572)	12.5 (11.1 to 14.1)	2
Tripura	5,567 (4,641 to 6,780)	16.3 (15.0 to 17.7)	1
Arunachal Pradesh	4,347 (3,674 to 5,202)	16.8 (15.7 to 18.2)	1
Meghalaya	6,242 (5,238 to 7,384)	20.6 (19.1 to 22.4)	1
Karnataka	3,672 (3,142 to 4,250)	10.0 (8.9 to 11.1)	3
Telangana	3,728 (3,017 to 4,603)	12.4 (11.4 to 13.5)	1
Gujarat	5,109 (4,350 to 5,945)	15.6 (14.1 to 17.3)	1
Manipur	3,731 (3,136 to 4,485)	12.3 (11.1 to 13.5)	1
Jammu and Kashmir†	4,358 (3,751 to 5,063)	15.8 (14.1 to 17.7)	1
Haryana	5,316 (4,527 to 6,136)	15.6 (14.2 to 17.3)	1
**High SDI**	**3,162 (2,748 to 3,674)**	**10.0 (9.3 to 10.8)**	**3**
Uttarakhand	4,050 (3,458 to 4,733)	12.3 (11.0 to 13.8)	1
Tamil Nadu	2,501 (2,053 to 3,006)	7.4 (6.5 to 8.4)	5
Mizoram	4,619 (3,898 to 5,420)	15.1 (13.8 to 16.3)	1
Maharashtra	3,474 (2,961 to 4,077)	11.2 (10.0 to 12.4)	2
Punjab	3,911 (3,350 to 4,559)	12.1 (10.9 to 13.4)	3
Sikkim	2,643 (2,204 to 3,208)	10.0 (9.2 to 10.9)	1
Nagaland	5,804 (4,714 to 7,169)	18.9 (17.0 to 20.9)	1
Himachal Pradesh	3,774 (3,170 to 4,471)	11.7 (10.4 to 13.3)	1
Union Territories other than Delhi	3,981 (3,389 to 4,717)	13.9 (12.6 to 15.3)	1
Kerala	1,618 (1,289 to 2,010)	5.3 (4.6 to 6.2)	8
Delhi	4,393 (3,680 to 5,199)	15.8 (13.9 to 17.8)	1
Goa	2,221 (1,755 to 2,765)	7.9 (7.0 to 8.8)	5

DALY=disability-adjusted life-year. SDI=Socio-demographic Index. *The states are listed in increasing order of Socio-demographic Index in 2017.
†The state of Jammu and Kashmir was divided into two union territories in August 2019; as we are reporting findings up to 2017, we report findings for the state of Jammu and Kashmir.

6. Percentage contribution of DALYs and deaths attributable to child and maternal malnutrition and its components by sex in India, 2017

	Percentage of total DALYs in under-5 (95% uncertainty interval)			Percentage of total deaths in under-5 (95% uncertainty interval)		
	Boys	Girls	Both sexes	Boys	Girls	Both sexes
**Child and maternal malnutrition**	**67.9 (65.4 to 70.3)**	**66.3 (63.8 to 69.2)**	**67.1 (64.9 to 69.4)**	**69.1 (66.4 to 71.6)**	**67.3 (64.6 to 70.3)**	**68.2 (65.8 to 70.7)**
**Low birth weight and short gestation**	**46.5 (44.5 to 48.4)**	**40.6 (38.8 to 42.3)**	**43.6 (41.8 to 45.2)**	**49.4 (47.7 to 51.1)**	**42.7 (41.1 to 44.3)**	**46.1 (44.5 to 47.6)**
Short gestation for birth weight	36.8 (34.8 to 38.7)	32.1 (30.3 to 34)	34.5 (32.8 to 36.2)	39.0 (37.1 to 40.9)	33.6 (31.9 to 35.5)	36.3 (34.7 to 37.9)
Low birth weight for gestation	26.0 (24.0 to 28.2)	22.4 (20.5 to 24.7)	24.2 (22.5 to 26.2)	27.4 (25.2 to 29.6)	23.3 (21.2 to 25.6)	25.3 (23.6 to 27.2)
**Child growth failure**	**18.6 (16.6 to 20.4)**	**22.9 (20.9 to 24.9)**	**20.7 (19.0 to 22.5)**	**19.1 (17.0 to 20.9)**	**23.7 (21.6 to 25.9)**	**21.4 (19.5 to 23.2)**
Child stunting	3.73 (1.78 to 6.61)	4.45 (2.01 to 8.13)	4.09 (1.88 to 7.44)	4.01 (1.90 to 7.11)	4.76 (2.15 to 8.77)	4.38 (2.04 to 8.03)
Child wasting	17.0 (14.4 to 19.2)	21.0 (18.0 to 23.7)	19.0 (16.2 to 21.2)	17.3 (14.5 to 19.7)	21.7 (18.4 to 24.5)	19.5 (16.5 to 21.8)
Child underweight	6.31 (5.18 to 8.02)	8.01 (6.69 to 9.95)	7.16 (6.01 to 8.97)	5.92 (4.71 to 7.79)	7.82 (6.38 to 9.92)	6.87 (5.64 to 8.81)
**Suboptimal breastfeeding**	**2.88 (2.09 to 3.68)**	**3.39 (2.55 to 4.31)**	**3.13 (2.35 to 3.90)**	**3.09 (2.25 to 3.94)**	**3.09 (2.25 to 3.94)**	**3.35 (2.52 to 4.17)**
Non-exclusive breastfeeding	2.77 (2.04 to 3.56)	3.26 (2.43 to 4.17)	3.01 (2.27 to 3.78)	2.98 (2.18 to 3.82)	3.48 (2.59 to 4.44)	3.23 (2.43 to 4.04)
Discontinued breastfeeding	0.13 (0.04 to 0.25)	0.16 (0.05 to 0.29)	0.15 (0.05 to 0.27)	0.14 (0.45 to 0.26)	0.17 (0.06 to 0.30)	0.17 (0.06 to 0.30)
**Iron deficiency**	**1.34 (0.91 to 1.91)**	**0.98 (0.67 to 1.43)**	**1.16 (0.79 to 1.66)**	-	-	-
**Vitamin A deficiency**	**5.57 (4.29 to 6.91)**	**6.30 (4.91 to 7.83)**	**5.93 (4.66 to 7.31)**	**4.97 (3.67 to 6.37)**	**5.62 (4.20 to 7.15)**	**5.29 (4.02 to 6.69)**
**Zinc deficiency**	**0.33 (0.03 to 0.93)**	**0.46 (0.03 to 1.31)**	**0.39 (0.03 to 1.11)**	**0.35 (0.02 to 1.01)**	**0.48 (0.02 to 1.41)**	**0.42 (0.02 to 1.20)**

The sum of percentage DALYs attributable to the components of malnutrition is more than the total for overall malnutrition due to overlap of their contribution to disease burden, and also because the population attributable fractions from component risk factors can add up to more than the population attributable fraction for the parent risk factor even if the components are independent.
DALY=disability-adjusted life-year.

7. Projected prevalence and the NNM targets in 2022 for states of India

States of India*	Low birth weight: Projected prevalence per 100 (95% uncertainty interval) 2022†	Low birth weight: NNM 2022 target‡	Child stunting: Projected prevalence per 100 (95% uncertainty interval) 2022†	Child stunting: NNM 2022 target‡	Child underweight: Projected prevalence per 100 (95% uncertainty interval) 2022†	Child underweight: NNM 2022 target‡	Child anaemia: Projected prevalence per 100 (95% uncertainty interval) 2022†	Child anaemia: NNM 2022 target‡	Anaemia in women: Projected prevalence per 100 (95% uncertainty interval) 2022†	Anaemia in women: NNM 2022 target‡
**India**	**20.3 (19.6 to 20.9)**	**11.4**	**34.6 (33.7 to 35.7)**	**25.0**	**27.5 (27.0 to 28.0)**	**22.7**	**56.4 (53.1 to 60.2)**	**44.7**	**53.2 (52.1 to 54.2)**	**39.4**
Bihar	22.2 (19.7 to 24.0)	13.4	44.4 (40.6 to 50.5)	25.0	32.9 (30.9 to 35.4)	29.1	62.5 (53.8 to 72.8)	50.3	60.7 (56.5 to 65.3)	46.2
Madhya Pradesh	22.3 (19.7 to 24.2)	13.2	35.5 (33.1 to 38.2)	25.0	29.2 (27.7 to 31.3)	27.1	64.5 (58.5 to 71.2)	52.9	52.4 (48.8 to 56.1)	41.6
Jharkhand	19.2 (17.4 to 20.5)	10.2	42.5 (39.2 to 46.7)	25.0	36.1 (33.7 to 39.5)	32.2	72.7 (65.9 to 80.6)	57.1	63.2 (59.1 to 67.7)	48.7
Uttar Pradesh	23.3 (20.7 to 25.2)	14.2	44.4 (41.1 to 49.1)	25.0	31.3 (29.6 to 33.2)	26.4	65.5 (58.8 to 72.8)	51.7	53.7 (49.9 to 57.3)	39.6
Rajasthan	21.6 (19.2 to 23.4)	12.6	32.2 (30.0 to 34.5)	25.0	27.8 (26.4 to 29.5)	23.2	55.8 (48.8 to 63.4)	44.5	47.0 (43.1 to 51.8)	33.7
Chhattisgarh	15.7 (13.6 to 17.5)	7.1	32.7 (29.9 to 36.7)	25.0	27.3 (25.7 to 29.3)	23.4	32.7 (26.2 to 39.7)	30.7	45.3 (41.3 to 49.6)	38.1
Odisha	20.6 (18.6 to 22.1)	11.8	30.9 (28.7 to 33.5)	25.0	26.4 (24.9 to 28.0)	21.9	39.5 (33.4 to 46.6)	30.3	48.7 (43.9 to 53.6)	35.2
Assam	19.6 (17.5 to 21.0)	10.8	33.2 (30.5 to 36.8)	25.0	23.2 (21.7 to 24.6)	16.9	27.4 (22.0 to 33.7)	21.3	45.1 (40.9 to 49.4)	38.5
Andhra Pradesh	17.9 (16.3 to 19.5)	9.1	30.6 (28.4 to 32.8)	25.0	22.7 (21.5 to 24.0)	17.2	57.4 (50.2 to 65.9)	44.5	60.0 (56.3 to 63.8)	43.6
West Bengal	19.5 (17.0 to 21.2)	10.8	26.9 (25.1 to 29.2)	25.0	25.6 (24.3 to 26.9)	20.2	52.3 (43.8 to 62.4)	40.7	58.1 (53.7 to 63.4)	42.6
Tripura	19.4 (18.0 to 21.0)	10.7	25.9 (23.5 to 28.2)	25.0	25.0 (23.2 to 27.1)	19.0	45.2 (36.6 to 55.1)	35.2	52.4 (47.4 to 58.3)	38.8
Arunachal Pradesh	18.7 (16.8 to 19.9)	9.3	28.1 (25.8 to 30.8)	25.0	24.1 (22.8 to 25.5)	15.7	56.7 (49.6 to 64.7)	41.7	40.9 (36.8 to 45.0)	28.7
Meghalaya	18.4 (16.9 to 19.8)	9.5	42.3 (39.6 to 45.5)	25.0	23.8 (22.5 to 25.2)	21.2	41.1 (32.9 to 48.9)	32.0	57.0 (52.2 to 62.2)	43.7
Karnataka	17.9 (16.0 to 19.5)	9.1	30.4 (28.6 to 32.4)	25.0	27.3 (26.1 to 28.5)	21.7	57.8 (50.6 to 65.8)	44.7	45.9 (42.5 to 49.5)	31.0
Telangana	14.4 (11.2 to 16.1)	5.3	26.6 (23.8 to 29.4)	25.0	23.2 (21.9 to 24.6)	18.3	56.9 (48.3 to 66.0)	47.6	57.2 (52.8 to 61.9)	44.2
Gujarat	19.5 (18.2 to 20.7)	10.7	34.6 (32.2 to 37.2)	25.0	28.4 (27.0 to 29.9)	23.5	55.5 (48.7 to 62.6)	46.7	57.3 (53.6 to 61.1)	45.1
Manipur	15.9 (13.8 to 17.5)	7.3	28.7 (26.6 to 31.0)	25.0	14.7 (13.8 to 15.9)	6.5	25.2 (19.4 to 32.2)	14.7	28.0 (24.6 to 32.0)	14.7
Jammu and Kashmir§	18.8 (17.0 to 20.3)	10.1	26.3 (24.2 to 28.5)	25.0	14.5 (13.5 to 15.4)	7.6	52.7 (45.8 to 61.6)	38.2	48.9 (45.3 to 52.4)	33.6
Haryana	21.2 (19.7 to 22.2)	12.3	29.5 (27.3 to 31.6)	25.0	23.7 (22.5 to 25.1)	17.9	71.6 (64.8 to 79.3)	59.0	65.2 (61.1 to 69.3)	50.0
Uttarakhand	22.0 (19.8 to 23.7)	12.6	26.1 (24.3 to 27.9)	25.0	17.8 (16.8 to 18.9)	14.0	58.6 (50.3 to 68.4)	47.2	44.5 (40.5 to 48.9)	35.9
Tamil Nadu	14.2 (12.2 to 16.1)	5.4	22.1 (20.5 to 23.9)	25.0	20.0 (19.1 to 21.0)	14.6	44.3 (37.6 to 51.9)	33.0	53.2 (49.4 to 57.0)	39.0
Mizoram	8.3 (6.5 to 9.9)	0.0	25.6 (23.6 to 27.6)	25.0	15.7 (14.7 to 16.7)	7.2	15.1 (11.5 to 19.4)	6.1	24.6 (21.6 to 27.9)	13.5
Maharashtra	18.1 (16.6 to 19.1)	9.8	27.3 (25.5 to 29.2)	25.0	25.1 (24.0 to 26.3)	20.3	47.3 (41.5 to 53.9)	37.4	48.8 (45.6 to 52.3)	35.2
Punjab	18.9 (17.1 to 20.3)	10.2	25.8 (24.0 to 27.9)	25.0	19.6 (18.8 to 20.6)	12.7	50.9 (43.2 to 59.3)	42.4	53.4 (48.7 to 58.2)	41.1
Sikkim	10.7 (8.2 to 12.9)	2.2	24.3 (21.8 to 26.6)	25.0	15.0 (13.7 to 16.3)	6.8	47.9 (38.8 to 57.1)	36.8	34.3 (30.3 to 38.8)	24.6
Nagaland	17.8 (15.1 to 19.4)	8.6	27.9 (25.6 to 30.3)	25.0	18.8 (17.6 to 20.0)	10.6	24.7 (19.7 to 31.0)	14.5	28.7 (25.6 to 31.9)	17.9
Himachal Pradesh	20.6 (18.6 to 22.2)	11.5	27.4 (25.0 to 30.2)	25.0	17.2 (15.9 to 18.5)	10.8	48.9 (42.0 to 56.3)	37.9	56.1 (51.1 to 60.9)	41.2
Union Territories other than Delhi	13.7 (10.6 to 16.1)	4.0	25.5 (21.9 to 30.0)	25.0	19.1 (17.2 to 21.5)	12.0	59.0 (51.1 to 67.8)	41.6	60.5 (55.6 to 65.6)	43.5
Kerala	13.7 (10.5 to 16.0)	4.4	17.7 (16.0 to 19.4)	25.0	15.3 (14.2 to 16.4)	9.3	34.6 (28.8 to 41.4)	23.0	36.0 (32.7 to 39.9)	20.1
Delhi	22.7 (19.2 to 25.0)	13.1	27.2 (24.8 to 29.7)	25.0	22.1 (20.5 to 23.8)	15.3	68.3 (57.8 to 78.6)	55.3	58.7 (52.2 to 64.9)	43.9
Goa	18.6 (15.9 to 20.6)	9.5	18.5 (16.2 to 20.8)	25.0	16.9 (15.5 to 18.3)	8.8	46.0 (38.1 to 54.8)	31.9	31.6 (27.4 to 35.9)	18.6

* The states are listed in increasing order of Socio-demographic Index in 2017.
† In 2022 if trends up to 2017 continue.
‡The NNM targets for 2025 are: 25% for stunting, annual reduction of 2 percentage points for low birth weight and underweight from 2017, and an annual reduction of 3 percentage points from 2017 in anaemia among under-five children and women aged 15-49 years.
§The state of Jammu and Kashmir was divided into two union territories in August 2019; as we are reporting findings up to 2017, we report findings for the state of Jammu and Kashmir.

8. Projected prevalence and the WHO/UNICEF targets in 2030 for states of India

States of India*	Low birth weight: Projected prevalence per 100 (95% uncertainty interval) 2030†	Low birth weight: WNO/UNICEF 2030 targets‡	Child stunting: Projected prevalence per 100 (95% uncertainty interval) 2030†	Child stunting: WNO/UNICEF 2030 targets‡	Child wasting: Projected prevalence per 100 (95% uncertainty interval) 2030†	Child wasting: WNO/UNICEF 2030 targets‡	Anaemia in women: Projected prevalence per 100 (95% uncertainty interval) 2030†	Anaemia in women: WNO/UNICEF 2030 targets‡	Exclusive breastfeeding: Projected prevalence per 100 (95% uncertainty interval) 2030†	Exclusive breastfeeding: WNO/UNICEF 2030 targets‡	Child overweight: Projected prevalence per 100 (95% uncertainty interval) 2030†	Child overweight: WNO/UNICEF 2030 targets‡
**India**	**18.7 (17.7 to 19.5)**	**15.9**	**27.7 (26.4 to 29.2)**	**22.5**	**13.4 (13.0 to 13.8)**	**<3.0**	**51.1 (49.6 to 52.6)**	**28.3**	**59.3 (56.2 to 62.2)**	**≥70**	**17.5 (10.9 to 25.5)**	**<3.0**
Bihar	20.4 (17.1 to 22.8)	17.5	38.5 (32.6 to 47.5)	26.5	10.5 (9.2 to 11.8)	<3.0	59.9 (53.9 to 66.4)	31.2	64.3 (59.3 to 69.0)	≥70	9.0 (4.4 to 15.3)	<3.0
Madhya Pradesh	20.8 (17.6 to 23.5)	17.1	27.6 (24.4 to 31.2)	23.9	15.8 (14.4 to 17.2)	<3.0	45.7 (40.2 to 51.0)	30.9	64.8 (59.9 to 69.3)	≥70	13.7 (7.2 to 22.7)	<3.0
Jharkhand	17.6 (15.4 to 19.1)	15.1	37.5 (32.5 to 43.9)	25.0	15.5 (13.9 to 17.2)	<3.0	62.3 (56.3 to 68.7)	32.8	68.5 (64.0 to 72.6)	≥70	14.2 (6.9 to 25.1)	<3.0
Uttar Pradesh	22.0 (18.4 to 24.4)	17.6	37.3 (32.0 to 44.2)	27.3	11.9 (10.8 to 13.0)	<3.0	52.2 (46.9 to 57.4)	28.1	40.0 (35.0 to 44.9)	≥70	12.2 (6.2 to 21.1)	<3.0
Rajasthan	20.0 (17.0 to 22.3)	16.7	24.8 (21.9 to 28.0)	21.8	17.0 (15.4 to 18.5)	<3.0	44.4 (39.1 to 51.1)	26.5	63.5 (58.8 to 68.1)	≥70	12.7 (6.4 to 22.0)	<3.0
Chhattisgarh	13.7 (11.0 to 16.1)	13.3	24.3 (20.8 to 29.4)	23.2	14.3 (12.7 to 16.0)	<3.0	33.6 (28.4 to 39.5)	28.9	77.8 (74.5 to 80.8)	≥70	16.7 (8.5 to 27.3)	<3.0
Odisha	18.8 (16.2 to 20.8)	16.2	23.7 (21.0 to 27.3)	21.2	15.5 (14.0 to 17.0)	<3.0	46.3 (39.3 to 53.3)	26.6	71.2 (67.1 to 75.1)	≥70	16.1 (8.0 to 26.7)	<3.0
Assam	17.9 (15.2 to 19.9)	15.5	26.2 (22.2 to 31.2)	21.9	11.3 (9.9 to 12.9)	<3.0	32.4 (27.4 to 38.2)	30.7	70.8 (66.1 to 75.2)	≥70	15.1 (7.3 to 26.2)	<3.0
Andhra Pradesh	16.1 (14.1 to 18.2)	14.2	24.1 (21.2 to 27.1)	20.4	16.0 (14.5 to 17.7)	<3.0	62.3 (56.7 to 67.6)	29.9	70.1 (65.8 to 74.2)	≥70	28.1 (15.6 to 42.5)	<3.0
West Bengal	17.5 (14.4 to 19.6)	15.7	19.8 (17.7 to 22.8)	19.8	9.8 (8.9 to 11.0)	<3.0	58.9 (52.6 to 66.3)	29.7	58.7 (52.4 to 64.2)	≥70	25.5 (14.2 to 38.6)	<3.0
Tripura	17.6 (15.8 to 19.5)	15.5	19.6 (16.6 to 22.8)	17.5	13.4 (11.7 to 15.3)	<3.0	50.2 (42.9 to 59.3)	29.0	71.1 (65.1 to 76.2)	≥70	21.3 (10.9 to 35.1)	<3.0
Arunachal Pradesh	17.8 (15.3 to 19.7)	14.0	22.2 (18.7 to 26.1)	18.9	17.4 (15.4 to 19.6)	<3.0	36.5 (31.1 to 42.5)	24.4	65.3 (59.6 to 70.4)	≥70	28.5 (16.0 to 42.8)	<3.0
Meghalaya	16.9 (14.6 to 19.0)	14.5	38.1 (33.9 to 42.8)	24.0	8.3 (7.1 to 9.6)	<3.0	54.1 (47.2 to 61.7)	29.6	49.3 (40.2 to 57.8)	≥70	17.9 (9.0 to 31.5)	<3.0
Karnataka	16.2 (13.7 to 18.1)	14.3	24.0 (21.6 to 26.8)	20.3	15.6 (14.1 to 17.2)	<3.0	45.6 (40.8 to 50.6)	24.4	63.4 (58.6 to 68.0)	≥70	21.0 (10.9 to 33.9)	<3.0
Telangana	13.0 (9.2 to 15.4)	11.2	19.6 (16.4 to 23.1)	19.2	14.3 (12.3 to 16.4)	<3.0	54.0 (47.0 to 61.2)	31.1	71.1 (66.4 to 75.5)	≥70	38.1 (23.6 to 53.7)	<3.0
Gujarat	17.7 (16.1 to 19.3)	15.5	27.2 (24.0 to 30.9)	23.0	17.6 (16.2 to 19.0)	<3.0	52.7 (47.3 to 58.6)	31.7	60.1 (54.1 to 65.3)	≥70	20.7 (11.0 to 33.1)	<3.0
Manipur	13.9 (11.4 to 16.0)	13.3	23.6 (20.4 to 26.9)	18.2	6.6 (5.6 to 7.8)	<3.0	25.4 (20.8 to 31.1)	17.6	75.6 (71.9 to 78.9)	≥70	27.1 (15.5 to 40.5)	<3.0
Jammu and Kashmir§	16.9 (14.5 to 18.8)	15.0	20.6 (18.0 to 23.5)	17.2	6.9 (5.9 to 8.0)	<3.0	49.3 (44.1 to 54.2)	25.2	70.2 (65.5 to 74.5)	≥70	19.0 (9.5 to 32.3)	<3.0
Haryana	19.6 (17.7 to 21.0)	16.5	21.6 (18.7 to 24.7)	20.9	10.4 (9.1 to 11.8)	<3.0	65.5 (59.2 to 71.5)	32.8	60.4 (54.0 to 66.5)	≥70	24.9 (14.4 to 37.8)	<3.0
Uttarakhand	21.1 (18.3 to 23.2)	16.4	17.9 (15.8 to 20.1)	19.6	8.5 (7.3 to 9.9)	<3.0	34.7 (29.6 to 40.2)	27.1	60.9 (54.5 to 67.2)	≥70	25.1 (13.7 to 39.2)	<3.0
Tamil Nadu	12.5 (10.1 to 14.9)	11.8	17.1 (15.1 to 19.4)	15.4	15.8 (14.4 to 17.2)	<3.0	52.0 (46.8 to 57.4)	26.6	58.1 (52.5 to 63.6)	≥70	21.2 (11.2 to 34.9)	<3.0
Mizoram	7.7 (5.3 to 9.7)	6.4	19.9 (16.8 to 22.9)	17.2	13.1 (11.7 to 14.7)	<3.0	19.2 (15.7 to 23.4)	16.7	67.7 (61.3 to 73.3)	≥70	19.0 (9.5 to 30.9)	<3.0
Maharashtra	15.6 (13.9 to 17.0)	15.6	19.5 (17.3 to 22.0)	19.4	15.2 (13.8 to 16.7)	<3.0	46.7 (41.9 to 51.8)	26.0	66.9 (61.7 to 72.0)	≥70	22.8 (12.7 to 35.9)	<3.0
Punjab	17.0 (14.7 to 19.0)	15.2	19.7 (17.4 to 22.3)	18.0	9.5 (8.4 to 10.7)	<3.0	49.1 (42.3 to 56.3)	27.6	61.0 (54.5 to 67.3)	≥70	20.0 (10.4 to 33.7)	<3.0
Sikkim	8.6 (6.4 to 11.0)	10.5	18.5 (14.8 to 22.0)	16.7	5.1 (4.2 to 6.3)	<3.0	26.6 (21.8 to 32.3)	23.7	64.9 (57.0 to 71.4)	≥70	23.1 (11.9 to 39.0)	<3.0
Nagaland	16.5 (13.1 to 18.7)	13.9	22.8 (19.1 to 26.3)	18.1	10.6 (9.2 to 11.9)	<3.0	22.7 (19.1 to 26.7)	19.8	55.4 (47.3 to 62.0)	≥70	20.9 (10.7 to 35.3)	<3.0
Himachal Pradesh	19.2 (16.4 to 21.5)	15.8	20.9 (17.8 to 24.5)	18.9	9.4 (8.1 to 10.9)	<3.0	55.8 (48.7 to 62.5)	28.1	74.6 (69.0 to 79.2)	≥70	31.7 (19.7 to 46.6)	<3.0
Union Territories other than Delhi	12.3 (8.6 to 15.6)	10.8	20.0 (15.8 to 25.6)	16.5	12.1 (9.5 to 14.9)	<3.0	63.6 (56.6 to 70.6)	28.8	63.5 (57.0 to 69.5)	≥70	32.4 (17.7 to 49.8)	<3.0
Kerala	12.7 (8.6 to 15.8)	10.5	12.6 (10.7 to 14.5)	13.3	16.2 (14.7 to 17.9)	<3.0	37.4 (32.6 to 43.1)	18.4	63.7 (57.2 to 69.5)	≥70	34.4 (20.5 to 49.8)	<3.0
Delhi	22.2 (17.3 to 25.1)	16.4	20.3 (17.3 to 23.4)	19.0	10.8 (9.3 to 12.4)	<3.0	58.3 (49.1 to 66.9)	28.8	58.0 (48.8 to 65.5)	≥70	38.0 (23.4 to 54.0)	<3.0
Goa	17.3 (13.8 to 19.8)	14.3	14.5 (11.6 to 17.7)	12.0	15.7 (14.0 to 17.6)	<3.0	28.5 (23.2 to 34.4)	18.6	62.4 (53.6 to 70.0)	≥70	35.7 (20.3 to 53.6)	<3.0

* The states are listed in increasing order of Socio-demographic Index in 2017.
† In 2030 if trends up to 2017 continue.
‡The WHO/UNICEF targets for 2030 are: 30% reduction in low birth weight from 2012; 50% reduction in stunting from 2012; wasting less than 3%; 70% or more exclusive breastfeeding; 50% reduction of anaemia in reproductive age women from 2012; and child overweight less than 3%.
§The state of Jammu and Kashmir was divided into two union territories in August 2019; as we are reporting findings up to 2017, we report findings for the state of Jammu and Kashmir.

9. Diagnostic test for robustness of 1000 draws to estimate the 95% uncertainty intervals for low birth weight, stunting, and wasting

Diagnostic test of the 1000 draws distribution was conducted for all states for low birth weight, stunting, and wasting. The test was conducted via random down-sampling draws by orders of 100 in order to identify the number of draws where instability in the 95% uncertainty interval begins to be evident, i.e. starting with 1000 draws, and an iterative approach was undertaken to randomly draw 900 samples, then 800 samples, then 700 samples, etc. The results in the following plots demonstrates the robustness of the 1000 draws approach to estimate 95% uncertainty intervals, as the point at which instability in the uncertainty interval starts becoming evident is 100s of draws below the 1000 draws level.

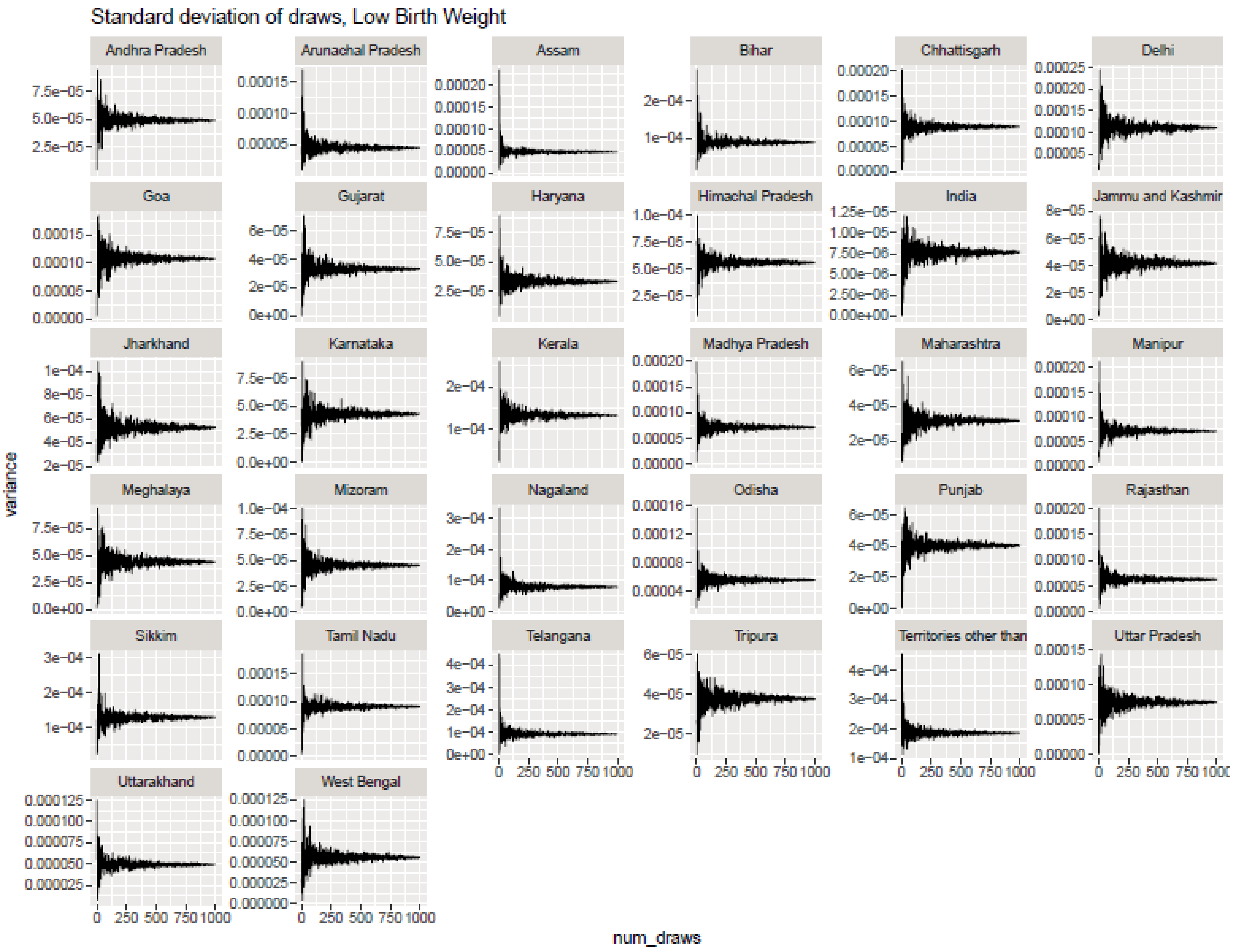

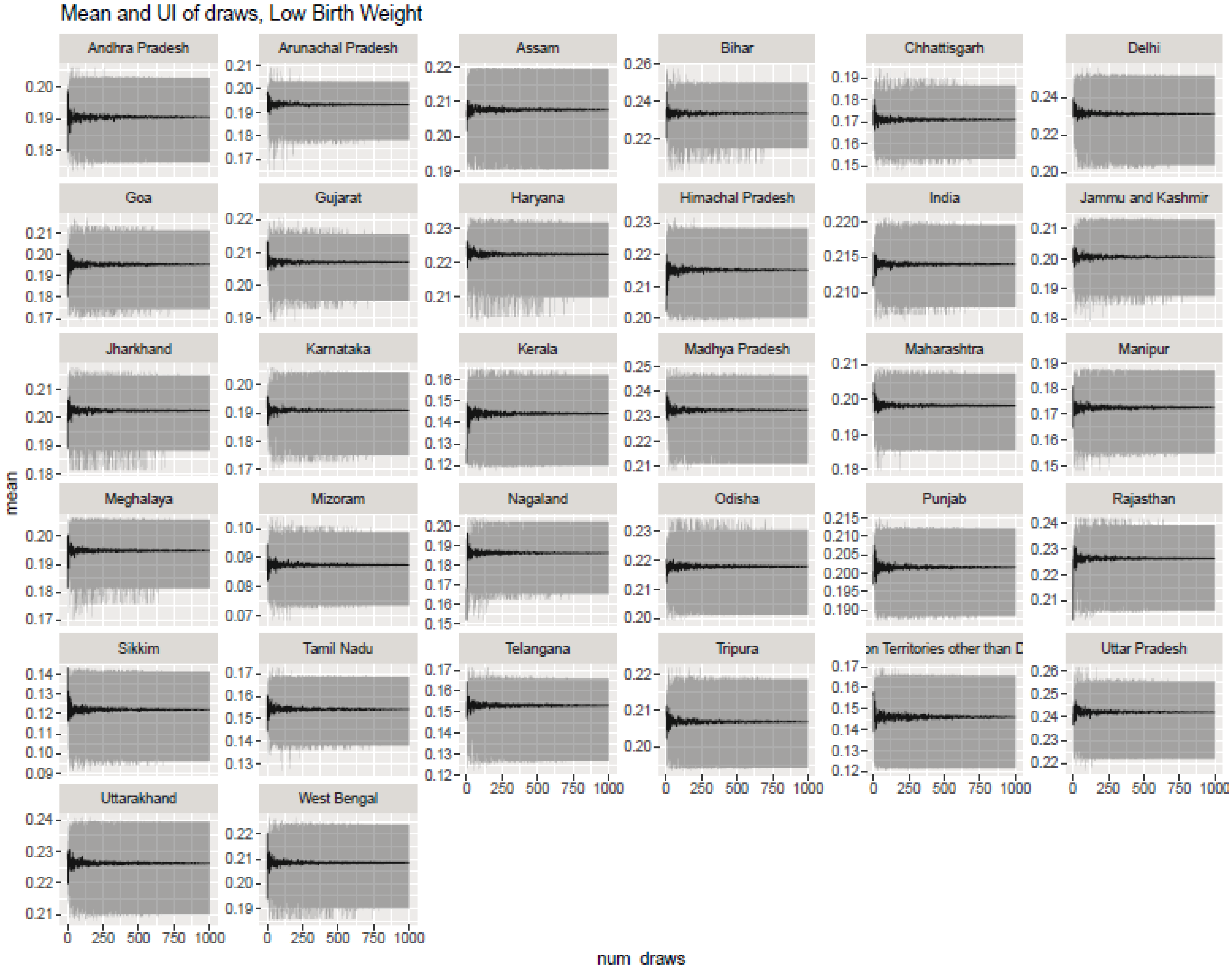

Mean and UI of draws, Low Birth Weight
Andhra Pradesh
Arunachal Pradesh
Assam
Bihar
Chhattisgarh
Delhi
Goa
Gujarat
Haryana
Himachal Pradesh
India
Jammu and Kashmir
Jharkhand
Karnataka
Kerala
Madhya Pradesh
Maharashtra
Manipur
Meghalaya
Mizoram
Nagaland
Odisha
Punjab
Rajasthan
Sikkim
Tamil Nadu
Telangana
Tripura
on Territories other than D
Uttar Pradesh
Uttarakhand
West Bengal
mean
num_draws

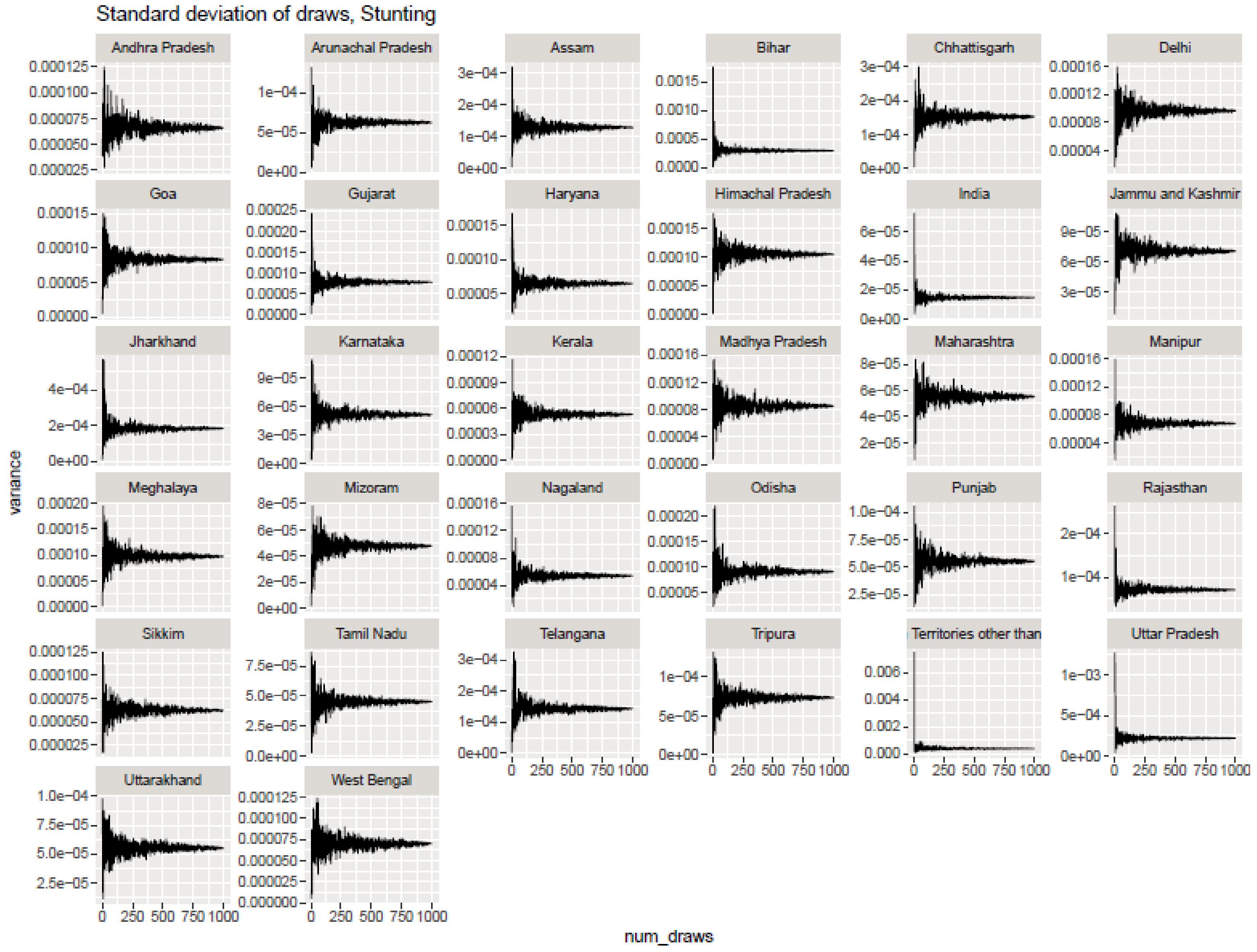
Standard deviation of draws, Stunting
Andhra Pradesh
Arunachal Pradesh
Assam
Bihar
Chhattisgarh
Delhi
Goa
Gujarat
Haryana
Himachal Pradesh
India
Jammu and Kashmir
Jharkhand
Karnataka
Kerala
Madhya Pradesh
Maharashtra
Manipur
Meghalaya
Mizoram
Nagaland
Odisha
Punjab
Rajasthan
Sikkim
Tamil Nadu
Telangana
Tripura
Territories other than
Uttar Pradesh
Uttarakhand
West Bengal
variance
num_draws

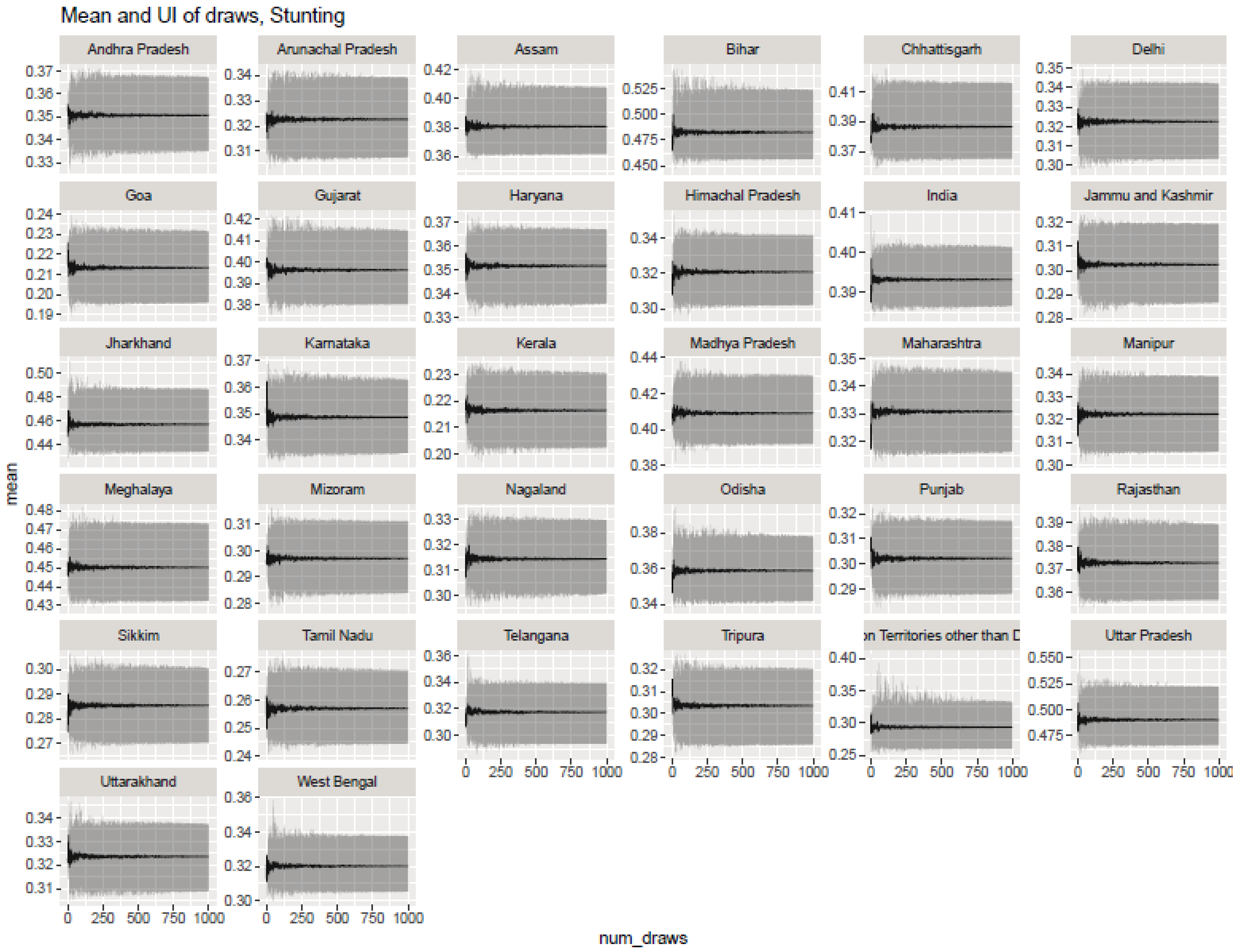
Mean and UI of draws, Stunting
Andhra Pradesh
Arunachal Pradesh
Assam
Bihar
Chhattisgarh
Delhi
Goa
Gujarat
Haryana
Himachal Pradesh
India
Jammu and Kashmir
Jharkhand
Karnataka
Kerala
Madhya Pradesh
Maharashtra
Manipur
Meghalaya
Mizoram
Nagaland
Odisha
Punjab
Rajasthan
Sikkim
Tamil Nadu
Telangana
Tripura
on Territories other than D
Uttar Pradesh
Uttarakhand
West Bengal
mean
num_draws

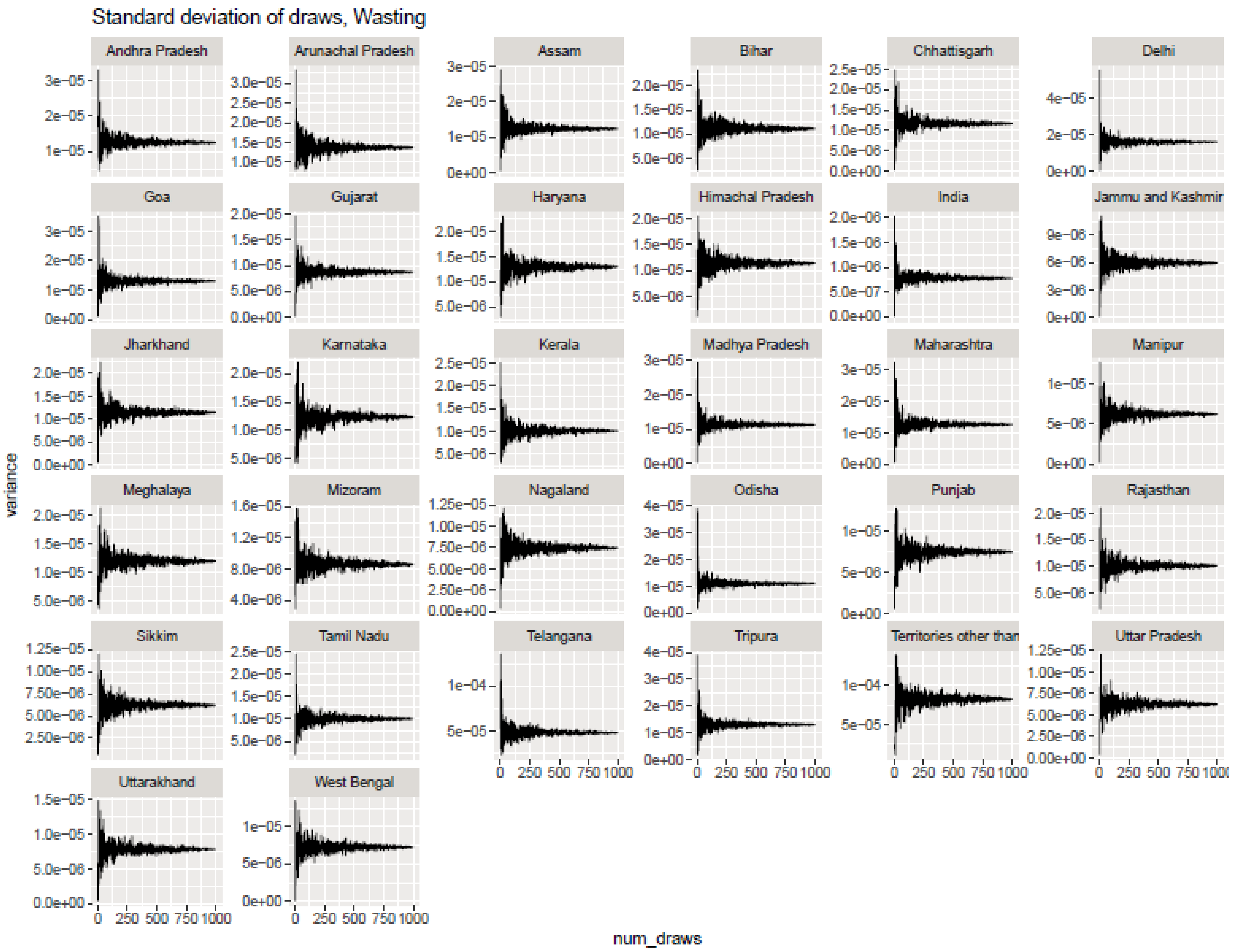

Standard deviation of draws, Wasting
Andhra Pradesh
Arunachal Pradesh
Assam
Bihar
Chhattisgarh
Delhi
Goa
Gujarat
Haryana
Himachal Pradesh
India
Jammu and Kashmir
Jharkhand
Karnataka
Kerala
Madhya Pradesh
Maharashtra
Manipur
Meghalaya
Mizoram
Nagaland
Odisha
Punjab
Rajasthan
Sikkim
Tamil Nadu
Telangana
Tripura
Territories other than
Uttar Pradesh
Uttarakhand
West Bengal
variance
num_draws

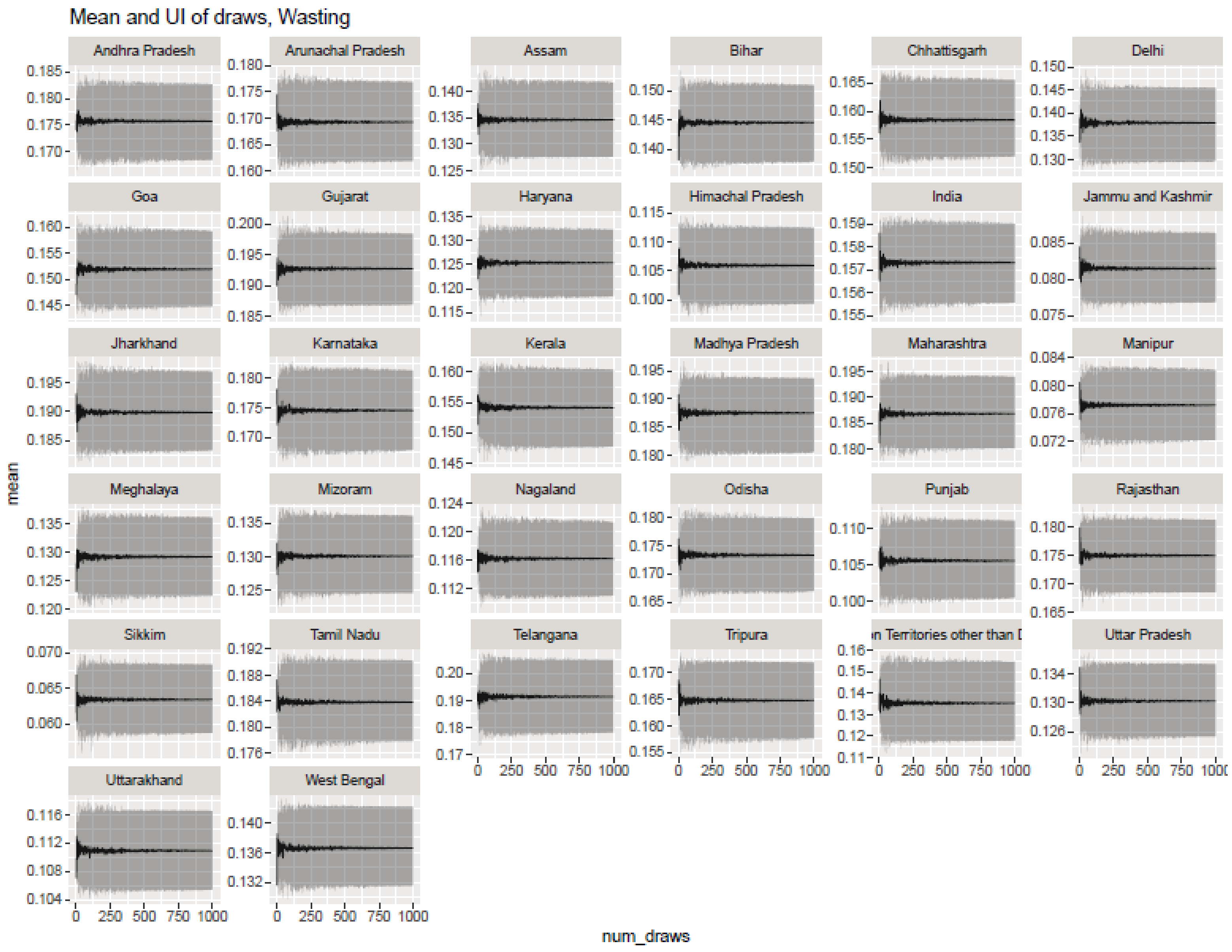
Mean and UI of draws, Wasting
Andhra Pradesh
Arunachal Pradesh
Assam
Bihar
Chhattisgarh
Delhi
Goa
Gujarat
Haryana
Himachal Pradesh
India
Jammu and Kashmir
Jharkhand
Karnataka
Kerala
Madhya Pradesh
Maharashtra
Manipur
Meghalaya
Mizoram
Nagaland
Odisha
Punjab
Rajasthan
Sikkim
Tamil Nadu
Telangana
Tripura
n Territories other than I
Uttar Pradesh
Uttarakhand
West Bengal
mean
num_draws
